# Habitat‐Based Predictions of Bridle Shiner (*Notropis bifrenatus*) in the Northeastern United States

**DOI:** 10.1002/ece3.72413

**Published:** 2026-01-12

**Authors:** Lara S. Katz, Stephen M. Coghlan, Matthew A. Carpenter, Michael T. Kinnison, Joseph D. Zydlewski

**Affiliations:** ^1^ Department of Wildlife, Fisheries, and Conservation Biology University of Maine Orono Maine USA; ^2^ New Hampshire Fish and Game Department Concord New Hampshire USA; ^3^ Maine Center for Genetics in the Environment Orono Maine USA; ^4^ U.S. Geological Survey, Maine Cooperative Fish and Wildlife Research Unit University of Maine Orono Maine USA

## Abstract

We sought to assess bridle shiner ( *Notropis bifrenatus* ) habitat associations at local and regional scales across southern Maine and New Hampshire. We used local habitat data at 95 Maine sites to predict occupancy with classification and regression trees (CART). We then used ensemble species distribution models (SDMs) to model the historical (1898–2008) and current (2009–2022) ranges of the species. We used the BIOMOD platform to model the association between 35 environmental variables and bridle shiner presence during both time periods and at fine (pseudo‐HUC14) and coarse (HUC12) spatial scales. We then calculated the change in predicted occupied drainages to estimate the change in the species' distribution at both scales. Within a site, bridle shiners were associated with submerged aquatic vegetation, organic substrate, and watermilfoil (*Myriophyllum* spp.). SDMs revealed an association with Appalachian (Hemlock‐)Northern Hardwood Forest, sand substrate, and low‐elevation terrain (at both spatial scales). Ensemble fine‐scale SDMs suggest a substantial loss of historical bridle shiner habitat in both Maine (36% of drainages) and New Hampshire (16%), with comparable described losses (of 21% and 14%) at a coarse scale. Our local and regional models may be used to focus surveys on areas with high predicted habitat suitability or to inform habitat restoration efforts.

## Graphical abstract

We developed local and regional models for Maine and New Hampshire that may be used to focus bridle shiner ( *Notropis bifrenatus* ) surveys on areas with high predicted habitat suitability. This is significant because bridle shiners receive protection or concern status in thirteen states and two Canadian provinces: Limited data show that bridle shiners have declined dramatically throughout most of the species' native range, and our results provide evidence that bridle shiners are declining in Maine and New Hampshire. In addition to guiding the search for undiscovered populations of bridle shiner, managers may also use our model results to search for suitable reintroduction sites or focus habitat restoration efforts.

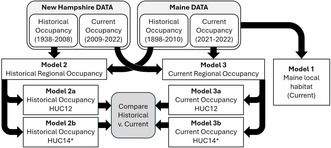

## 1 Introduction

The northern United States and southern Canadian provinces support several temperate fish species at the northern limit of their ranges (e.g., banded sunfish [ *Enneacanthus obesus* ], swamp darter [ *Etheostoma fusiforme* ], and tessellated darter [ *E. olmstedi* ]). Other boreal species are at the southern limit of their range (e.g., lake chub [ *Couesius plumbeus* ], northern pearl dace [ *Margariscus nachtriebi* ], and finescale dace [ *Chrosomus neogaeus* ]). Many of these species are locally at risk of extirpation despite being common elsewhere in their range (Gibson et al. 2009; Booher and Walters 2021). Monitoring these peripheral populations is critical to their conservation as rising temperatures shift biotic communities poleward (Viana 2017). Populations at the latitudinal edge of a species' range may be more isolated than at the core (Haak et al. 2010; Lamothe and Drake 2020) and utilize different habitats (Haak et al. 2010; Lamothe and Drake 2020). As a result, species with poor dispersal ability and short generation times may be likely to have important local adaptations (Lesica and Allendorf 1995).

Monitoring rare or declining species is challenging, and detection probabilities may be extremely low (Guisan et al. 2006). One approach to detecting such species is to conduct targeted surveys based on species distribution models (SDM). SDMs statistically associate occurrence data with environmental variables so that areas with suitable habitat may be identified (Riaz et al. 2020). These spatially explicit habitat models improve survey efficiency when compared with simple or stratified random sampling (Guisan et al. 2006). Model predictions, however, may be highly variable, and the choice of method impacts model outcomes and accuracy (Araújo and New 2007). Ensemble modeling approaches that combine model predictions may produce more robust predictions (Marmion et al. 2009).

Significant logistical challenges arise for modeling aquatic species. SDM platforms (such as BIOMOD [Thuiller et al. 2009] and SDMtune [Vignali et al. 2020]) work by comparing environmental variables at “presence” points to those at “absence” or “pseudo‐absence” points that must be generated from aquatic habitats. Aquatic species, however, are not only influenced by environmental factors at a point but also by characteristics of the watershed. For example, the cumulative upstream effects of anthropogenic and land cover factors may be highly predictive of stream fish distribution (Markovic et al. 2019). Because detailed hydrologic data are often unavailable at small scales, freshwater SDMs must often compromise and make use of topographic, climatic, and land cover variables aggregated by drainage (Domisch et al. 2015; Kärcher et al. 2019). These modeling units are irregular and nonrandomly distributed, and their size and configuration are dependent upon topography and geomorphometry (Amatulli et al. 2018; Friedrichs‐Manthey et al. 2020). This is important to consider as patterns of predictor importance may vary across species and drainage scale (e.g., temperature; Kärcher et al. 2019).

Friedrichs‐Manthey et al. (2020) encourage building freshwater SDMs across multiple drainage sizes to account for the “modifiable area unit problem” (MAUP). The problem arises because SDM predictions are highly dependent upon the size of the spatial unit used to aggregate data (Jelinski and Wu 1996; Lobo et al. 2008; Connor et al. 2018). Conclusions drawn from SDMs (e.g., covariate importance, habitat suitability, range size) are therefore limited to a specific spatial resolution (Seo et al. 2008; Connor et al. 2018; Friedrichs‐Manthey et al. 2020). With these challenges in mind, we sought to assess the habitat use of a rare minnow species, the bridle shiner ( *Notropis bifrenatus* ).

**FIGURE 1:**
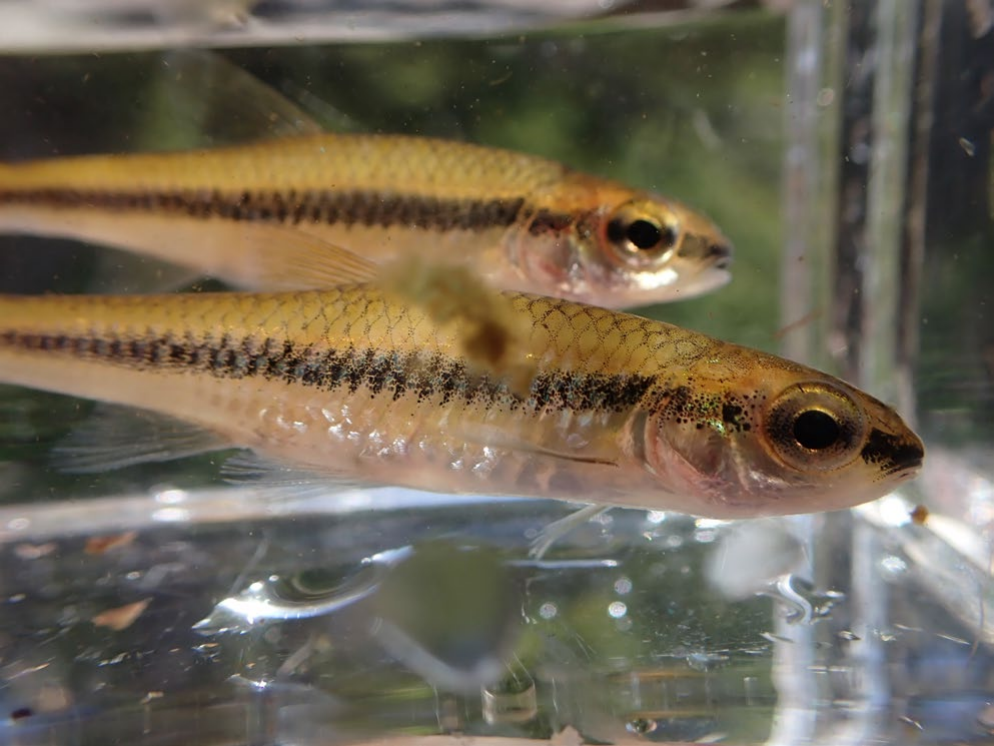
Two bridle shiners ( *Notropis bifrenatus* ) captured via seine net at Highland Lake, Cumberland County, Maine, in 2021.

The bridle shiner is a small‐bodied, specialist minnow native to the eastern United States and Canada (Figure 1). Historically, Maine's Saco River basin marked the northeastern limit of its known range in the United States. While the bridle shiner is thought to be declining throughout most of its native range, data are limited. The species is presumed to be extirpated from Maryland (Kilian et al. 2011) and South Carolina (Geneva et al. 2018; Starnes et al. 2025). In Delaware, New Jersey, and Pennsylvania, populations have declined as urbanization has increased (Cooper 1985). There are few known bridle shiner populations left in Virginia and North Carolina, where the species has likely been extirpated from the majority of its former range (Geneva et al. 2018; Starnes et al. 2025). This species now receives legal protection or “concern status” in thirteen states and two provinces (COSEWIC 2013; Hammerson 2021). Bridle shiners are listed as “threatened” in New Hampshire (NHFGD 2015) and as a “species of special concern” in Maine (MDIFW 2021).

Specific to our species of interest is a clear association with dense beds of aquatic vegetation in lakes, ponds, and rivers (Harrington 1948a). Submerged and emergent aquatic plants support diverse invertebrate communities and may serve bridle shiners and other species as foraging habitat, nurseries, and refuge from predators (Rozas and Odum 1988; Strayer and Malcom 2007; Wilson and Ricciardi 2009). Bridle shiners and other littoral fish may directly consume vascular plant material or epiphytic algae (Harrington 1948b; Carpenter and Lodge 1986). As bridle shiners are linked to aquatic plants, the species may be vulnerable to the loss or alteration of aquatic plant habitat (Pregler et al. 2019). Bridle shiners are highly sensitive to the changes in water quality, turbidity, and plant cover that result from anthropogenic disturbance (Cooper 1985; Gray et al. 2016). We have considered this knowledge as a starting point for our study approach.

The two objectives of our study were to (1) assess bridle shiner habitat associations within a local site, and to (2) inform bridle shiner conservation at the regional scale by modeling their distribution across southern Maine and New Hampshire. Recent bridle shiner surveys in Maine (2021–2022; Katz et al. 2024) and New Hampshire presented a unique opportunity to assess habitat associations at both a local and regional scale in the northeasternmost part of their range. We used an ensemble SDM approach at two spatial resolutions to characterize both the historical (1898–2008) and current (2009–2022) ranges of the bridle shiner in this region.

## 2 Methods

### 2.1 Study Area

The historical range of bridle shiners in New Hampshire and Maine falls within the Saco and Merrimack Hydrological Unit Code 6 (HUC6) basins. Much of this area was formerly glaciated, and most of the lakes were formed by glacier retreat (Wiken et al. 2011; Deeds et al. 2020). The region is dominated by mixed hardwood and spruce‐fir forests and is transitional between the northern boreal forests and the deciduous forests of New England (Wiken et al. 2011). Waterbodies along the coast are influenced by marine‐derived sediments known as the Presumpscot Formation, and coastal Maine contains much of the State's agriculture and human population because of this (Deeds et al. 2020). This is notable as agriculture and land development lead to increased erosion, nutrient loading, and road salt, which may all be significant stressors on freshwater ecosystem health (Soranno et al. 2015; Sutherland et al. 2018; Deeds et al. 2020).

### 2.2 Local Habitat Variables (Model 1)

#### 2.2.1 Maine Bridle Shiner Surveys

**FIGURE 2:**
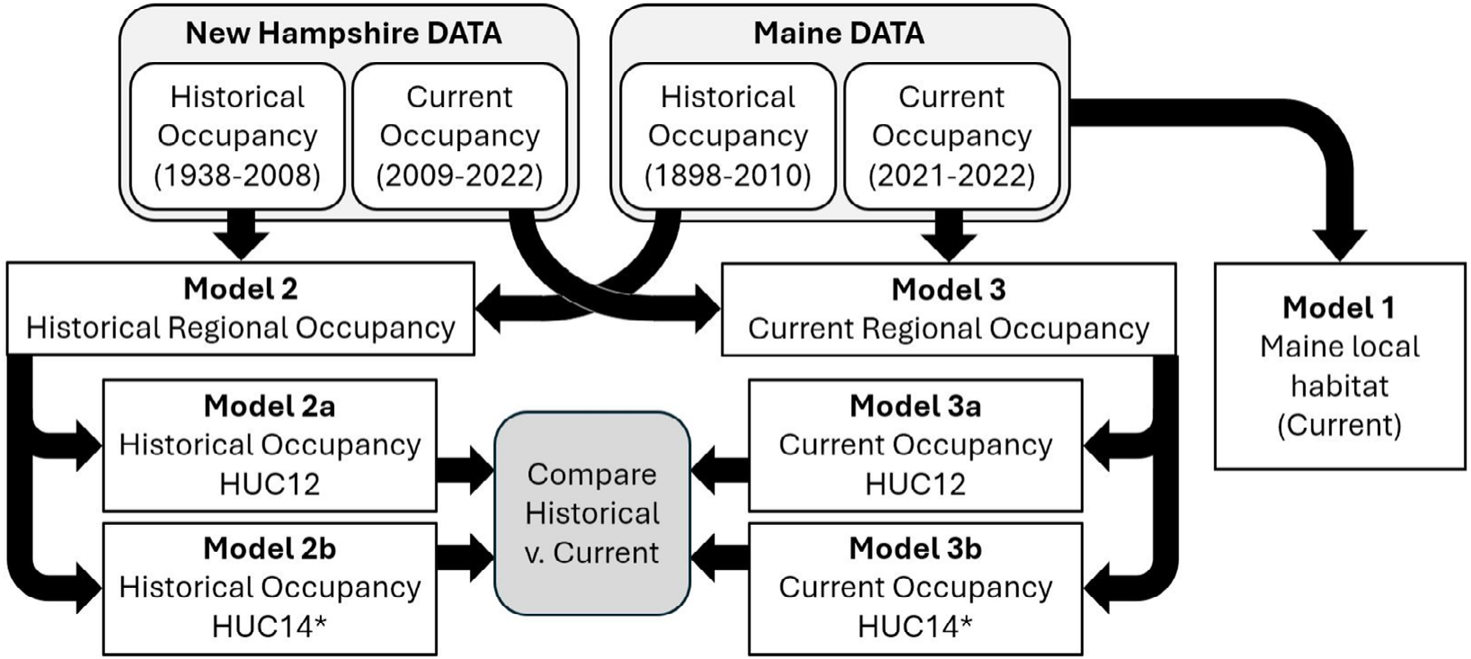
Flowchart describing the habitat models fit to bridle shiner ( *Notropis bifrenatus* ) occupancy data from Maine and New Hampshire. Habitat surveys conducted in Maine (2021–2022) were used to predict local habitat associations (Model 1). Occupancy data from Maine and New Hampshire were then used to fit species distribution models at two temporal scales (“Historical” [1898–2008; Model 2] and “Current” [2009–2022; Model 3]) and two spatial scales (“HUC12” [Models 2a, 3a] and “HUC14” [Models 2b, 3b]). “HUC12” polygons were established Hydrologic Unit Code (HUC) 12 drainages, and “HUC14” polygons were generated for this study and are an approximation of established HUC14 drainages (Supporting Information E).

We surveyed populations of Maine bridle shiners using seine netting and environmental DNA (eDNA) over the summer and fall of 2021 and 2022 (Table SA1; Figure 2). eDNA and seine netting protocols are described in Katz et al. (2024). The University of Maine Cooperative Research Extension (CORE) eDNA Laboratory designed species‐specific qPCR primers and a TaqMan minor groove binder (MGB) probe to target the cytochrome *b* mitochondrial gene region. The forward primer (5′‐TTCACTCCAGCGAACCCC‐3′) and the reverse primer (5′‐GGGACTACTAACAGTACTAGGATACTG‐3′) produced an amplicon of 149 base pairs (bp). The TaqMan MGB probe sequence was 5′‐GCCACCACACATCCAACCT‐3′ (Katz et al. 2024).

Prior to collecting eDNA samples, we surveyed aerial imagery from waterbodies where bridle shiners had been reported between 1898 and 2010 (Kendall 1914; Cooper 1939; Doering et al. 1995; Yoder et al. 2009, 2010; Gallagher 2010a, 2010b; USEPA 2016). We chose water collection sites based on historical records or descriptions of suitable habitat found in the literature (Jensen and Vokoun 2013; Pregler et al. 2015, 2019). We sampled eDNA at 49 sites within 32 waterbodies in 2021 and 2022 (Katz et al. 2024).

In 2021, we focused on sites that were likely occupied for physical collection. We collected eDNA samples and seined at 43 sites within 29 waterbodies with a historical record of occurrence (Table SA1). We collected eDNA samples at an additional seven sites within four historically occupied waterbodies in 2021 and 2022 but were unable to pair these with seine surveys. In 2022, we focused on sampling sites that would be used to inform an occupancy model. We did this by applying a rudimentary habitat suitability index model (not presented here) based on published bridle shiner habitat preferences (Katz et al. 2024). Using this model, we selected and surveyed 45 new sites in 37 waterbodies with unknown bridle shiner presence (Table SA1). Our site selection was intended (1) to reduce spatial autocorrelation between surveys, (2) to ensure that surveys covered a wide area of southern Maine, and (3) to include a wide range of habitats to better inform habitat and species distribution models.

**FIGURE 3:**
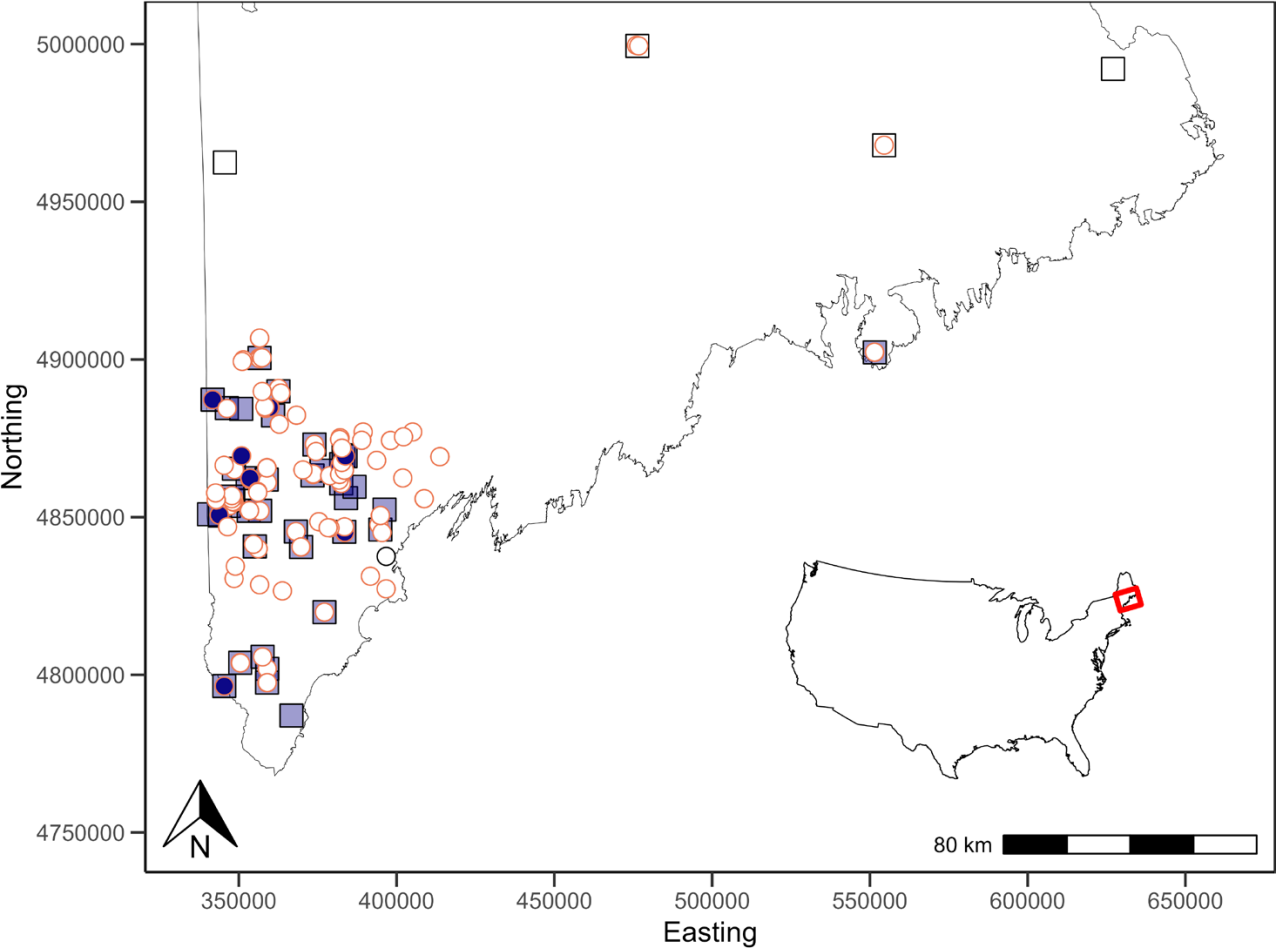
Bridle shiner ( *Notropis bifrenatus* ) survey sites in Maine. Squares signify historically occupied sites (1898–2010): White squares signify records that are likely misidentifications (see Katz et al. 2024), and transparent purple squares represent credible records. Circles signify sites surveyed in 2021–2022: White circles represent sites where bridle shiners were not detected, and purple circles represent sites where bridle shiners were detected. Circles with an orange border represent sites where we collected local habitat data.

We detected bridle shiners at 17 out of the 95 sites surveyed in Maine using seine netting, eDNA, or both methods (Table SA1; Figure 3; Katz et al. 2024). Our eDNA survey efforts gave us sufficient power (> 0.80) to detect bridle shiners at the site scale, so most of our non‐detections likely reflected true absences of the species from a survey site. However, we may have failed to detect bridle shiners at the waterbody scale due to the infeasibility of sampling entire lakes and ponds (Katz et al. 2024). The low ratio of presences to absences was partially due to the rarity of bridle shiners in Maine (including at historically occupied sites), as well as our need to sample putatively unsuitable habitats (e.g., rocky streams) for local habitat and species distribution modeling.

#### 2.2.2 Habitat Data Collection

To assess associations between aquatic plants and bridle shiner occurrence, we measured vegetation and water quality characteristics at the 95 sites sampled in Maine (2021–2022). We measured temperature (°C), total dissolved solids (ppm), and conductivity (μS/cm) using a pocket water quality tester (Oakton CTSTester 5 Waterproof Pocket Tester, Environmental Express, Charleston, South Carolina, USA) at each sampled site. At each seine site (2021), we assessed dominant substrate type (visually or by sediment particle diameter; Table 1) and dominant plant species. We collected and/or identified submerged, emergent, and floating plant species at each site. We measured water depth at three locations within each seine net haul (cm).

**TABLE 1:** Covariates used to determine local habitat associations with bridle shiner ( *Notropis bifrenatus* ) presence in Maine.

Category	Variable name	Description	Source
Location	catchment	Catchment position: 6 size classes^a^	1
	dams	Number of dams within 2‐km of sampling location	State dam point locations (2, 3, 4, 5)
	WBType	LakePond or StreamRiver	Categorized by sampling location
	elevation	Elevation (m) at survey points	6
Water chemistry	conductivity	Conductivity (μS/cm)	Measured at site
	TDS	Total dissolved solids (ppm)	Measured at site
Substrate	dom.substrate	Dominant substrate at the site (categorical)^b^	Recorded at site
	prop.org.sub	Proportion of site dominated by organic substrates	7
	prop.large.sub	Proportion of site dominated by large, inorganic substrates (> 2‐mm diameter)	7
	prop.sm.sub	Proportion of site dominated by small, inorganic substrates (< 2‐mm diameter)	7
Plant cover proportion	subm	Proportion of site dominated by submerged aquatic vegetation (SAV)	7
	emerg	Proportion of site dominated by emergent aquatic vegetation	7
	float	Proportion of site dominated by floating aquatic vegetation	7
	open	Proportion of site dominated by open water (no vegetation)	7
	subm.complex	Proportion of SAV dominated by complex‐leaved species	7
	subm.simple	Proportion of SAV dominated by simple‐leaved species	7
	subm.grasslike	Proportion of SAV dominated by mat‐forming or grass‐like species	7
	emerg.persist	Proportion of emergent vegetation dominated by persistent emergent species	7
	emerg.cattail	Proportion of emergent vegetation dominated by cattails	7
	emerg.broad	Proportion of emergent vegetation dominated by broad‐leaved deciduous species	7
	canopy	Percent tree canopy cover	8
Plant species presence/absence	ALNINC^c^	Presence of *Alnus incana* (gray alder) at site	9
	BIDBEC	Presence of *Bidens beckii* (Beck's water‐marigold) at site	9
	BRASCH	Presence of *Brasenia schreberi* (watershield) at site	9
	CALLSP	Presence of *Callitriche* spp. (water‐starworts) at site	9
	CARESP^c^	Presence of *Carex* spp. (sedges) at site	9
	CEPOCC^c^	Presence of *Cephalanthus occidentalis* (buttonbush) at site	9
	CERASP	Presence of *Ceratophyllum* spp. ( *C. demersum* or *C. echinatum* ; hornworts) at site	9
	DULARU^c^	Presence of *Dulichium arundinaceum* (threeway sedge) at site	9
	ELATRI	Presence of *Elatine triandra* (longstem waterwort) at site	9
	ELEACI	Presence of *Eleocharis acicularis* (needle spikesedge) at site	9
	ELEROB	Presence of *Eleocharis robbinsii* (Robbins' spikerush) at site	9
	ELODSP	Presence of *Elodea* spp. (waterweeds) at site	9
	ERIOSP	Presence of *Eriocaulon* spp. (pipeworts) at site	9
	EQUISP^c^	Presence of *Equisetum* spp. (horsetails) at site	9
	GRAAUR^c^	Presence of *Gratiola aurea* (golden hedge‐hyssop) at site	9
	GRASS^c^	Presence of grass spp. (including *Leersia oryzoides* , *Glyceria* spp., etc.) at site	9
	ISOESP	Presence of *Isoetes* spp. (quillworts) at site	9
	JUNCSP^c^	Presence of *Juncus* spp. ( *J. canadensis* or *J. effusus* ; Canadian or lamp rush) at site	9
	JUNMIL	Presence of *Juncus militaris* (bayonet rush) at site	9
	ILEVER^c^	Presence of *Ilex verticillata* (common winterberry) at site	9
	LEMNSP	Presence of *Lemna* spp. (duckweeds) at site	9
	LUDPAL	Presence of *Ludwigia palustris* (marsh primrose‐willow) at site	9
	LYSTER^c^	Presence of *Lysimachia terrestris* (swamp candles) at site	9
	MOSS1	Presence of Bryophyta sp. 2 (moss sp.) at site	9
	MOSS2	Presence of Bryophyta sp. 1 (moss sp.) at site	9
	MYRTEN	Presence of *Myriophyllum tenellum* (slender watermilfoil) at site	9
	MYRISP	Presence of *Myriophyllum* spp. (including *M. heterophyllum* ; watermilfoils) at site	9
	NAJASP	Presence of *Najas* spp. (waternymphs) at site	9
	NASOFF	Presence of *Nasturtium officinale* (watercress) at site	9
	NUPVAR	Presence of *Nuphar variegata* (varigated yellow pond‐lily) at site	9
	NYMCOR	Presence of *Nymphoides cordata* (little floatingheart) at site	9
	NYMODO	Presence of *Nymphaea odorata* (white waterlily) at site	9
	PERSSP^c^	Presence of *Persicaria* spp. (smartweeds) at site	9
	PONCOR	Presence of *Pontederia cordata* (pickerelweed) at site	9
	POTAMP	Presence of *Potamogeton amplifolius* (largeleaf pondweed) at site	9
	POTEPI	Presence of *Potamogeton epihydrus* (ribbonleaf pondweed) at site	9
	POTGEM	Presence of *Potamogeton pusillus* (small pondweed) at site	9
	POTILL	Presence of *Potamogeton illinoensis* (Illinois pondweed) at site	9
	POTNAT	Presence of *Potamogeton natans* (floating pondweed) at site	9
	POTOAK	Presence of *Potamogeton oakesianus* (Oakes' pondweed) at site	9
	POTPER	Presence of *Potamogeton perfoliatus* (claspingleaf pondweed) at site	9
	POTROB	Presence of *Potamogeton robbinsii* (Robbins' pondweed) at site	9
	SAGFIL	Presence of *Sagittaria filiformis* (threadleaf arrowhead) at site	9
	SAGLAT^c^	Presence of *Sagittaria latifolia* (common arrowhead) at site	9
	SCHSUB	Presence of *Schoenoplectus subterminalis* (swaying bulrush) at site	9
	SCICYP^c^	Presence of *Scirpus cyperinus* (woolgrass) at site	9
	SPARSP	Presence of *Sparganium* spp. (bur‐reeds) at site	9
	TYPHSP^c^	Presence of *Typha* spp. ( *T. angustifolia* or *T. latifolia* ; cattails) at site	9
	UTRINT	Presence of *Utricularia intermedia* (flatleaf bladderwort) at site	9
	UTRISP	Presence of *Utricularia* sp. (including *Utricularia purpurea* ; bladderworts) at site	9
	VACCSP^c^	Presence of *Vaccinium* spp. (cranberries) at site	9
	VALAME	Presence of *Vallisneria americana* (watercelery) at site	9
	ZANPAL	Presence of *Zannichellia palustris* (horned pondweed) at site	9

^a^ Northeast Aquatic Habitat Classification System (NAHCS) stream orders: 1a: Headwater: 0 < 3.861 sq.mi, 1b: Creek: ≥ 3.861 < 38.61 sq.mi., 2: Small River: ≥ 38.61 < 200 sq.mi., 3a: Medium Tributary River: ≥ 200 < 1000 sq.mi., 3b: Medium Mainstem River: ≥ 1000 < 3861 sq.mi., 4: Large River: ≥ 3861 < 9653 sq.mi., 5: Great River: ≥ 9653 sq.mi.

^b^ L: Ledge (> 1 m diameter), B: Submerged boulders or rocks (> 50 cm diameter), R: Rubble or rocks from 10 to 20″ (25–50 cm), O: Cobble rocks 2.5–10″ (6.3–25 cm); G‐Gravel 0.6–2.5″ (1.5–6.3 cm), P: Pea gravel (7.6–15 mm), S: Sand or granular material < 1/16″ (0.0625–2 mm), F: Silt or fine powdery inorganic material, M: Mud or decomposed organic material, D: Detritus or large particles of organic material such as leaves, bark, sawdust, etc.

^c^ At least partially submerged and available as cover.

*Source:* (1) NAHCS (Olivero and Anderson 2008); (2) (Massachusetts Office of Dam Safety [MAODS] and Massachusetts Dept. of Conservation and Recreation [MADCR] 2012); (3) (Maine Dept. of Environmental Protection [MEDEP] 2022); (4) (Vermont Dept. of Environmental Conservation [VTDEC] 2022); (5) (New Hampshire Dept. of Environmental Services [NHDES] 2022); (6) Digital Elevation Model (USGS 1998); (7) Estimated at site (2022) or from site photos (this study: 2021); (8) NLCD 2016 Tree Canopy Cover (USFS 2023); (9) Site survey(s), this study.

In 2022, we recorded habitat information at each site where we collected eDNA samples. We visually estimated the proportion of floating, emergent, and submerged aquatic vegetation (SAV) and the proportion of open water at each site. We divided plant species into categories (*sensu* Nohner and Diana 2015) and estimated the proportion of total SAV made up of simple‐leaved (i.e., leaves that are not divided or branched), complex‐leaved (i.e., leaves that are highly divided and feather‐like), and mat‐forming and grass‐like plants (e.g., swaying bulrush [ *Schoenoplectus subterminalis* ] and watercelery [ *Vallisneria americana* ]). We then estimated the proportion of total emergent vegetation composed of persistent vegetation (i.e., grasses and sedges), broad‐leaved deciduous vegetation (e.g., pickerelweed [ *Pontederia cordata* ]), and cattails (*Typha* spp.; Table 1).

We categorized available plant cover by plant growth form at a site rather than assigning a set subcategory to each species. For example, we classified golden hedge‐hyssop ( *Gratiola aurea* ) as submerged simple‐leaf or as emergent broad‐leaf depending on whether it was completely submerged or only available as cover during periods of high water (Table SB1). We also classified the simple‐leaved water starworts (*Callitriche* spp.) and waterweeds (*Elodea* spp.) in the submerged complex‐leaved category because their branching stems and dense mats were structurally more like watermilfoils or bladderworts (*Utricularia* spp.).

We visually estimated the proportion of the site composed of organic substrates, small inorganic substrates (< 2‐mm), and large inorganic substrates (> 2‐mm; Lamothe and Drake 2020). We measured temperature (°C), total dissolved solids (ppm), and conductivity (μS/cm) at each site. We did not directly measure the proportion of organic, small inorganic, and large inorganic substrates or the proportion of vegetation types during sampling in 2021; rather, we used photographs of the sites and plant samples to estimate these values and substrate proportions. We also did not measure water velocity at the sites because we specifically targeted areas of low or no flow where eDNA would be more likely to accumulate (Katz et al. 2024).

#### 2.2.3 Local Habitat Modeling

We used classification and regression trees (CART) to identify local‐scale environmental variables associated with bridle shiner presence in Maine (Figure 2, Model 1). We used 74 environmental variables (Table 1) from each of the 95 sites where we collected eDNA and/or seined in 2021 and 2022 (Katz et al. 2024; Figure 3), then used the CART models to determine which variables were substantively predictive of bridle shiner presence.

We assessed the catchment position of a site using the method of Pregler et al. (2019) and data from the Northeast Aquatic Habitat Classification System (NAHCS; Olivero and Anderson 2008). We counted the number of dams within a 2‐km radius of a site (within the same HUC8 subbasin; Pregler et al. 2019). We used a 10‐m digital elevation model (DEM; USGS 1998) and the 2016 National Land Cover Database (NLCD) Tree Canopy Cover dataset (Coulston et al. 2012; USFS 2023) to estimate elevation (m) and percent canopy cover at each sampling point (seine net or eDNA). We included the measurements of conductivity (μS/cm) and total dissolved solids (ppm) that we had collected in the field along with our estimates of site substrate composition and plant cover described above and in Table 1. The remaining variables were binary presence‐absence data for 53 aquatic plants identified to species or genus (Table 1; Table SB1). We did not include water temperature because the pocket water tester was sensitive to outside air temperature, and thus water temperature data collected in summer were often inaccurate (e.g., readings > 40°C). Because we only measured water depth at sites sampled via seine net, we did not include depth in local habitat models. The majority of sampled areas were below 1.5‐m in depth and accessible via chest waders.

Although researchers may opt to remove highly correlated ($\left|r\right|$≥ 0.70; Dormann et al. 2013) variables prior to model fitting (e.g., Murphy et al. 2010; Booher and Walters 2021), we note that such attempts to circumvent multicollinearity may unintentionally mask interactions among predictor variables, thereby reducing the predictive power of local non‐parametric modeling methods. This is the case for the CART method we have used (Muñoz and Felicísimo 2004). Furthermore, CART analyses are robust to outliers, multicollinearity, instances of missing data, mixed data types, and deviance from multivariate normality (De'ath and Fabricius 2000; Borcard et al. 2018). Therefore, as a strategic approach to maximize model predictive power, we did not transform or standardize continuous variables, nor did we remove highly correlated variables in the local habitat analyses.

Because there were only 17 sites occupied by bridle shiners, splitting the data into training and testing datasets would most likely result in insufficient data to build robust models (De'ath and Fabricius 2000) and be influenced by the choice of model hyperparameters (e.g., minimum number of observations required to split a node; *minsplit*). To account for these biases, we generated 1000 random seeds and used these to randomly split local habitat data into 1000 combinations of training (60%) and testing (40%) data (De'ath and Fabricius 2000) in Program R (version 4.4.1; R Core Team 2024). Each set of training data was used to train 20 CART models with the *rpart* (version 4.1.23; Therneau and Atkinson 2023) package using *minsplit* values between 1 and 20 and 1000 cross‐validations to obtain consistent estimates of prediction error for each tree (Breiman et al. 1984).

For each seed, we pruned each of the 20 trees using the one standard error (1‐SE) rule (Breiman et al. 1984; De'ath and Fabricius 2000) to obtain the most parsimonious model. For each tree (*n* = 20,000), we evaluated the classification accuracy of the training dataset (“unpruned tree”), the testing dataset (“unpruned tree”), the testing dataset (“pruned tree”), and the number of correctly identified presences. We also assessed the sensitivity and specificity of the pruned model, and the top three splitting variables. We selected the top model by finding the model that correctly predicted the most bridle shiner presences (highest sensitivity; Barbet‐Massin et al. 2012) while also having relatively high prediction accuracy. We then ran this model with the combined training and testing datasets to characterize its overall prediction accuracy.

### 2.3 Species Distribution Models for Maine and New Hampshire (Models 2 and 3)

#### 2.3.1 New Hampshire Bridle Shiner Surveys

The New Hampshire Fish and Game Department (NHFGD) conducted fisheries surveys between 2005 and 2022 (NHFGD 2015). Capture methods included seine netting, boat electrofishing, backpack electrofishing, dip netting, and minnow trapping across lakes, ponds, streams, and rivers (M. Carpenter, NHFGD, written communication, 07 February 2023). Surveys conducted for other fish, such as brook trout ( *Salvelinus fontinalis* ), provided incidental bridle shiner presence data, while surveys conducted specifically for bridle shiners (mostly using dip netting, seine netting, and minnow traps) noted both presence and absence.

#### 2.3.2 Presence‐Absence Data Preparation (Maine and New Hampshire)

We classified deliberate bridle shiner surveys conducted during or after 2009 as “current” surveys (Figure 2, Model 2) and incidental bridle shiner detections prior to 2009 as “historical” surveys (Figure 2, Model 3). The only exception to this was Maine's last incidental bridle shiner detection in 2010; we classified this detection as “historical” and all subsequent detections as “current.” We did not include records (*n* = 4) from sites where bridle shiners had been introduced (e.g., Marshall Brook; Doering et al. 1995) or were likely misidentified based on Maine Department of Inland Fisheries and Wildlife (MDIFW) biologist consultation. When two or more surveys were conducted at a site across years, we calculated the spatial average of the reported survey locations. We considered a site to be currently occupied if bridle shiners were found there during the most recent site survey between 2009 and 2022 (Table SD1). There were 33 survey sites with historical bridle shiner presence but no survey coordinates. In these situations, we approximated the coordinates of the sites using site descriptions (*n* = 8) or the center point of the lake (*n* = 11), pond (*n* = 11), or stream reach (*n* = 3; Table SD1).

Because scale may influence prediction outcomes, we fit SDMs at two different spatial scales. We used established HUC12s (USGS 2021) to predict bridle shiner presence at a “coarse” scale (Figure 2, Models 2a and 3a), and delineated pseudo‐HUC14s following the methods of Friedrichs‐Manthey et al. (2020) to predict presence at a “fine” scale (Figure 2, Models 2b and 3b; Supporting Information E). These pseudo‐HUC14s were approximately equivalent in size to established HUC14 polygons (USGS 2021), which were not available for the entire modeled area. For the remainder of this paper, we will refer to these newly delineated polygons as “HUC14s,” to the established HUC12 polygons as “HUC12s,” and to “drainages” when we are referring to both scales of the analysis.

**FIGURE 4:**
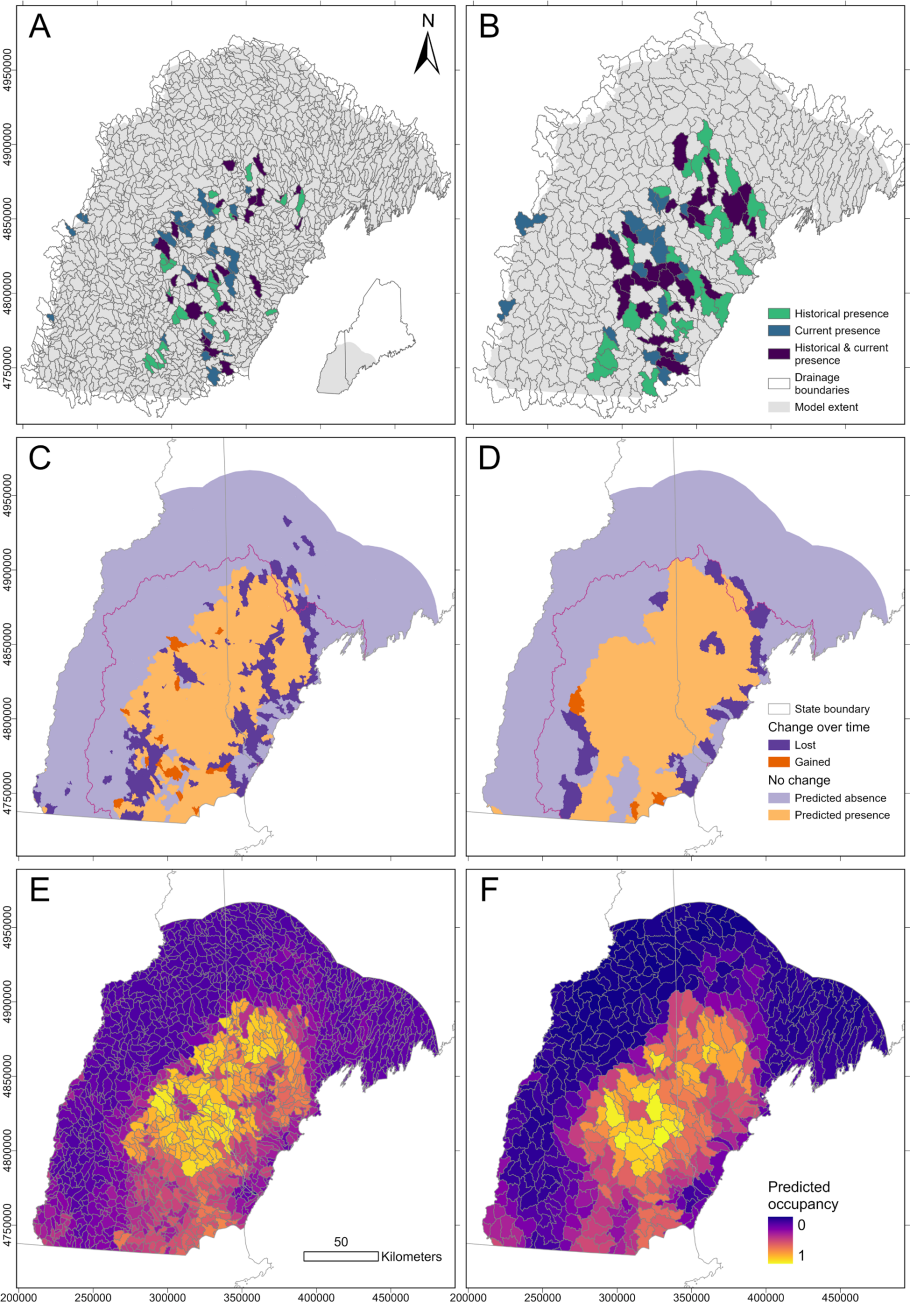
Topmost panels show occupancy status at the (A) pseudo‐Hydrologic Unit Code 14 (HUC14) and (B) HUC12 drainage scales used to model bridle shiner ( *Notropis bifrenatus* ) distribution in Maine and New Hampshire. Drainages where bridle shiners were only detected prior to 2009 (“historical”) are in green; drainages where bridle shiners were detected at least once prior to 2009 and at least once post‐2009 (“current”) are in purple; and drainages where bridle shiners were only detected after 2009 are in blue. Panels C‐F show ensemble species distribution model (SDM) predictions of bridle shiner presence over historical and current periods. Binary model results: (C) HUC14 and (D) HUC12 drainages predicted to support bridle shiners across both periods (light orange), drainages predicted to have never supported bridle shiners (light purple), drainages where SDMs predict historical presence but current absence (dark purple), and drainages where SDMs predict historical absence but current presence (dark orange). Pink lines denote the known bridle shiner range (Saco and Merrimack Hydrologic Unit Code 6 [HUC6] basins) within the two states. Continuous model results: Predicted probability of bridle shiner presence (current) at (E) HUC14 and (F) HUC12 drainage scales.

We aggregated presence points (*n* = 210) by HUC14 (Figure 4A) and HUC12 (Figure 4B) for both the current (*n* = 117) and historical (*n* = 93) time periods. We considered drainages with at least one presence point as “present” for the scale and period (e.g., HUC12 scale, historical), and all other drainages for that scale and period as having “unknown occupancy.” When we did not know the specific location of a historical survey, we took an inclusive approach and assigned presence to all HUC14s that could have contained the survey. This was only necessary around Lake Winnisquam, New Hampshire, where five additional HUC14s were included. This was not necessary at the HUC12 scale, as all potential survey locations were contained within each surveyed waterbody's corresponding HUC12. We did not include sites where bridle shiners were deliberately introduced in our SDMs (“Warren Hatchery Pond” in New Hampshire; Table SD1).

#### 2.3.3 Raster Data Preparation

We limited the spatial extent of the SDMs by buffering the known historical range of bridle shiners in New Hampshire and Maine (i.e., the Saco and Merrimack HUC6 basins) by 50‐km to account for the likely non‐detection of fish at the edges of their range (Sutton et al. 2015) while preventing unrealistic geographic assignments. We selected 35 environmental variables available in GIS repositories to include in the SDMs (Table 2). These included elevation (30‐m resolution), 27 Biophysical Settings land cover types (300‐m resolution) from the 2020 Landscape Fire and Resource Management Planning Tools program (LANDFIRE; Rollins 2009; Blankenship et al. 2021), three soil composition variables (225‐m resolution), and four lithology variables (90‐m resolution; Table 2). We used 2020 LANDFIRE Biophysical Settings land cover classifications because they are based on both the current biophysical environment and historical disturbance regimes (Rollins 2009; Blankenship et al. 2021), and so were applicable to both of our modeling time scales (1898–2008 and 2009–2022). We obtained the soil composition (clay content, sand content, and silt content of the uppermost 5‐cm of soil) rasters from the International Soil Reference and Information Centre's (ISRIC) Soil Data Hub (Poggio et al. 2021). We also included lithology classes (coarse glacial till, fine glacial lake sediment, coarse glacial outwash, and fine coastal sediment and alluvium) from the Conservation Science Partners Ecologically Relevant Geomorphology Datasets, Landforms, and Physiography dataset as these are the parent materials of soil substrates and remain stable over long time scales (Theobald et al. 2015). We calculated the mean elevation, mean soil composition, and the proportion of each LANDFIRE cover type and lithology class within each HUC14 and HUC12. We then extracted raster habitat covariates from the centroid of each HUC14 and HUC12 (*n* = 479 HUC12 points and *n* = 1747 HUC14 points).

**TABLE 2:** Rasters of regional variables used to model bridle shiner ( *Notropis bifrenatus* ) distribution in Maine and New Hampshire.

Variable name	Description	Source
elev	Elevation (m)	1
Ac_LoElev_SpruceFirHardwd	Proportion of Acadian Low‐Elevation Spruce‐Fir‐Hardwood Forest in modeling unit	2
AcAp_AlpineTundra	Proportion of Acadian‐Appalachian Alpine Tundra in modeling unit	2
AcAp_Montane_SpruceFir	Proportion of Acadian‐Appalachian Montane Spruce‐Fir Forest in modeling unit	2
AcAp_WdHeath	Proportion of Acadian‐Appalachian Subalpine Woodland and Heath‐Krummholz in modeling unit	2
Ap_Hemlock_NHardwd	Proportion of Appalachian (Hemlock‐)Northern Hardwood Forest in modeling unit	2
barren	Proportion of Barren‐Rock/Sand/Clay in modeling unit	2
Bor_Acid_PeatSys	Proportion of Boreal Acidic Peatland Systems in modeling unit	2
Bor_JackPineBlSpruce	Proportion of Boreal Jack Pine‐Black Spruce Forest in modeling unit	2
CAp_Dry_OakPine	Proportion of Central Appalachian Dry Oak‐Pine Forest in modeling unit	2
CAp_PineOak_Rocky_Wd	Proportion of Central Appalachian Pine‐Oak Rocky Woodland in modeling unit	2
CIntAp_FldplnSys	Proportion of Central Interior and Appalachian Floodplain Systems in modeling unit	2
CIntAp_RiparSys	Proportion of Central Interior and Appalachian Riparian Systems in modeling unit	2
CIntAp_SwampSys	Proportion of Central Interior and Appalachian Swamp Systems in modeling unit	2
GulfAtl_CstPln_SwampSys	Proportion of Gulf and Atlantic Coastal Plain Floodplain Systems in modeling unit	2
GulfAtl_CstPln_TMarshSys	Proportion of Gulf and Atlantic Coastal Plain Tidal Marsh Systems in modeling unit	2
LAc_FldplnSys	Proportion of Laurentian‐Acadian Floodplain Systems in modeling unit	2
LAc_NHardwd	Proportion of Laurentian‐Acadian Northern Hardwoods Forest in modeling unit	2
LAc_NPineOak	Proportion of Laurentian‐Acadian Northern Pine(‐Oak) Forest in modeling unit	2
LAc_PineHemlockHardwd	Proportion of Laurentian‐Acadian Pine‐Hemlock‐Hardwood Forest in modeling unit	2
LAc_ShrubHerb_WetlSys	Proportion of Laurentian‐Acadian Shrub‐Herbaceous Wetland Systems in modeling unit	2
LAc_SwampSys	Proportion of Laurentian‐Acadian Swamp Systems in modeling unit	2
NCInt_Wet_Flatwd	Proportion of North‐Central Interior Wet Flatwoods in modeling unit	2
NeInt_PineBarrens	Proportion of Northeastern Interior Pine Barrens in modeling unit	2
NAtl_CstPln_Dun	Proportion of Northern Atlantic Coastal Plain Dune and Swale in modeling unit	2
NAtl_CstPlain_Hardwd	Proportion of Northern Atlantic Coastal Plain Hardwood Forest in modeling unit	2
NAtl_CstPln_Mar	Proportion of Northern Atlantic Coastal Plain Maritime Forest in modeling unit	2
water	Proportion of Open Water in modeling unit	2
clay	Clay content of soil (g/kg) at 0‐5 cm	3
sand	Sand content of soil (g/kg) at 0‐5 cm	3
silt	Silt content of soil (g/kg) at 0‐5 cm	3
Glac_till_crs	Proportion of Glacial Till Coarse in modeling unit	4
Glac_lk_sed_fin	Proportion of Glacial Lake Sediment Fine in modeling unit	4
Glac_out_crs	Proportion of Glacial Outwash Coarse in modeling unit	4
Alluv_cst_sed_fine	Proportion of Alluvium and Coastal Sediment Fine in modeling unit	4

*Source:* (1) Digital Elevation Model (USGS 1998); (2) LANDFIRE Biophysical Settings (USFS and USGS 2022); (3) SoilGrids (Poggio et al. 2021); (4) Lithology dataset (Theobald et al. 2015).

#### 2.3.4 BIOMOD Models

We used the classification tree analysis (CTA; Breiman et al. 1984), generalized boosted models (GBM; Ridgeway 1999; González‐Ferreras et al. 2016), and random forest (RF; Breiman 2001) modeling options in R package *biomod2* (version 4.2–5; Thuiller et al. 2009) to explore the relationships between the 35 habitat covariates and the current and historical bridle shiner distributions in Maine and New Hampshire. All three of these models employ machine‐learning classification methods that can fit complex nonlinear relationships, and thus do not require the transformation of non‐normally distributed data or the removal of highly correlated covariates (Breiman et al. 1984; Breiman 2001; Muñoz and Felicísimo 2004; González‐Ferreras et al. 2016).

We did not use generalized linear models (GLM), generalized additive models (GAM), or Maxent because these models require a larger number of pseudo‐absence points than classification and machine‐learning models (Barbet‐Massin et al. 2012). Global parametric models such as GLMs are also sensitive to multicollinearity and require simplifications that may result in models that perform poorly on ecological data (Muñoz and Felicísimo 2004). We chose an ensemble model approach to emphasize the trends emerging from the data while reducing the noise from individual model outputs (Araújo and New 2007).

Following the process outlined in Hao et al. (2019), we used a two‐step internal validation approach to divide our data into “outer” training and testing (“evaluation”) datasets, and then to further divide the training data into “inner” training (“calibration”) and testing (“validation”) datasets. This approach ensured that a subset of the data (the evaluation dataset) was held apart to assess and compare the intermediate performance‐informed ensemble models (Marmion et al. 2009; Meller et al. 2014; Hao et al. 2019) and to provide a direct measure of model transferability (Fielding and Bell 1997; Wenger and Olden 2012). Detailed ensemble modeling methods can be found in Supporting Information F.

For each of the final ensemble models, we then joined the drainage polygons to their centroid points to create shapefiles of both continuous probability of presence and binary presence‐absence. Binarizing the model outputs allowed us to quantify the predicted area of suitable habitat (Fourcade 2021). We then calculated the proportion of predicted historically occupied drainages (Models 2a and 2b) that were also predicted to currently support bridle shiners (Models 3a and 3b) at both spatial scales to determine the overall change in predicted occupied drainages (Figure 2).

#### 2.3.5 Variable Importance

Finally, we fit exploratory GLMs to assess the relationship of each SDM predictor variable to the final four ensemble model predictions. Although GLMs may mask interactions among predictor variables and are sensitive to multicollinearity (Muñoz and Felicísimo 2004), we used them in a strictly exploratory and inferential sense to gauge the relative importance of the predictor variables to the final ensemble models. We evaluated each predictor variable with a separate GLM and determined effect size using covariate *z*‐scores.

## 3 Results

### 3.1 Local Habitat Variables (Model 1)

We fit 20,000 CART models using 1000 combinations of training and testing data and *minsplit* values between 1 and 20. Of these, 11,243 models produced viable classification trees (trees with an estimated error rate within one standard error of the minimum error; De'ath and Fabricius 2000). We used model sensitivity and overall model accuracy to select the top four CART models (Models 1a–1d; Table SC1). The two models with the highest sensitivity (71.4%) were Models 1a and 1b: Model 1a identified a proportion of SAV (“subm”) greater than 65% as the top splitting variable and Model 1b identified dominant substrate (“dom.substrate” not equal to detritus, gravel, pea gravel, sand, or silt) as the top splitting variable. Models 1c and 1d had the highest overall classification accuracy (86.8%): Model 1c identified a proportion of SAV greater than 65% as the top splitting variable and Model 1d identified the absence of watermilfoils (*Myriophyllum* spp. other than *M. tenellum* ) as the top splitting variable.

We then combined the training and testing datasets and evaluated the performance of the four models at all 95 sites (Table SC1). The model with the highest sensitivity (Model 1a; Figure SC1) correctly classified 69 sites (accuracy = 72.6%), correctly predicted 13 presences (sensitivity = 76.5%), and correctly predicted 56 absences (specificity = 71.8%). The model with the highest accuracy (Model 1c; Figure SC2) correctly classified 87 sites (accuracy = 91.2%), correctly predicted 10 presences (sensitivity = 58.8%), and correctly predicted 77 absences (specificity = 98.7%; Table SC1).

There were several sites that were misclassified by two or more of the top models. None of the top models correctly predicted presence at GWORKS, and only one of the top models correctly predicted presence at sites CRESLK‐01, OSSIPE, and OSSIPM. Only half of the top models accurately classified the following 10 sites: BRADPD‐02, CRESLK‐02, DUCKIN, HEATH‐02, HIGHLK‐04, JORDAN, MOSQPD, MUDNO, OCSACO, and SEBAGO‐04 (Table SC2).

### 3.2 Species Distribution Models (Models 2 and 3)

We modeled the historical and current range of the bridle shiner within Maine and New Hampshire at both the HUC12 (coarse) and HUC14 (fine) scales. Of the three individual model types, GBM and RF models had the highest cross‐validated receiver operating characteristic curve metric (ROC; Fielding and Bell 1997) across all individual model runs (Table SF1). True skill statistic (TSS; Allouche et al. 2006) values for these models, however, indicated poor model performance (Table SF1). CTA models performed fairly well when evaluated by both ROC and TSS. Models run with historical presence data performed slightly better than those run with current data at both spatial scales (Table SF1). This pattern was no longer evident when fitting the intermediate ensemble models: The mean AUC and mean TSS were similar between periods at both spatial scales (Table SF2). There were fewer ensemble models fit at the HUC12 scale than at the HUC14 scale, meaning that more individual models at the HUC12 scale had TSS values below 0.5 (Table SF2). All final ensemble model types performed better than random (AUC > 0.70; TSS > 0.50) for both the historical and current periods (Table SF2). The historical HUC12 (Model 2a) model had a TSS score slightly below 0.60 and an AUC slightly below 0.8, indicating only moderate support for this model (Landis and Koch 1977).

Models at both fine and coarse spatial scales predicted an overall loss of between 14% (HUC12 scale) and 24% (HUC14 scale) of historically occupied drainages along the periphery and interior of the bridle shiner range (Figure 4C,D; Table SF3). Predicted range loss was more pronounced in Maine, where ensemble models predicted a net decrease in occupied drainages between 21% (HUC12 scale) and 36% (HUC14 scale). Ensemble models also predicted current occupancy at some drainages where bridle shiners were predicted to be absent prior to 2009.

#### 3.2.1 Variable Importance

**FIGURE 5:**
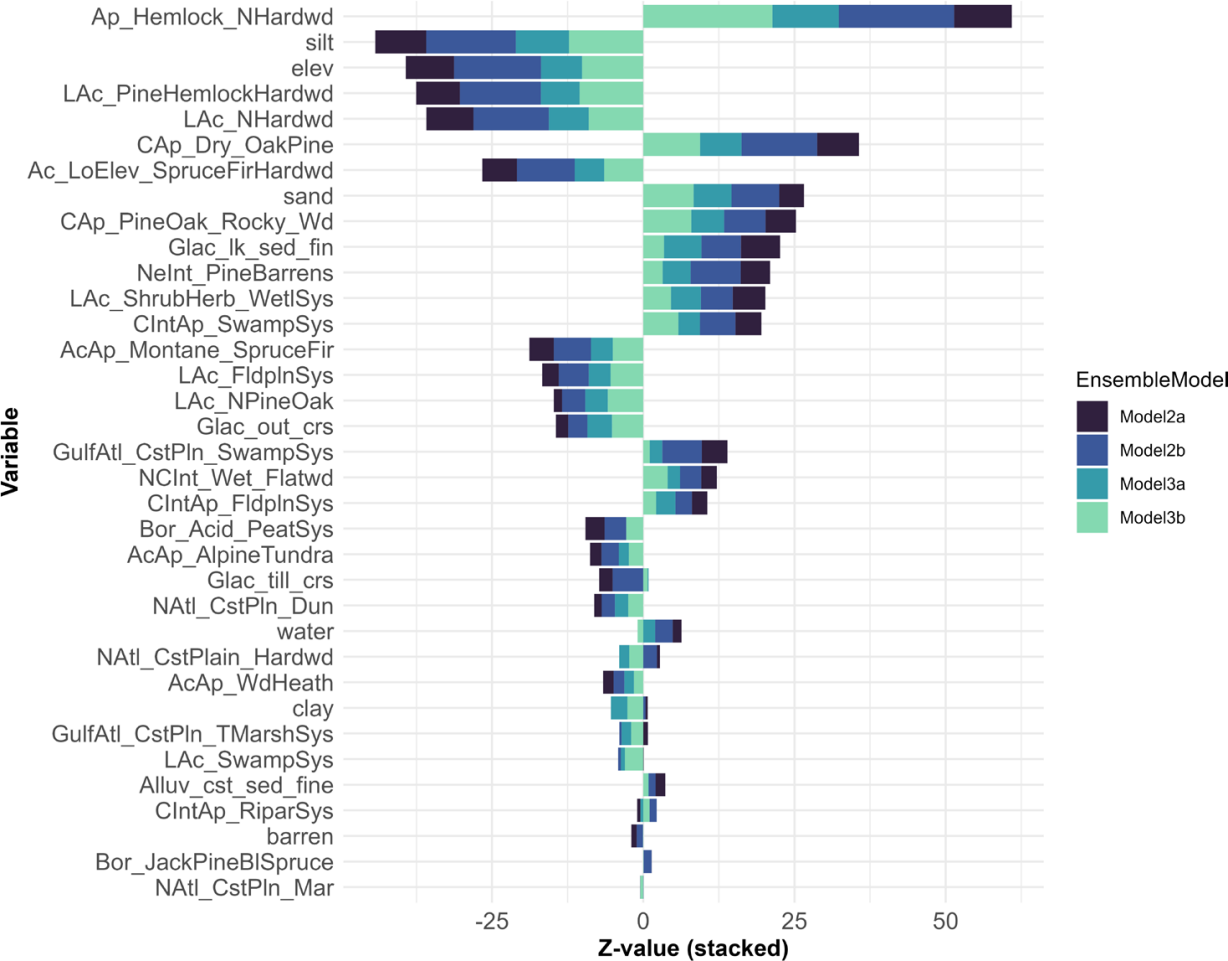
Relationship between ensemble species distribution model (SDM) predictions of binary bridle shiner ( *Notropis bifrenatus* ) presence‐absence and the proportion of 35 topographic, lithologic, and land cover variables in a drainage. Four final models were compared: One fit to historical (1898–2008) presence data across coarse‐scale Hydrologic Unit Code 12 (HUC12) polygons (Model 2a), one fit to historical presence data across fine‐scale HUC14s (Model 2b), one fit to current (2009–2022) presence data across HUC12s (Model 3a), and one fit to current presence data across HUC14s (Model 3b). The importance of each explanatory variable to each of the final models was assessed using generalized linear model (GLM) *z*‐scores. Variable name abbreviations are outlined in Table 2.

We fit exploratory generalized linear models to assess the relationship of each SDM predictor variable to the final four ensemble model predictions. Predicted bridle shiner presence was associated with up to 26 habitat variables (*p* < 0.05), and these associations differed over period and spatial scale (Figure 5). The most influential variable across all models was Appalachian (Hemlock‐)Northern Hardwood Forest (“Ap_Hemlock_NHardwd”), followed by silt, elevation (“elev”), Laurentian‐Acadian Pine‐Hemlock‐Hardwood Forest (“LAc_PineHemlockHardwd”), and Laurentian‐Acadian Northern Hardwoods Forest (“LAc_NHardwd”; Figure 5).

Predicted presence was strongly positively associated with 10 variables in all four models: Appalachian (Hemlock‐)Northern Hardwood Forest, Central Appalachian Dry Oak‐Pine Forest (“Cap_Dry_OakPine”), Northeastern Interior Pine Barrens (“NeInt_PineBarrens”), sand, Central Appalachian Pine‐Oak Rocky Woodland (“Cap_PineOak_Rocky_Wd”), fine glacial lake sediment (“Glac_lk_sed_fin”), Central Interior and Appalachian Swamp Systems (“CIntAp_SwampSys”), Laurentian‐Acadian Shrub‐Herbaceous Wetland Systems (“LAc_ShrubHerb_WetlSys”), North‐Central Interior Wet Flatwoods (“NCInt_Wet_Flatwd”), and Central Interior and Appalachian Floodplain Systems (“CIntAp_Fldpln_Sys”; Figure 5).

Predicted presence was strongly negatively associated with eight variables in all four models: Silt, elevation, Laurentian‐Acadian Pine‐Hemlock‐Hardwood Forest, Laurentian‐Acadian Northern Hardwoods Forest, Acadian Low‐Elevation Spruce‐Fir‐Hardwood Forest (“Ac_LoElev_SpruceFirHardwd”), Acadian‐Appalachian Montane Spruce‐Fir Forest (“AcAp_Montane_SpruceFir”), Laurentian‐Acadian Floodplain Systems (“LAc_FldplnSys”), and coarse glacial outwash (“Glac_out_crs”; Figure 5). Variable importance to the final ensemble models differed by spatial scale. The relationship between predictor variables and predicted occupancy was stronger (larger absolute *z*‐values) in fine‐scale models fit to HUC14s than in coarse‐scale models fit to HUC12s. A higher proportion of clay soils in a drainage was not a significant predictor of occupancy at a fine scale but was significantly negatively associated with bridle shiner presence at the HUC12 scale.

Variable importance of the final ensemble models also differed over time. Historical models suggested a positive association between Northern Atlantic Coastal Plain Hardwood Forest land cover and bridle shiner presence, but current models showed the opposite (Figure 5). Acadian Low‐Elevation Spruce‐Fir‐Hardwood Forest, fine glacial lake sediment, Northeastern Interior Pine Barrens, Boreal Acidic Peatland Systems (“Bor_Acid_PeatSys”), and Gulf and Atlantic Coastal Plain Swamp Systems (“GulfAtl_CstPln_SwampSys”) were more closely associated with bridle shiner occupancy in historical models than in current models.

## 4 Discussion

### 4.1 Local Habitat Predictions for Bridle Shiners

Bridle shiners inhabit shallow, vegetated areas in ponds, lakes, and rivers (Jenkins and Burkhead 1994; Page and Burr 2011), but sources differ in their identification of which vegetation and substrate types make up the species' habitat. Some sources specify that only submerged aquatic vegetation (e.g., Geneva et al. 2018; Pregler et al. 2019), or that both submerged and emergent vegetation are preferred (Jensen and Vokoun 2013). Others report that submerged, emergent, or floating vegetation is suitable habitat (Holm et al. 2001). Page and Burr (2011) report that bridle shiners inhabit sluggish, mud‐bottomed pools, while bridle shiners in Connecticut select reaches or habitat patches with unconsolidated bottoms and silty substrate (Jensen and Vokoun 2013; Pregler et al. 2019).

We assessed associations between aquatic plants, substrate, and bridle shiner occurrence in Maine (2021–2022). Most sites surveyed in Maine were dominated by organic matter (mud or detritus) or sand (e.g., Figure SB1). Of the eight sites dominated by silt substrate, none supported bridle shiners. The most accurate CART model (Model 1c) suggested that when the proportion of SAV at a site is below 65% and watermilfoils are present, bridle shiners are found at sites with less than 75% organic substrates (determined via visual surveys). This series of splitting variables was based on three sites (COLCPD‐01, PRESUM‐01, and SEBAGO‐01) with substrates composed of 50%–60% organic material and 40%–45% sand or silt inorganic materials. This lack of association with silt substrate may reflect different habitat selection behavior by bridle shiners at the periphery of their range but may also simply be due to differences in substrate availability between Maine and Connecticut. The local habitat model results may also reflect that sites in Maine were often dominated by patches of SAV and organic substrate interspersed with patches with little SAV cover dominated by sand or silt substrate. We did not measure the size of the SAV patches or the degree of interspersion between habitat types, but these site characteristics may have influenced bridle shiner presence at sites with less overall SAV cover.

We found that Maine bridle shiners were associated with sites that have a higher proportion of SAV than emergent vegetation, floating vegetation, or open water. According to the two top CART models (Models 1a and 1c), sites with at least one species of complex‐leaved watermilfoil were also more likely to support bridle shiners (Figures SC1 and SC2). This corroborates the observations of Harrington (1947a), where bridle shiners in New Hampshire spawned over stands of native watermilfoil. We did not distinguish between native and invasive species of watermilfoil in our analysis because of their structural similarities and the difficulty of distinguishing invasive two‐leaf watermilfoil from native species such as whorl‐leaf watermilfoil ( *M. verticillatum* ) or alternate‐flowered watermilfoil ( *M. alterniflorum* ). We did not identify any Eurasian watermilfoil ( *M. spicatum* ) at our sites, and as of 2008 this species had only been detected in one waterbody in Maine (Bailey and Calhoun 2008).

We also found bridle shiners using other species of SAV as habitat (e.g., bladderworts [Figure SB2], swaying bulrush [Figure SB3], and watercelery). These species were common to the survey region and to multiple sites where bridle shiners were absent, so they could not be regarded as splitting variables in the CART models. While other studies have demonstrated an association between bridle shiners and coontail ( *Ceratophyllum demersum* ; Hanson 2013; Starnes et al. 2025), this species did not form dense stands at the majority of our sites.

While invasive watermilfoil species may appear physically similar to native macrophytes such as hornworts (*Ceratophyllum* spp.), bladderworts, and native watermilfoils, it is unknown how the spread of these plants will affect bridle shiners. Bridle shiners spawn at sites that permit unobstructed movement above SAV (Harrington 1947a). While native watermilfoils usually grow in scattered clumps, invasive watermilfoils outcompete native macrophytes, reduce water flow, and form a significant barrier to fish movement and foraging (Keast 1984). The reduced flow and finely divided, feather‐like watermilfoil leaves trap substrates and decomposing organic matter, which lowers dissolved oxygen levels (Sculthorpe 1967; Keast 1984; Mathai et al. 2018). Unchecked spread at a site could reduce habitat suitability for bridle shiners as the invasive watermilfoil fills in the gaps above and between stands of SAV that are required for movement and spawning (Harrington 1947a; Keast 1984). Two historically occupied sites in Maine, for example (JORDAN and JOSIES), are now packed with dense stands of two‐leaf watermilfoil (this study). While we do not know why bridle shiners no longer occupy these sites, these habitats are now most likely too degraded to support them.

### 4.2 Regional Species Distribution Models for Historical and Current Distributions

The recent bridle shiner surveys in Maine and New Hampshire allowed us to model both their historical (1898–2008) and current (2009–2022) distribution in the region. We found that bridle shiner presence at both coarse (HUC12) and fine (HUC14) scales in this region was influenced by dominant land cover type, elevation, soil composition, and lithology. The four final ensemble models had high model performance as determined by AUC and TSS statistics.

We included the proportions of a suite of substrate composition and land cover types in our models and found that Appalachian (Hemlock‐)Northern Hardwood Forest cover was strongly associated with bridle shiner presence. The influence of this forest type may be specific to this region, as it does not extend much further south than New Hampshire. High proportions of other forest types, such as Central Appalachian Dry Oak‐Pine Forest or Northern Atlantic Coastal Plain Hardwood Forest, may be more predictive of bridle shiner occurrence in central and southern portions of their range. Predicted bridle shiner presence was strongly negatively associated with Laurentian‐Acadian forest types, which are cooler and drier than Appalachian forests (Anderson et al. 2013). Appalachian (Hemlock‐)Northern Hardwood Forest grades into Laurentian‐Acadian Pine‐Hemlock‐Hardwood forest in western New Hampshire and northeastern Maine, and the component vegetation associations change with latitude (Anderson et al. 2013).

The bridle shiner range may therefore have been historically limited by mountains to the north and west of the Merrimack basin and by colder, drier Laurentian‐Acadian habitats to the north and east of the Saco basin. We also found that drainages with higher soil sand content (via ISRIC soil composition rasters; Poggio et al. 2021) were more likely to support bridle shiners, while drainages with soil high in silt or clay were less likely to support bridle shiners. This is especially apparent in Maine, where soils high in sand are replaced by soils higher in clay and silt to the east of Sebago Lake, and most of this clay‐silt region is not predicted to support bridle shiners. Our ensemble models predicted that much of the once‐suitable habitat in the central Saco and southwestern Merrimack has been lost, and the limits of the bridle shiner range seem to be shifting away from coastal areas (Figure 4C–F). Bridle shiners were predicted to have historically occupied drainages east of the Saco basin (Lower Androscoggin HUC8), but only a small portion of this area remains in the current model (Figure 4C).

Our models of historical bridle shiner presence were most likely underestimating the full extent of the bridle shiner's range because they were based entirely on incidental observations, while current models were based on targeted surveys. False absences are likely to be common as surveys have used a variety of equipment and protocols to detect bridle shiners, and detection probability at the waterbody scale (e.g., entire lakes and ponds) can be low (Katz et al. 2024). Comparisons of range extent between time periods are therefore imprecise, and, because of biases in gear, sampled habitat, and survey goals (Stone et al. 2001; Katz et al. 2024), models of the historical bridle shiner range in the region are likely an underestimate. This is one possible explanation for why some drainages were predicted to have current but not historical bridle shiner presence (Figure 4C,D), as range expansion is unlikely due to extensive habitat fragmentation (Fagan 2002).

Surveys at historical sites in both New Hampshire and Maine suggest that bridle shiner declines in the region may be more pronounced than suggested by our SDMs. Ensemble models predicted that 14%–24% of historically suitable habitat has been lost in this region (depending on the spatial scale), with losses in Maine being the most pronounced (20%–36% loss). However, survey data suggest that losses have been more pronounced in New Hampshire: NHFGD surveys have determined that bridle shiners remain at only 8 of 30 historical sites (73% loss) occupied in 1947 (Harrington 1947b; M. Carpenter, NHFGD, written communication, 27 June 2023). Multiple surveys at these locations have confirmed that significant habitat degradation rendered the sites unsuitable for bridle shiners. Similarly, bridle shiner populations remain at only 11 of 30 historically occupied sites (63% loss) or waterbodies in Maine (excluding sites with likely false positive historical records; Katz et al. 2024). These surveys suggest that, although bridle shiners may have historically been more widely distributed in New Hampshire and Maine than previously thought, our current ensemble models are likely underestimating the true scale of bridle shiner declines in these states.

One of the limitations of using pseudo‐absence points in SDMs is the high degree of class overlap between presence and background variables: A portion of the randomly generated points will occur in areas that have suitable habitat and may even have undocumented populations of the species (i.e., introducing false negatives into the model; Valavi et al. 2021). Generating many pseudo‐absence points is necessary when characterizing the range of environmental conditions in the modeled area, which creates an imbalance between the number of presence and background points (Valavi et al. 2021). In general, presence‐absence data are preferred to presence‐only data because observed zeros are more informative than points with unknown occupancy (Royle et al. 2012). Because we aggregated bridle shiner surveys at the HUC14 or HUC12 scale, we were only able to use presence points (i.e., absence at a site could not be interpreted as absence within an entire drainage). This reduced spatial autocorrelation (Phillips et al. 2017) and the ambiguity involved in delineating one survey site from another (e.g., the many survey sites in neighboring coves at Lake Winnipesaukee). Aggregating presences also allowed us to include entire lakes and ponds as presences when we did not have precise historical survey coordinates, which reduced the inclusion of false absences. The disadvantages of using drainages as our modeling unit were that we did not have the ability to predict occupancy at individual waterbodies within a drainage, and we could not compare historical surveys with imprecise location data to recent surveys in the area. It was also not feasible for us to survey entire lakes and larger ponds in Maine. Future bridle shiner surveys may consider increasing sampling effort in line with waterbody size and sampling sites more than once to increase detection probability (Katz et al. 2024).

There are several environmental variables that are currently unavailable in spatial databases that could potentially improve future iterations of our SDMs. Combining bathymetry data with other features such as river and lake substrate, water velocity, SAV density, salinity (Cooper 1985), and pH could provide detailed predictions of specific areas within waterbodies where bridle shiners are likely to persist. Measures of general land cover composition, such as the 2019 National Land Cover Dataset (NLCD) mixed, coniferous, and deciduous forest classifications or the LANDFIRE Existing Vegetation Type layers may be more appropriate for modeling larger portions of the bridle shiner's current range because they include anthropogenic disturbances such as developed land, roads, and farmland. Finally, we did not include bioclimatic variables in our SDMs because we were modeling over a small region; future bridle shiner SDMs over the entire bridle shiner range may benefit from including these variables.

### 4.3 Implications and Considerations for Conservation

Bridle shiner declines are likely due to the same factors that affect other minnow species, especially habitat loss and degradation. Bridle shiners are vulnerable to practices such as lake drawdowns and herbicide use because they live on the shoreline and require access to abundant vegetation (Pregler et al. 2019). Several populations in New Hampshire have been extirpated due to shoreline habitat loss, lake drawdowns, eutrophication, and herbicide use (NHFGD 2015; M. Carpenter, NHFGD, written communication, 27 June 2023). Occupancy modeling has shown that bridle shiners can be reliably detected via seine net (Jensen and Vokoun 2013; Pregler et al. 2015) and eDNA (Katz et al. 2024) at the site scale, so range‐wide declines likely reflect true absences and extirpations rather than a failure to detect the species.

A problem for management of invasive watermilfoils is that they can sometimes form habitat for rare and vulnerable native species such as the bridle shiner (Gross et al. 2020). Bridle shiners have been observed living and spawning in stands of invasive two‐leaf watermilfoil in Maine (this study) and New Hampshire (NHFGD 2022). Large‐scale herbicide treatments for two‐leaf watermilfoil have greatly reduced or extirpated bridle shiner populations at multiple lakes and ponds in New Hampshire (NHFGD 2015; M. Carpenter, NHFGD, written communication, 27 June 2023).

Bailey and Calhoun (2008) found that hand removal and benthic mats are effective two‐leaf watermilfoil management techniques in Maine. Hand removal could be used as an alternative to herbicide use at sites with bridle shiner presence where invasive watermilfoil is either interspersed with native aquatic plants or where it forms small, high‐density stands (Bailey and Calhoun 2008). Benthic mats could be used in larger areas with dense stands of two‐leaf watermilfoil. While these barriers also prevent the growth of native species of SAV, these species recolonize the area after the mat is removed (Bailey and Calhoun 2008).

NHFGD (2015) has also determined that sudden, artificial water level fluctuations at dams threaten bridle shiner populations both upstream and downstream of the dam. While we did not find evidence that the number of dams within 2‐km of a site influences bridle shiner presence in Maine (Model 1), bridle shiners are known to utilize dam headponds (Geneva et al. 2018; Pregler et al. 2019; Starnes et al. 2025). Eight of Maine's 17 occupied sites are in artificially impounded lakes or ponds or near a dam on a stream or river. Although dam impoundments can provide habitat for cyprinids, these impoundments also support a higher relative abundance of large piscivores (Whittum et al. 2023; Starnes et al. 2025). In several instances, bridle shiners have become extirpated from such habitats after sudden water level drops or dam breaches (NHFGD 2015). It is possible that these artificial habitats are population sinks for bridle shiners because of the high risk of predation and sudden water level fluctuations. Annual wintertime drawdown, for example, is a common management strategy used for hydroelectric power generation, flood control, and invasive aquatic plant control (Mjelde et al. 2013; Carmignani and Roy 2017, 2021; He et al. 2023). Drawdowns reduce the abundance of aquatic plants in littoral zones both by directly exposing them to desiccation and by reducing silt and organic matter cover within exposure zones (Carmignani and Roy 2021). These drawdowns may have long‐term impacts on cyprinid populations by reducing the SAV habitat available for reproduction (Yamamoto et al. 2006; Pregler et al. 2019) and increasing vulnerability to predation (Sutela et al. 2011; Starnes et al. 2025). Working with dam managers, hydroelectric companies, or state agencies may be necessary to mitigate these effects and prevent local extirpations (M. Carpenter, NHFGD, written communication, 27 June 2023). When dams are removed to recover ecosystem function, managers may consider translocating vulnerable bridle shiner populations and then reintroducing them when sustainable SAV habitat is restored (Pires et al. 2021).

## 5 Conclusions

Locating additional populations of bridle shiner in Maine and New Hampshire, especially at the periphery of their predicted range in Maine, may be critical to preventing further declines. These peripheral populations may merit high conservation priority because they are at the leading edge of the species' potential northward expansion in response to climate change (Gibson et al. 2009). We found that bridle shiners were associated with sites with abundant SAV, a high level of organic substrate, and the presence of at least one complex‐leaved watermilfoil species.

Ensemble SDMs at the HUC12 and HUC14 scale predicted that bridle shiner presence was associated with Appalachian (Hemlock‐)Northern Hardwood Forest, sand substrate, and low‐elevation terrain, and negatively associated with Laurentian‐Acadian forest types within a drainage. Our local and regional models may be used to focus surveys on areas across Maine and New Hampshire with high predicted habitat suitability. In addition to guiding the search for undiscovered populations of bridle shiner, managers may also use these models to search for suitable reintroduction sites or focus habitat restoration efforts.

## Author Contributions

**Lara S. Katz:** data curation (equal), formal analysis (lead), funding acquisition (supporting), investigation (equal), methodology (equal), software (lead), validation (supporting), visualization (lead), writing – original draft (lead), writing – review and editing (lead). **Stephen M. Coghlan Jr.:** conceptualization (equal), funding acquisition (lead), methodology (equal), supervision (supporting), validation (equal), writing – review and editing (supporting). **Matthew A. Carpenter:** data curation (equal), investigation (equal), methodology (equal), resources (equal), validation (equal), writing – review and editing (supporting). **Michael T. Kinnison:** methodology (equal), resources (equal), validation (equal), writing – review and editing (supporting). **Joseph D. Zydlewski:** conceptualization (equal), funding acquisition (lead), methodology (equal), project administration (lead), supervision (lead), validation (equal), visualization (supporting), writing – review and editing (lead).

## Ethics Statement

Field surveys in Maine were conducted under a MDIFW Scientific Fish Collectors Permit and Acadia National Park Scientific Research and Collecting Permit No. ACAD‐2021SCI‐0067. Fish handling followed University of Maine IACUC guidelines under permit A2021‐03‐01. Field surveys in New Hampshire were conducted by NHFGD.

## Conflicts of Interest

The authors declare no conflicts of interest.

## Supporting information


**Appendix S1:** ece372413‐sup‐0001‐AppendixS1.docx.

## Data Availability

Data generated or analyzed during this study are provided in full within the published article, its Supporting Information, and its Dryad Digital Repository (Katz et al. 2025). Questions about the data and code may be directed to Lara Katz (lara.katz@maine.edu).
