# Supplementary material for: Habitat‐Based Predictions of Bridle Shiner (Notropis bifrenatus) in the Northeastern United States
Source: Ecol Evol. 2026 Jan 12;16(1):e72413. doi: 10.1002/ece3.72413 (PMC12796511; doi:10.1002/ece3.72413)
Supplement: Supplementary file 1 — Appendix S1: ece372413‐sup‐0001‐AppendixS1.docx. [file ECE3-16-e72413-s001.docx]

**Title:** Habitat-based predictions of bridle shiner (*Notropis bifrenatus*) in the northeastern United States; Supporting Information

**Authors**: Lara S. Katz^1^, Stephen M. Coghlan, Jr. ^1^, Matthew A. Carpenter^2^, Michael T. Kinnison^3^, and Joseph D. Zydlewski^4,1^

**Author affiliations**: ^1^Department of Wildlife, Fisheries, and Conservation Biology, University of Maine, 5755 Nutting Hall, Orono, ME 04469.

^2^New Hampshire Fish and Game Department, 11 Hazen Drive, Concord, NH 03301.

^3^Maine Center for Genetics in the Environment, Murray Hall, 23 Flagstaff Rd, Orono, ME 04469.

^4^U.S. Geological Survey, Maine Cooperative Fish and Wildlife Research Unit, University of Maine, 5755 Nutting Hall, Orono, ME 04469.

**Corresponding author:**

Lara S. Katz

5755 Nutting Hall, Orono, ME 04469

Email: lara.katz@maine.edu

Phone: (207) 631-6223

*Any use of trade, firm, or product names is for descriptive purposes only and does not imply endorsement by the U.S. Government.*

**Supplement A. Maine bridle shiner presence-absence surveys.**

**Table SA1**. Maine bridle shiner (*Notropis bifrenatus*) 2021-2022 presence-absence and local habitat survey sites (NAD83/UTM Zone 19N; from Katz et al. 2024).

| Site | Waterbody name | Year(s) sampled | Easting | Northing | Survey method | Present/Absent |
| --- | --- | --- | --- | --- | --- | --- |
| ANDROS | Androscoggin River | 2022 | 405085 | 4877020 | eDNA | Absent |
| BARKER | Barker Pond | 2021, 2022 | 359052 | 4860990 | eDNA + seine | Present |
| BEARPD-01 | Bear Pond | 2021 | 362597 | 4890820 | eDNA + seine | Absent |
| BEARPD-02 | Bear Pond | 2021 | 363317 | 4889350 | eDNA + seine | Absent |
| BOOMBR | Unnamed brook | 2021 | 377144 | 4819870 | eDNA + seine | Absent |
| BRADPD-01 | Bradley Pond | 2022 | 351426 | 4899890 | eDNA | Absent |
| BRADPD-02 | Bradley Pond | 2022 | 351071 | 4899370 | eDNA | Absent |
| BROBRK | Brown's Brook | 2021 | 554524 | 4967970 | eDNA + seine | Absent |
| BUCKBR | Buck Meadow Brook | 2022 | 350874 | 4869460 | eDNA | Present |
| BUFFBR | Buff Brook | 2022 | 356590 | 4828550 | eDNA | Absent |
| BURNPD-01 | Burnt Meadow Pond | 2021 | 348493 | 4865680 | eDNA + seine | Absent |
| BURNPD-02 | Burnt Meadow Pond | 2021 | 348628 | 4865060 | eDNA + seine | Absent |
| CARBRK | Carsley Brook | 2022 | 368265 | 4882340 | eDNA | Absent |
| CHANBR | Chandler Brook | 2022 | 401963 | 4862410 | eDNA | Absent |
| COLCPD-01 | Colcord Pond | 2022 | 342804 | 4855520 | eDNA | Present |
| COLCPD-02 | Colcord Pond | 2022 | 342639 | 4857680 | eDNA | Absent |
| CRESLK-01 | Tenny River | 2022 | 382630 | 4867340 | eDNA | Present |
| CRESLK-02 | Crescent Lake | 2022 | 383820 | 4869380 | eDNA | Present |
| CRESLK-03 | Crescent Lake | 2022 | 382678 | 4871880 | eDNA | Absent |
| CROOKN | Crooked River | 2022 | 357338 | 4900570 | eDNA | Absent |
| CROOKR | Crooked River | 2021 | 373952 | 4873110 | eDNA + seine | Absent |
| CROOKS | Crooked River | 2022 | 374475 | 4870820 | eDNA | Absent |
| DINGLY | Dingley Brook | 2022 | 378789 | 4863140 | eDNA | Absent |
| DUCKIN | Duck Pond Brook | 2022 | 358274 | 4884980 | eDNA | Absent |
| DUCKNO | Duck Pond Brook | 2022 | 357422 | 4889870 | eDNA | Absent |
| EDDYBR | Eddy Brook | 2022 | 393679 | 4868000 | eDNA | Absent |
| GRTBRK | Great Brook | 2022 | 346438 | 4846970 | eDNA | Absent |
| GWORKB | Bauneg Beg Pond | 2021 | 359014 | 4801930 | eDNA + seine | Absent |
| GWORKN | Great Works River | 2021 | 357524 | 4805680 | eDNA + seine | Absent |
| GWORKS | Great Works River | 2021 | 358935 | 4797490 | eDNA + seine | Present |
| HALEY | Unnamed brook | 2022 | 353782 | 4844520 | eDNA | Unknown* |
| HEATH-01 | The Heath | 2022 | 382068 | 4875170 | eDNA | Absent |
| HEATH-02 | The Heath | 2022 | 381920 | 4874680 | eDNA | Absent |
| HIGHLK-02 | Highland Lake | 2021 | 358466 | 4884480 | eDNA + seine | Absent |
| HIGHLK-03 | Highland Lake | 2021 | 362817 | 4879480 | eDNA + seine | Absent |
| HIGHLK-04 | Highland Lake | 2021 | 359879 | 4884760 | eDNA + seine | Present |
| INGALS-01 | Ingalls Pond | 2022 | 355979 | 4858140 | eDNA | Absent |
| INGALS-02 | Ingalls Pond | 2022 | 356014 | 4857860 | eDNA | Absent |
| JORDAN | Panther Run | 2021 | 382444 | 4860640 | eDNA + seine | Absent |
| JOSIES | Josies Brook | 2021 | 369686 | 4840580 | eDNA + seine | Absent |
| KIMBAL | Kimball Brook | 2021 | 341689 | 4887230 | eDNA + seine | Present |
| LITTLP-01 | Little Pond | 2021 | 350779 | 4884460 | eDNA | Unknown* |
| LITTLP-02 | Little Pond | 2021 | 350903 | 4884350 | eDNA | Unknown* |
| LITTLR | Little River | 2021 | 350489 | 4803790 | eDNA + seine | Absent |
| MARBRK | Marshall Brook | 2021 | 551554 | 4902250 | eDNA + seine | Absent |
| MARRPD-01 | Marr Pond | 2022 | 476034 | 4999620 | eDNA | Absent |
| MARRPD-02 | Marr Pond | 2022 | 476743 | 4999440 | eDNA | Absent |
| MEADBR | Meadow Brook | 2022 | 413718 | 4869180 | eDNA | Absent |
| MERRIL | Merrill Brook | 2022 | 408744 | 4855850 | eDNA | Absent |
| MOSQPD | Mosquito Pond | 2022 | 356574 | 4906770 | eDNA | Absent |
| MUDNO | Mud Pond | 2022 | 358926 | 4865650 | eDNA | Present |
| MUDSO | Mud Pond | 2022 | 348484 | 4830530 | eDNA | Absent |
| OCSACO | Old Course Saco River | 2021 | 346256 | 4884460 | eDNA + seine | Absent |
| OSSIPE | Ossipee River | 2021-2022 | 353209 | 4852140 | eDNA + seine | Present |
| OSSIPM | Ossipee River | 2021 | 343737 | 4850670 | eDNA + seine | Present |
| OSSIPR | Ossipee River | 2022 | 353408 | 4852070 | eDNA | Absent |
| OTTER-01 | Snake Pond | 2022 | 378927 | 4846550 | eDNA | Absent |
| OTTER-02 | Half Moon Pond | 2022 | 378321 | 4846660 | eDNA | Absent |
| PANTHR-01 | Tenny River | 2022 | 382062 | 4866260 | eDNA | Present |
| PANTHR-02 | Panther Pond | 2022 | 381929 | 4863540 | eDNA | Absent |
| PANTHR-03 | Panther Pond | 2022 | 383454 | 4864880 | eDNA | Absent |
| PISCAT | Piscataqua River | 2021 | 394376 | 4847570 | eDNA + seine | Absent |
| PISCDN | Piscataqua River | 2022 | 395387 | 4845110 | eDNA | Absent |
| PISCUP | Piscataqua River | 2022 | 394834 | 4850480 | eDNA | Absent |
| PRESBG | Presumpscot River | 2022 | 383487 | 4846940 | eDNA | Absent |
| PRESUM-01 | Presumpscot River | 2021-2022 | 383533 | 4845090 | eDNA + seine | Present |
| PRESUM-02 | Presumpscot River | 2021 | 383583 | 4845200 | eDNA + seine | Present |
| PROCPD-01 | Proctor Pond | 2021 | 356431 | 4900500 | eDNA + seine | Absent |
| PROCPD-02 | Proctor Pond | 2021 | 356592 | 4900260 | eDNA + seine | Absent |
| RACHEL | Spurwink River | 2022 | 396681 | 4827270 | eDNA | Absent |
| RANGE-01 | Middle Range Pond | 2022 | 389383 | 4877000 | eDNA | Absent |
| RANGE-02 | Middle Range Pond | 2022 | 388929 | 4874370 | eDNA | Absent |
| REDBRK | Red Brook | 2022 | 391701 | 4831260 | eDNA | Absent |
| RIDGEB | Unnamed brook | 2022 | 363812 | 4826610 | eDNA | Absent |
| ROYAL | Royal River | 2022 | 398071 | 4874310 | eDNA | Absent |
| SACONO-01 | Saco River | 2021 | 353424 | 4862040 | eDNA + seine | Absent |
| SACONO-02 | Saco River | 2021 | 353642 | 4862300 | eDNA + seine | Absent |
| SACONO-03 | Saco River | 2021, 2022 | 353523 | 4862370 | eDNA + seine | Present |
| SACOSO | Saco River | 2021 | 356715 | 4851970 | eDNA + seine | Absent |
| SEBAGO-01 | Songo River | 2021 | 373567 | 4863550 | eDNA + seine | Present |
| SEBAGO-03 | Sebago Lake | 2021 | 370327 | 4864980 | eDNA + seine | Absent |
| SEBAGO-04 | Sticky River | 2021 | 375361 | 4848550 | eDNA + seine | Absent |
| SEBAGO-06 | Sebago Lake | 2021 | 381712 | 4861730 | eDNA + seine | Absent |
| SFALLS | Salmon Falls River | 2021 | 345364 | 4796430 | eDNA + seine | Present |
| SHEPR | Shepards River | 2022 | 345344 | 4866430 | eDNA | Absent |
| SOKOLK-01 | Sokokis Lake | 2022 | 356133 | 4839980 | eDNA | Absent |
| SOKOLK-02 | Sokokis Lake | 2022 | 354643 | 4841400 | eDNA | Absent |
| SOKOLK-03 | Sokokis Lake | 2022 | 354682 | 4841370 | eDNA | Absent |
| SOPER | Soper Mill Brook | 2022 | 402191 | 4875470 | eDNA | Absent |
| SPECPD | Spectacle Ponds | 2021 | 346921 | 4853590 | eDNA + seine | Absent |
| STANPD-01 | Stanley Pond | 2021 | 348533 | 4854720 | eDNA + seine | Absent |
| STANPD-02 | Stanley Pond | 2021 | 348118 | 4854850 | eDNA + seine | Absent |
| STANPD-03 | Stanley Pond | 2021 | 347764 | 4855830 | Seine | Absent |
| SYMMES-01 | Symmes Pond | 2022 | 348917 | 4834440 | eDNA | Absent |
| SYMMES-02 | Symmes Pond | 2022 | 348864 | 4834450 | eDNA | Absent |
| TRAFPD-01 | Trafton Pond | 2021 | 348270 | 4856280 | eDNA + seine | Absent |
| TRAFPD-02 | Trafton Pond | 2021 | 347797 | 4856810 | Seine | Absent |
| WATBRK | Unnamed brook | 2021-2022 | 368055 | 4845460 | eDNA + seine | Absent |
| *Sample unusable (algae) or lost. | |  |  |  |  |  |

**Supplement B. Plant species and percent cover estimates included in the local habitat model (Model 1).**

**Table SB1**. Aquatic plant species included in the local habitat classification and regression tree (CART) analysis and the plant cover category and subcategory assigned to each species or group of plants. Plants in the Floating category did not have a subcategory (denoted by ‘n/a’). An asterisk (*) by a species’ name denotes a semi-aquatic or wetland species that we only included in analyses when a plant of that species was at least partially underwater and available as cover.

| **Plant code** | **Species name or description** | **Common name** | **Category** | **Subcategory** | **Comments** |
| --- | --- | --- | --- | --- | --- |
| ALNINC | *Alnus incana** | Gray alder | Emergent | Persistent emergent | Shrub |
| BIDBEC | *Bidens beckii* | Beck's water-marigold | Submerged | Complex leaf |  |
| BRASCH | *Brasenia schreberi* | Watershield | Floating | n/a |  |
| CALLSP | *Callitriche* spp. | Water-starworts | Submerged | Complex leaf | Simple leaves but usually grows in dense patches, not singly |
| CARESP | *Carex* spp.* | Sedges | Emergent | Persistent emergent |  |
| CEPOCC | *Cephalanthus occidentalis** | Buttonbush | Emergent | Persistent emergent | Shrub |
| CERASP | *Ceratophyllum* spp. (*C. demersum* or *C. echinatum*) | Hornworts | Submerged | Complex leaf |  |
| DULARU | *Dulichium arundinaceum** | Threeway sedge | Emergent | Broad leaf |  |
| ELATRI | *Elatine triandra* | Longstem waterwort | Submerged or Emergent | Simple leaf or broad leaf |  |
| ELEACI | *Eleocharis acicularis* | Needle spikesedge | Submerged | Mat-forming and grass-like |  |
| ELEROB | *Eleocharis robbinsii* | Robbins' spikerush | Emergent | Broad leaf |  |
| ELODSP | *Elodea* spp. | Waterweeds | Submerged | Complex leaf |  |
| ERIOSP | *Eriocaulon* spp. | Pipeworts | Submerged | Simple leaf | Grass-like but not mat-forming. |
| EQUISP | *Equisetum* spp.* | Horsetails | Emergent | Broad leaf |  |
| GRAAUR | *Gratiola aurea** | Golden hedge-hyssop | Submerged or Emergent | Simple leaf or broad leaf |  |
| GRASS | Grass spp. (including *Leersia oryzoides*, *Glyceria* spp.)* | Grass spp. | Emergent | Persistent emergent |  |
| ISOESP | *Isoetes* spp. | Quillworts | Submerged | Simple leaf |  |
| JUNCSP | *Juncus* spp. (*J. canadensis* or *J. effusus*)* | Rushes | Emergent | Persistent emergent |  |
| JUNMIL | *Juncus militaris* | Bayonet rush | Emergent | Persistent emergent |  |
| ILEVER | *Ilex verticillata** | Common winterberry | Emergent | Persistent emergent | Shrub |
| LEMNSP | *Lemna* spp. | Duckweeds | Floating | n/a |  |
| LUDPAL | *Ludwigia palustris* | Marsh primrose-willow | Submerged | Simple leaf |  |
| LYSTER | *Lysimachia terrestris** | Swamp candles | Emergent | Broad leaf |  |
| MOSS1 | Unknown | Possibly a moss | Submerged | Complex leaf | HIGHLK-02: Possibly an *Elodea* sp. |
| MOSS2 | Bryophyta sp. | Moss sp. | Submerged | Complex leaf | Found more in streams growing on rocks |
| MYRTEN | *Myriophyllum tenellum* | Slender watermilfoil | Submerged | Complex leaf |  |
| MYRISP | *Myriophyllum* sp. (including *M. heterophyllum*) | Watermilfoils | Submerged | Complex leaf |  |
| NAJASP | *Najas* spp. | Waternymphs | Submerged | Mat-forming and grass-like |  |
| NASOFF | *Nasturtium officinale* | Watercress | Submerged | Simple leaf |  |
| NUPVAR | *Nuphar variegata* | Varigated yellow pond-lily | Floating | n/a |  |
| NYMCOR | *Nymphoides cordata* | Little floatingheart | Floating | n/a |  |
| NYMODO | *Nymphaea odorata* | White waterlily | Floating | n/a |  |
| PERSSP | *Persicaria* spp.* | Smartweeds | Emergent | Broad leaf |  |
| PONCOR | *Pontederia cordata* | Pickerelweed | Emergent | Broad leaf |  |
| POTAMP | *Potamogeton amplifolius* | Largeleaf pondweed | Submerged | Simple leaf |  |
| POTEPI | *Potamogeton epihydrus* | Ribbonleaf pondweed | Submerged/Floating | Mat-forming and grass-like |  |
| POTGEM | *Potamogeton pusillus* | Small pondweed | Submerged | Mat-forming and grass-like |  |
| POTILL | *Potamogeton illinoensis* | Illinois pondweed | Submerged | Simple leaf |  |
| POTNAT | *Potamogeton natans* | Floating pondweed | Floating | n/a |  |
| POTOAK | *Potamogeton oakesianus* | Oakes' pondweed | Submerged | Simple leaf |  |
| POTPER | *Potamogeton perfoliatus* | Claspingleaf pondweed | Submerged | Simple leaf |  |
| POTROB | *Potamogeton robbinsii* | Robbins' pondweed | Submerged | Mat-forming and grass-like |  |
| SAGFIL | *Sagittaria filiformis* | Threadleaf arrowhead | Submerged | Simple leaf |  |
| SAGLAT | *Sagittaria latifolia** | Common arrowhead | Emergent | Broad leaf |  |
| SCHSUB | *Schoenoplectus subterminalis* | Swaying bulrush | Submerged | Mat-forming and grass-like |  |
| SCICYP | *Scirpus cyperinus** | Woolgrass | Emergent | Persistent emergent |  |
| SPARSP | *Sparganium* spp. | Bur-reeds | Emergent | Broad leaf |  |
| TYPHSP | *Typha* sp. (*T. angustifolia* or *T. latifolia*) * | Cattails | Emergent | Cattails |  |
| UTRINT | *Utricularia intermedia* | Flatleaf bladderwort | Submerged | Complex leaf |  |
| UTRISP | *Utricularia* sp. (including *Utricularia purpurea*) | Bladderworts | Submerged | Complex leaf |  |
| VACCSP | *Vaccinium* spp. * | Cranberries | Emergent | Persistent emergent | Small shrub |
| VALAME | *Vallisneria americana* | Watercelery | Submerged | Mat-forming and grass-like |  |
| ZANPAL | *Zannichellia palustris* | Horned pondweed | Submerged | Mat-forming and grass-like |  |


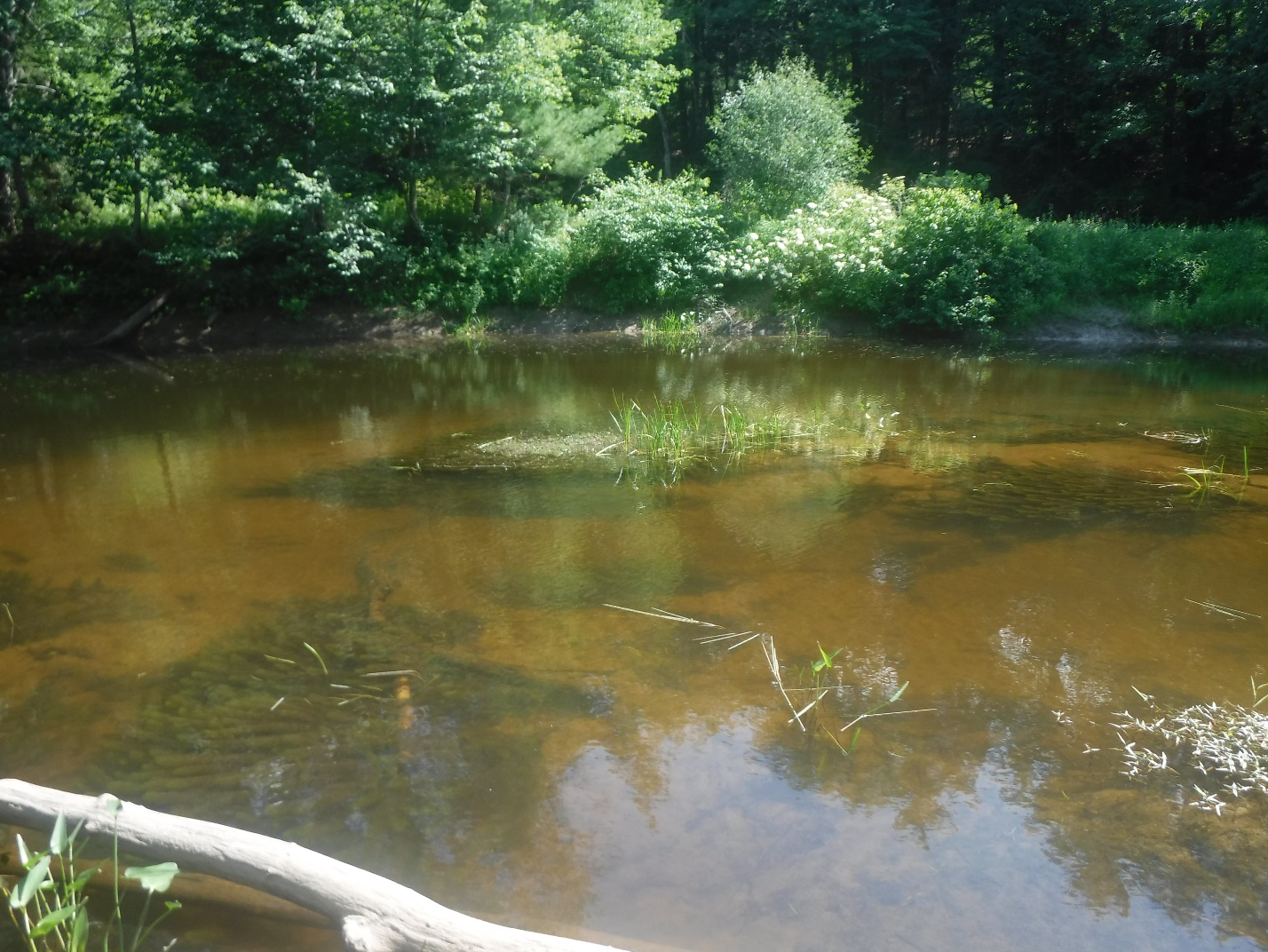


**Figure SB1**. Example of a Maine site (SFALLS; Table SA1) occupied by bridle shiner (*Notropis bifrenatus*) and dominated by sand substrate and open water with patches of submerged vegetation. We estimated that the site was dominated by 20% submerged vegetation cover, 10% emergent vegetation cover, 1% floating vegetation cover, and 69% open water (no vegetation). Submerged vegetation was dominated by complex-leaved species (90%, mostly *Myriophyllum* sp.) and simple-leaved species (10%). Emergent vegetation was dominated (100%) by the broad-leaved species *Persicaria amphibia* (water knotweed).


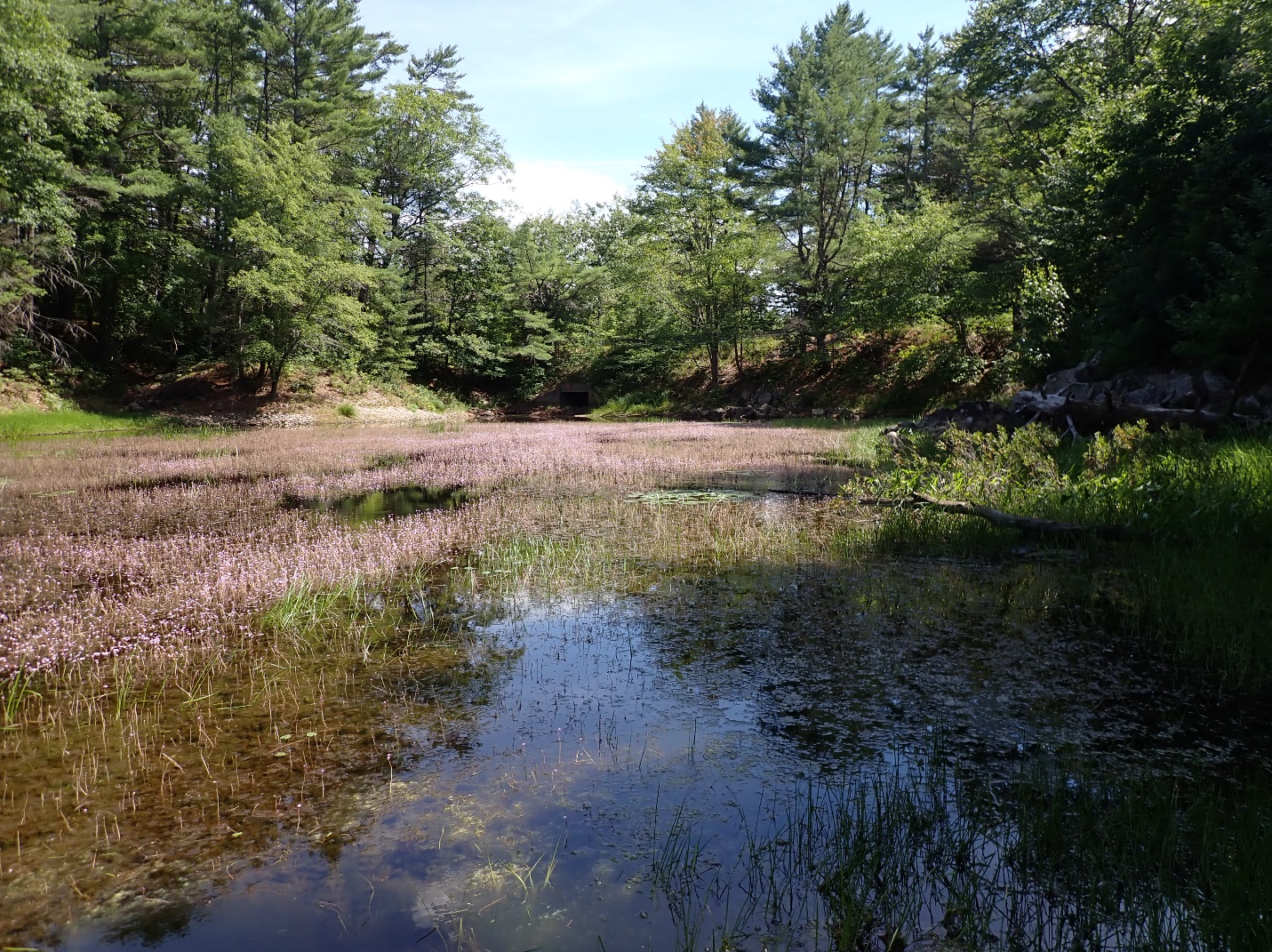


**Figure SB2**. Example of a Maine site (BUCKBR; Table SA1) occupied by bridle shiner (*Notropis bifrenatus*) and dominated by complex-leaved submerged vegetation. We estimated that the site was dominated by 85% submerged vegetation, 10% emergent vegetation, 5% floating vegetation, and 5% open water (no vegetation). We estimated that the submerged vegetation category was dominated by 80% complex-leaved species (mainly *Utricularia* spp.) and 20% mat-forming and grass-like species such as *Vallisneria americana*.


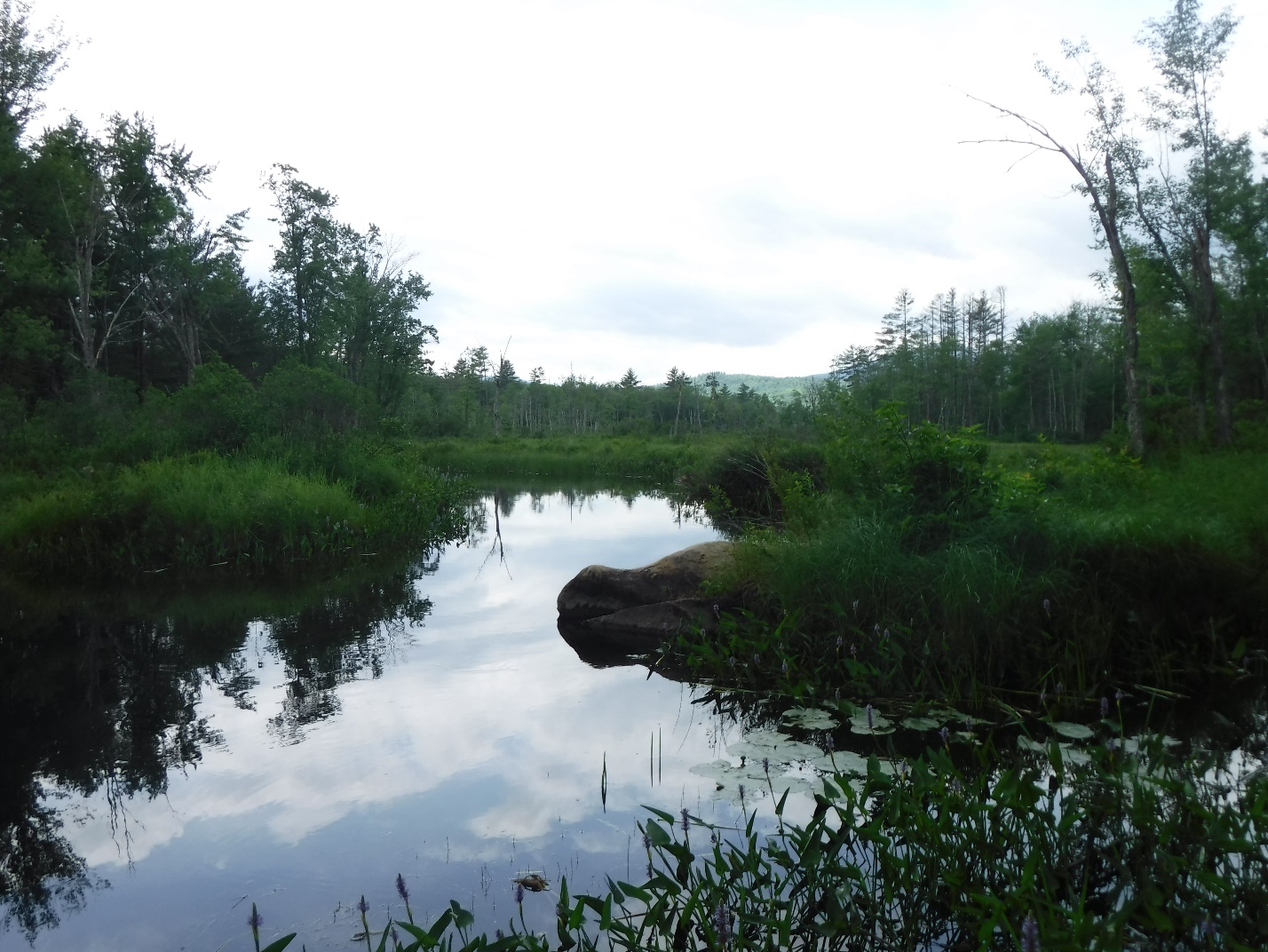

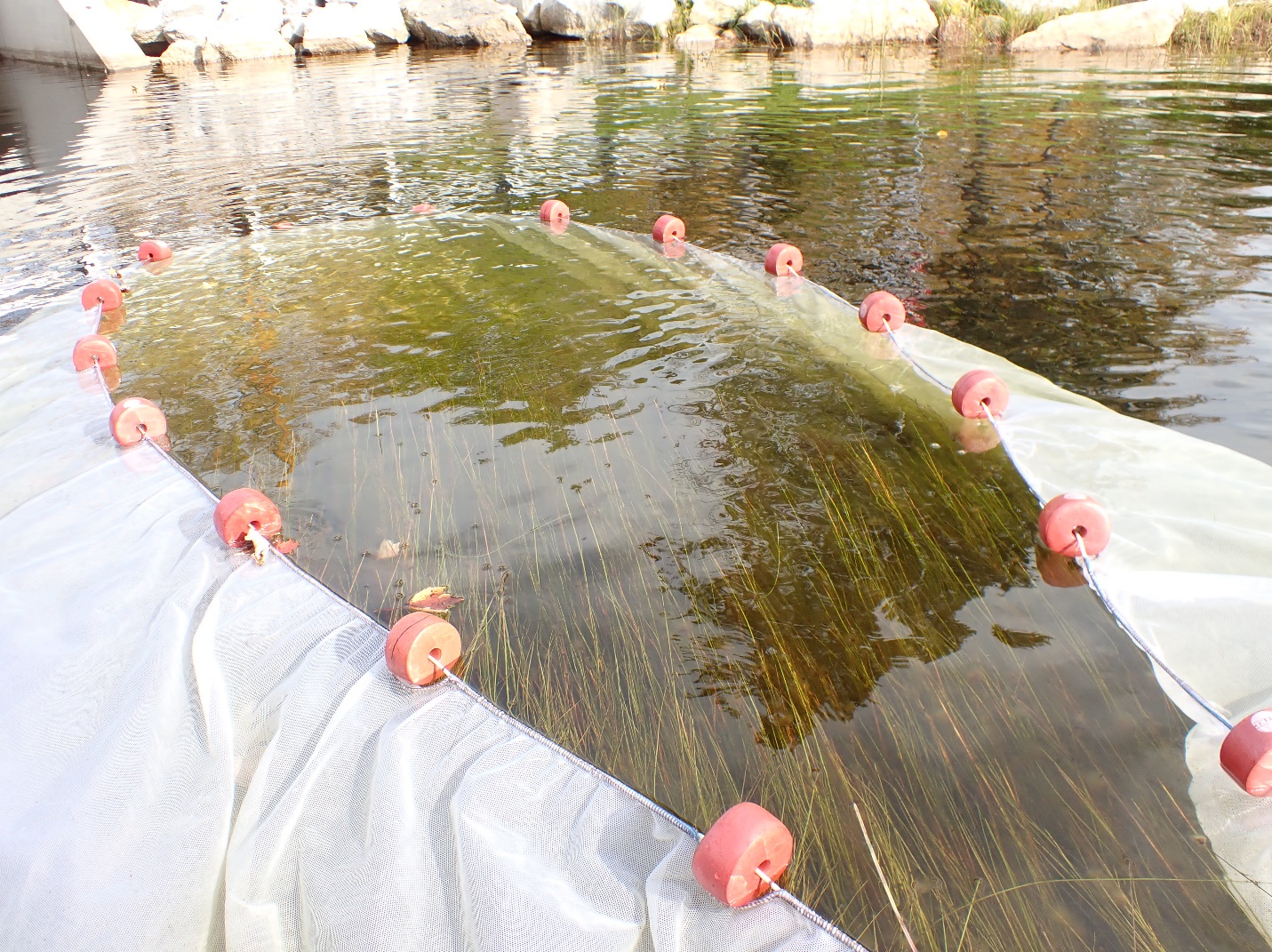


B

A

**Figure SB3**. Example of a Maine site (KIMBAL; Table SA1) occupied by bridle shiner (*Notropis bifrenatus*) and dominated by mat-forming and grass-like submerged vegetation. We estimated that the site was dominated by: A) 70% submerged vegetation cover, 20% emergent vegetation cover (mostly *Pontederia cordata*), 5% floating vegetation cover and 5% open water (no vegetation). B) The submerged vegetation was mostly (estimated 90%) made up of dense stands of *Schoenoplectus subterminalis*.

# **Supplement C. Maine local habitat model results (Model 1).**

**Table SC1**. Performance scores for the top four classification and regression tree (CART) models (Figure 2, Model 1) of local bridle shiner (*Notropis bifrenatus*) habitat in Maine.

|  | **Model 1a** | **Model 1b** | **Model 1c** | **Model 1d** |
| --- | --- | --- | --- | --- |
| Seed | 284 | 613 | 138 | 929 |
| Minsplit | 16 | 19 | 5 | 5 |
| Accuracy (training) | 0.860 | 0.895 | 0.947 | 0.965 |
| Accuracy (testing, unpruned) | 0.526 | 0.684 | 0.868 | 0.816 |
| Accuracy (testing, pruned) | 0.526 | 0.684 | 0.868 | 0.868 |
| Accuracy (full dataset) | 0.726 | 0.811 | 0.916 | 0.905 |
| Sensitivity (testing, pruned) | 0.714 | 0.714 | 0.429 | 0.286 |
| Sensitivity (full dataset) | 0.765 | 0.588 | 0.588 | 0.471 |
| Specificity (testing, pruned) | 0.484 | 0.677 | 0.968 | 1.000 |
| Specificity (full dataset) | 0.718 | 0.859 | 0.987 | 1.000 |
| Splitting variable 1 | subm | dom.substrate | subm | MYRISP |
| Splitting variable 2 | MYRISP | GRASS | MYRISP | TDS |
| Splitting variable 3 | . | PONCOR | prop.org.subst | elevation |


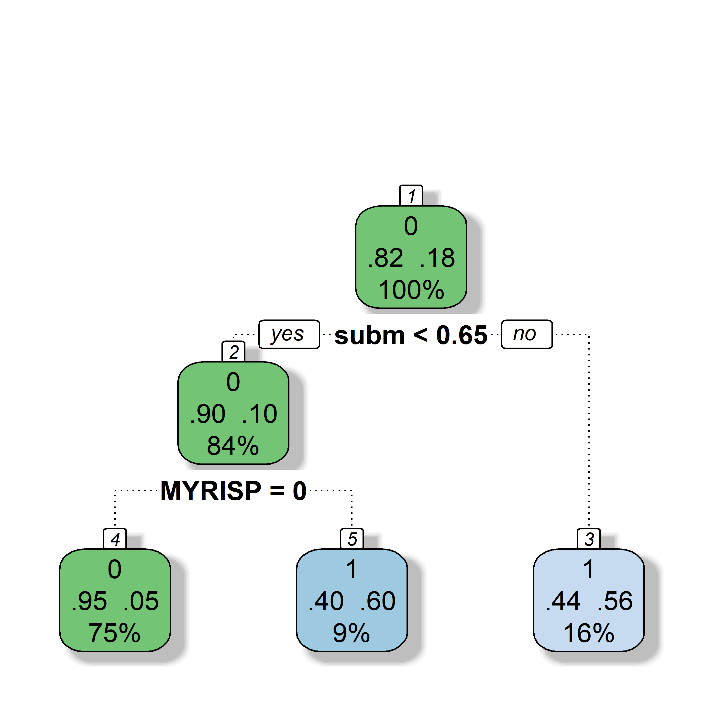


**Figure SC1.** Classification and regression tree (CART) model of bridle shiner (*Notropis bifrenatus*) presence-absence (2021-2022) as a response to 76 local habitat variables in Maine. Of the four top models, this model (Model 1a) had the highest sensitivity (76.5% true positive rate) but lowest overall classification accuracy (72.6%; Table SC1). Habitat variable abbreviations: “subm” refers to the proportion of the site dominated by submerged aquatic vegetation and “MYRISP” refers to the presence of *Myriophyllum* spp. (watermilfoils including *M. heterophyllum*; Table 1).


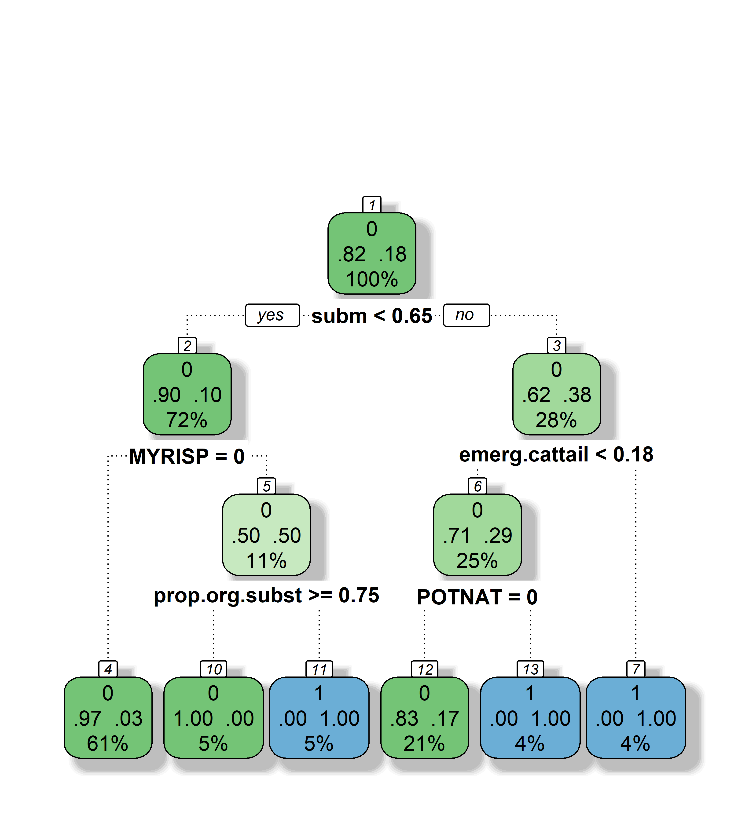


**Figure SC2**. Classification and regression tree (CART) model of bridle shiner (*Notropis bifrenatus*) presence-absence (2021-2022) as a response to 76 local habitat variables in Maine. Of the four top models, this model (Model 1c) had the highest classification accuracy (91.6%) but lowest sensitivity (58.8% true positive rate; Table SC1). Habitat variable abbreviations: “subm” refers to the proportion of the site dominated by submerged aquatic vegetation; “MYRISP” refers to the presence of *Myriophyllum* spp. (watermilfoils including *M. heterophyllum*); “prop.org.subst” refers to the proportion of the site dominated by organic substrates; “emerg.cattail” refers to the proportion of the site dominated by cattails (*Typha* spp.); “POTNAT” refers to the presence of *Potamogeton natans* (floating pondweed; Table 1).

**Table SC2**. Predictions of the top four classification and regression tree (CART) models of bridle shiner (*Notropis bifrenatus*) local habitat associations and the proportion of models that correctly classified occupancy at each site.

| **Site** | **Occupancy** | **Model 1a** | **Model 1b** | **Model 1c** | **Model 1d** | **Prop. correct** |
| --- | --- | --- | --- | --- | --- | --- |
| ANDROS | 0 | 0 | 0 | 0 | 0 | 1.00 |
| BARKER | 1 | 1 | 1 | 1 | 0 | 0.75 |
| BEARPD-01 | 0 | 1 | 0 | 0 | 0 | 0.75 |
| BEARPD-02 | 0 | 0 | 0 | 0 | 0 | 1.00 |
| BOOMBR | 0 | 0 | 0 | 0 | 0 | 1.00 |
| BRADPD-01 | 0 | 0 | 1 | 0 | 0 | 0.75 |
| BRADPD-02 | 0 | 1 | 1 | 0 | 0 | 0.50 |
| BROBRK | 0 | 0 | 0 | 0 | 0 | 1.00 |
| BUCKBR | 1 | 1 | 1 | 0 | 1 | 0.75 |
| BUFFBR | 0 | 0 | 0 | 0 | 0 | 1.00 |
| BURNPD-01 | 0 | 0 | 0 | 0 | 0 | 1.00 |
| BURNPD-02 | 0 | 0 | 0 | 0 | 0 | 1.00 |
| CARBRK | 0 | 0 | 0 | 0 | 0 | 1.00 |
| CHANBR | 0 | 0 | 0 | 0 | 0 | 1.00 |
| COLCPD-01 | 1 | 1 | 1 | 1 | 1 | 1.00 |
| COLCPD-02 | 0 | 1 | 0 | 0 | 0 | 0.75 |
| CRESLK-01 | 1 | 0 | 1 | 0 | 0 | 0.25 |
| CRESLK-02 | 1 | 1 | 1 | 0 | 0 | 0.50 |
| CRESLK-03 | 0 | 0 | 0 | 0 | 0 | 1.00 |
| CROOKN | 0 | 0 | 0 | 0 | 0 | 1.00 |
| CROOKR | 0 | 0 | 0 | 0 | 0 | 1.00 |
| CROOKS | 0 | 1 | 0 | 0 | 0 | 0.75 |
| DINGLY | 0 | 1 | 0 | 0 | 0 | 0.75 |
| DUCKIN | 0 | 1 | 1 | 0 | 0 | 0.50 |
| DUCKNO | 0 | 0 | 0 | 0 | 0 | 1.00 |
| EDDYBR | 0 | 0 | 0 | 0 | 0 | 1.00 |
| GRTBRK | 0 | 0 | 0 | 0 | 0 | 1.00 |
| GWORKB | 0 | 1 | 0 | 0 | 0 | 0.75 |
| GWORKN | 0 | 0 | 0 | 0 | 0 | 1.00 |
| GWORKS | 1 | 0 | 0 | 0 | 0 | 0.00 |
| HEATH-01 | 0 | 1 | 0 | 0 | 0 | 0.75 |
| HEATH-02 | 0 | 1 | 1 | 0 | 0 | 0.50 |
| HIGHLK-02 | 0 | 0 | 0 | 0 | 0 | 1.00 |
| HIGHLK-03 | 0 | 0 | 0 | 0 | 0 | 1.00 |
| HIGHLK-04 | 1 | 1 | 0 | 1 | 0 | 0.50 |
| INGALS-01 | 0 | 1 | 0 | 0 | 0 | 0.75 |
| INGALS-02 | 0 | 1 | 0 | 0 | 0 | 0.75 |
| JORDAN | 0 | 1 | 0 | 1 | 0 | 0.50 |
| JOSIES | 0 | 1 | 0 | 0 | 0 | 0.75 |
| KIMBAL | 1 | 1 | 0 | 1 | 1 | 0.75 |
| LITTLR | 0 | 0 | 0 | 0 | 0 | 1.00 |
| MARBRK | 0 | 0 | 0 | 0 | 0 | 1.00 |
| MARRPD-01 | 0 | 0 | 1 | 0 | 0 | 0.75 |
| MARRPD-02 | 0 | 0 | 1 | 0 | 0 | 0.75 |
| MEADBR | 0 | 0 | 0 | 0 | 0 | 1.00 |
| MERRIL | 0 | 0 | 0 | 0 | 0 | 1.00 |
| MOSQPD | 0 | 1 | 1 | 0 | 0 | 0.50 |
| MUDNO | 1 | 0 | 1 | 0 | 1 | 0.50 |
| MUDSO | 0 | 0 | 0 | 0 | 0 | 1.00 |
| OCSACO | 0 | 1 | 1 | 0 | 0 | 0.50 |
| OSSIPE | 1 | 0 | 1 | 0 | 0 | 0.25 |
| OSSIPM | 1 | 1 | 0 | 0 | 0 | 0.25 |
| OSSIPR | 0 | 0 | 0 | 0 | 0 | 1.00 |
| OTTER-01 | 0 | 0 | 0 | 0 | 0 | 1.00 |
| OTTER-02 | 0 | 0 | 0 | 0 | 0 | 1.00 |
| PANTHR-01 | 1 | 1 | 1 | 1 | 0 | 0.75 |
| PANTHR-02 | 0 | 0 | 1 | 0 | 0 | 0.75 |
| PANTHR-03 | 0 | 0 | 0 | 0 | 0 | 1.00 |
| PISCAT | 0 | 0 | 0 | 0 | 0 | 1.00 |
| PISCDN | 0 | 0 | 0 | 0 | 0 | 1.00 |
| PISCUP | 0 | 0 | 0 | 0 | 0 | 1.00 |
| PRESBG | 0 | 0 | 0 | 0 | 0 | 1.00 |
| PRESUM-01 | 1 | 1 | 1 | 1 | 1 | 1.00 |
| PRESUM-02 | 1 | 1 | 1 | 1 | 0 | 0.75 |
| PROCPD-01 | 0 | 1 | 0 | 0 | 0 | 0.75 |
| PROCPD-02 | 0 | 0 | 0 | 0 | 0 | 1.00 |
| RACHEL | 0 | 0 | 0 | 0 | 0 | 1.00 |
| RANGE-01 | 0 | 0 | 1 | 0 | 0 | 0.75 |
| RANGE-02 | 0 | 1 | 0 | 0 | 0 | 0.75 |
| REDBRK | 0 | 0 | 0 | 0 | 0 | 1.00 |
| RIDGEB | 0 | 0 | 0 | 0 | 0 | 1.00 |
| ROYAL | 0 | 0 | 0 | 0 | 0 | 1.00 |
| SACONO-01 | 0 | 0 | 0 | 0 | 0 | 1.00 |
| SACONO-02 | 0 | 0 | 0 | 0 | 0 | 1.00 |
| SACONO-03 | 1 | 1 | 0 | 1 | 1 | 0.75 |
| SACOSO | 0 | 0 | 0 | 0 | 0 | 1.00 |
| SEBAGO-01 | 1 | 1 | 0 | 1 | 1 | 0.75 |
| SEBAGO-03 | 0 | 1 | 0 | 0 | 0 | 0.75 |
| SEBAGO-04 | 0 | 1 | 1 | 0 | 0 | 0.50 |
| SEBAGO-06 | 0 | 0 | 0 | 0 | 0 | 1.00 |
| SFALLS | 1 | 1 | 0 | 1 | 1 | 0.75 |
| SHEPR | 0 | 0 | 0 | 0 | 0 | 1.00 |
| SOKOLK-01 | 0 | 0 | 0 | 0 | 0 | 1.00 |
| SOKOLK-02 | 0 | 1 | 0 | 0 | 0 | 0.75 |
| SOKOLK-03 | 0 | 1 | 0 | 0 | 0 | 0.75 |
| SOPER | 0 | 0 | 0 | 0 | 0 | 1.00 |
| SPECPD | 0 | 0 | 0 | 0 | 0 | 1.00 |
| STANPD-01 | 0 | 0 | 0 | 0 | 0 | 1.00 |
| STANPD-02 | 0 | 0 | 0 | 0 | 0 | 1.00 |
| STANPD-03 | 0 | 1 | 0 | 0 | 0 | 0.75 |
| SYMMES-01 | 0 | 0 | 0 | 0 | 0 | 1.00 |
| SYMMES-02 | 0 | 0 | 0 | 0 | 0 | 1.00 |
| TRAFPD-01 | 0 | 0 | 0 | 0 | 0 | 1.00 |
| TRAFPD-02 | 0 | 0 | 0 | 0 | 0 | 1.00 |
| WATBRK | 0 | 0 | 0 | 0 | 0 | 1.00 |

# **Supplement D. Bridle shiner survey sites in Maine and New Hampshire.**

**Table SD1**. Historical (1898-2008) and current (2009-2022) bridle shiner (*Notropis bifrenatus*) survey sites in Maine (ME) and New Hampshire (NH) used to determine occupied drainages for species distribution model. Some sites are listed more than once: these reflect spatial or temporal replicates taken from different areas of the site (e.g., many sites in Maine).

| **Site Name** | **Water Body** | **Occupancy** | **Year** | **State** | **Easting** | **Northing** | **Point Type** | **Source** |
| --- | --- | --- | --- | --- | --- | --- | --- | --- |
| 19860610-Baboosic Brook-Merrimack-EFISH | Baboosic Brook | Historical presence | 1986 | NH | 292838 | 4751245 | Digitized from NH Fish survey map (StoryMap) | 1 |
| 19870609-Baboosic Brook-Amherst-EFISH | Baboosic Brook | Historical presence | 1987 | NH | 289391 | 4752970 | Digitized from NH Fish survey map (StoryMap) | 1 |
| 20080717-830-EBTJV-unnamed stream-Madison-EFISH | Banfield Brook | Historical presence | 2008 | NH | 327736 | 4866768 | GPS survey coordinates | 2 |
| BARKER | Barker Pond | Historical presence | 1938 | ME | 358811 | 4861900 | Center of waterbody: no precise location known | 3, 4 |
| GWORKB | Bauneg Beg Pond | Historical presence | 1937 | ME | 359032 | 4801911 | Upstream stream survey point. | 4 |
| BEARPD | Bear Pond | Historical presence | 1939 | ME | 362552 | 4889916 | Center of waterbody: no precise location known | 3, 4 |
| 19870609-Beaver Brook-Amherst-EFISH | Beaver Brook | Historical presence | 1987 | NH | 286246 | 4748446 | Digitized from NH Fish survey map (StoryMap) | 1 |
| 20070628-800-NHDES-Berrys River-Strafford-EFISH | Berrys River | Historical presence | 2007 | NH | 332695 | 4794644 | GPS survey coordinates | 2 |
| 19850718-Branch River-Wakefield-EFISH | Branch River | Historical presence | 1985 | NH | 336507 | 4820782 | Digitized from NH Fish survey map (StoryMap) | 1 |
| BURNPD | Burnt Meadow Pond | Historical presence | 1939 | ME | 348433 | 4865375 | Center of waterbody: no precise location known | 3, 4 |
| 1947-Canobie Lake-Salem/Windham | Canobie Lake | Historical presence | Unknown | NH | 315568 | 4740615 | Lake centroid | 5 |
| Chaffin Pond | Chaffin Pond | Historical presence | 1899 | ME | 383978 | 4856036 | Center of waterbody: no precise location known | 6 |
| 19850711-Cocheco River-Farmington-EFISH | Cocheco River | Historical presence | 1985 | NH | 333084 | 4805888 | Digitized from NH Fish survey map (StoryMap) | 1 |
| 20050812-1220-WAP-Coffin Brook-Alton-SEINE | Coffin Brook | Historical presence | 2005 | NH | 319310 | 4810310 | GPS survey coordinates | 2 |
| CRESLK | Crescent Lake | Historical presence | 1939 | ME | 383196 | 4869639 | Center of waterbody: no precise location known | 4 |
| CRESLK-02 | Crescent Lake | Historical presence | 1899 | ME | 383847 | 4869327 | Approximated from description of survey location | 6 |
| CROOKR | Crooked River | Historical presence | 1937 | ME | 373969 | 4873045 | Upstream stream survey point. | 4 |
| 19850709-Ela River-Farmington-EFISH | Ela River | Historical presence | 1985 | NH | 329943 | 4807156 | Digitized from NH Fish survey map (StoryMap) | 1 |
| 20060626-1155-WAP-Exeter River-Fremont-SEINE | Exeter River | Historical presence | 2006 | NH | 327050 | 4759241 | GPS survey coordinates | 2 |
| GWORKN | Great Works River | Historical presence | 1937 | ME | 357517 | 4805701 | Upstream stream survey point. | 4 |
| GWORKS | Great Works River | Historical presence | 1937 | ME | 358948 | 4797538 | Upstream stream survey point. | 4 |
| UNK-Heads Pond-Hooksett | Heads Pond | Historical presence | Unknown | NH | 301768 | 4774843 | Pond centroid | 5 |
| HIGHLK | Highland Lake | Historical presence | 1938 | ME | 360853 | 4882237 | Center of waterbody: no precise location known | 3, 4 |
| 20051012-1345-WAP-Isinglass River-Barrington-SEINE | Isinglass River | Historical presence | 2005 | NH | 333201 | 4789549 | GPS survey coordinates | 2 |
| 20060808-800-NHDES-Isinglass River-Barrington-EFISH | Isinglass River | Historical presence | 2006 | NH | 337298 | 4790038 | GPS survey coordinates | 2 |
| 20050803-1100-WAP-Jones Brook-Middleton-SEINE | Jones Brook | Historical presence | 2005 | NH | 334893 | 4816200 | GPS survey coordinates | 2 |
| Josias River | Josias River | Historical presence | Unknown | ME | 366676 | 4787109 | Survey coordinates | 7 |
| JOSIES | Josies Brook | Historical presence | 1937 | ME | 369739 | 4840524 | Upstream stream survey point. | 4 |
| KIMBAL | Kimball Brook | Historical presence | 2010 | ME | 341705 | 4887236 | Upstream stream survey point. | 4 |
| 1947-Winnipesaukee Lake-Fish Cove-Meredith | Lake Winnipesaukee | Historical presence | 1938 | NH | 303342 | 4835248 | Digitized coordinates: northern end of cove | 5 |
| 20060821-1220-WAP-Lake Winnipesaukee-Moultonborough-SEINE | Lake Winnipesaukee | Historical presence | 2006 | NH | 303164 | 4842575 | GPS survey coordinates | 2 |
| 1947-Winnisquam Lake-Sanbornton/Laconia/Belmont | Lake Winnisquam | Historical presence | Unknown | NH | 297443 | 4823795 | Lake centroid | 5 |
| 19850911-Lamprey River-Lee-EFISH | Lamprey River | Historical presence | 1985 | NH | 336628 | 4772908 | Digitized from NH Fish survey map (StoryMap) | 1 |
| 19980824-Lamprey River-Lee-EFISH | Lamprey River | Historical presence | 1998 | NH | 320173 | 4768526 | Digitized from NH Fish survey map (StoryMap) | 1 |
| 19980827-Lamprey River-Raymond-EFISH | Lamprey River | Historical presence | 1998 | NH | 320173 | 4768526 | Digitized from NH Fish survey map (StoryMap) | 1 |
| 20030825-Lamprey River-East of Wadleigh Falls Rd-Lee-EBOAT | Lamprey River | Historical presence | 2003 | NH | 336982 | 4772807 | Digitized from NH Fish survey map (StoryMap) | 1 |
| 20030825-Lamprey River-West of Wadleigh Falls Rd-Lee-EBOAT | Lamprey River | Historical presence | 2003 | NH | 335878 | 4772337 | Digitized from NH Fish survey map (StoryMap) | 1 |
| 20030826-Lamprey River-ReachCode01060003001832-Lee-EBOAT | Lamprey River | Historical presence | 2003 | NH | 337279 | 4772336 | Digitized from NH Fish survey map (StoryMap) | 1 |
| 20030826-Lamprey River-ReachCode01060003001872-Lee-EBOAT | Lamprey River | Historical presence | 2003 | NH | 337664 | 4772433 | Digitized from NH Fish survey map (StoryMap) | 1 |
| 20030827-Lamprey River-Lee-EBOAT | Lamprey River | Historical presence | 2003 | NH | 337217 | 4773680 | Digitized from NH Fish survey map (StoryMap) | 1 |
| 20060626-1030-WAP-Lamprey River-Raymond-SEINE | Lamprey River | Historical presence | 2006 | NH | 319632 | 4768987 | GPS survey coordinates | 2 |
| UNK-Lamprey River-Bunker Pond-Epping | Lamprey River | Historical presence | Unknown | NH | 326528 | 4767262 | Digitized coordinates from 1992 aerial imagery (Google Earth) | 5 |
| LITTLP | Little Pond | Historical presence | 1999 | ME | 350765 | 4884330 | Center of waterbody: no precise location known | 4 |
| LITTLR | Little River | Historical presence | 1937 | ME | 350431 | 4803832 | Upstream stream survey point. | 4 |
| Little Sebago Lake | Little Sebago Lake | Historical presence | 1898 | ME | 386662 | 4859630 | Center of waterbody: no precise location known | 6 |
| 20010620-Little Suncook River-Epsom-EFISH | Little Suncook River | Historical presence | 2001 | NH | 310797 | 4788057 | Digitized from NH Fish survey map (StoryMap) | 1 |
| 20050801-1330-WAP-Little Suncook River-Epsom-SEINE | Little Suncook River | Historical presence | 2005 | NH | 312316 | 4788272 | GPS survey coordinates | 2 |
| 1947-Lower Suncook Lake-Barnstead | Lower Suncook Lake | Historical presence | Unknown | NH | 316474 | 4804418 | Lake centroid | 5 |
| 19980827-Mad River-Farmington-EFISH | Mad River | Historical presence | 1998 | NH | 332108 | 4805965 | Point in between Mad River and Ela River: snapped to Mad River flowline | 2 |
| MARBRK | Marshall Brook | Historical presence | 1992 | ME | 551554 | 4902250 | Approximated from description of survey location | 8 |
| 19980825-North River-Nottingham-EFISH | North River | Historical presence | 1998 | NH | 328403 | 4781123 | Digitized from NH Fish survey map (StoryMap) | 1 |
| OCSACO | Old Course Saco River | Historical presence | 1939 | ME | 346205 | 4884581 | Upstream stream survey point. | 4 |
| OSSIPE | Ossipee River | Historical presence | 1939 | ME | 353118 | 4852028 | Upstream stream survey point. | 4 |
| OSSIPM | Ossipee River | Historical presence | 1939 | ME | 344008 | 4850493 | Upstream stream survey point. | 4 |
| OSSIPW | Ossipee River | Historical presence | 1939 | ME | 340547 | 4850908 | Upstream stream survey point. | 4 |
| 19460618-Harrington-Oyster River-Mill Pond-Durham | Oyster River | Historical presence | 1946 | NH | 343760 | 4777090 | Point in approximate center of impoundment: exact location unknown | 5, 9 |
| 19850705-Oyster River-Lee-EFISH | Oyster River | Historical presence | 1985 | NH | 340160 | 4779151 | Digitized from NH Fish survey map (StoryMap) | 1 |
| JORDAN | Panther Run | Historical presence | 1939 | ME | 382440 | 4860683 | Upstream stream survey point. | 4 |
| 20060627-1400-WAP-Pemigewasset Lake-New Hampton-SEINE | Pemingewasset Lake | Historical presence | 2006 | NH | 289875 | 4832820 | GPS survey coordinates | 2 |
| 20080521-1100-HERB-Pemigewasset Lake-New Hampton-EBOAT | Pemingewasset Lake | Historical presence | 2008 | NH | 290000 | 4832803 | GPS survey coordinates | 2 |
| PISCAT | Piscataqua River | Historical presence | 2002 | ME | 394861 | 4846070 | Upstream stream survey point. | 4 |
| 19850626-Piscataquog River-Goffstown-EFISH | Piscataquog River | Historical presence | 1985 | NH | 293864 | 4765225 | Digitized from NH Fish survey map (StoryMap) | 1 |
| 19850701-Piscataquog River-Goffstown-EFISH | Piscataquog River | Historical presence | 1985 | NH | 293564 | 4765251 | Digitized from NH Fish survey map (StoryMap) | 1 |
| 1947-Pleasant Lake-Deerfield | Pleasant Lake | Historical presence | Unknown | NH | 316353 | 4784453 | Point at northern end of cove: no precise survey location known | 5 |
| 20050707-1200-WAP-Powwow River-South Hampton-SEINE | Powwow River | Historical presence | 2005 | NH | 337348 | 4748991 | GPS survey coordinates | 2 |
| PRESUM | Presumpscot River | Historical presence | 2006 | ME | 383425 | 4845348 | Upstream stream survey point. | 4 |
| PROCPD | Proctor Pond | Historical presence | 1992 | ME | 356556 | 4900484 | GPS survey coordinates | 10 |
| 20050919-1225-WAP-Purity Lake-Madison-SEINE | Purity Lake | Historical presence | 2005 | NH | 332148 | 4858566 | GPS survey coordinates | 2 |
| 19850702-Riddle Brook-Bedford-EFISH | Riddle Brook | Historical presence | 1985 | NH | 292452 | 4759408 | Digitized from NH Fish survey map (StoryMap) | 1 |
| SACONO | Saco River | Historical presence | 1939 | ME | 352778 | 4862362 | Upstream stream survey point. | 4 |
| SACOSO | Saco River | Historical presence | 1939 | ME | 356749 | 4852010 | Upstream stream survey point. | 4 |
| SFALLS | Salmon Falls River | Historical presence | 1937 | ME | 345383 | 4796446 | Upstream stream survey point. | 4 |
| Kettle Cove | Sebago Lake | Historical presence | Unknown | ME | 376212 | 4864764 | Approximated from description of survey location | 6 |
| SEBAGO | Sebago Lake | Historical presence | 1898 | ME | 373319 | 4857226 | Center of waterbody: no precise location known | 3, 4 |
| SEBAGO-01 | Sebago Lake | Historical presence | 1900 | ME | 373371 | 4863214 | Approximated from description of survey location | 4, 6 |
| 1947-Shadow Lake-Salem/Windham | Shadow Lake | Historical presence | Unknown | NH | 316939 | 4743138 | Lake centroid | 5 |
| 20000711-Shaker Brook-Loudon-EFISH | Shaker Branch | Historical presence | 2000 | NH | 298262 | 4800214 | Digitized from NH Fish survey map (StoryMap) | 1 |
| SOKOLK | Sokokis Lake | Historical presence | 1955 | ME | 355058 | 4840728 | Center of waterbody: no precise location known | 4 |
| 20050616-1400-WAP-Soucook River-Loudon-SEINE | Soucook River | Historical presence | 2005 | NH | 299721 | 4795557 | GPS survey coordinates | 2 |
| 20060510-1500-WAP-Soucook River-Loudon-SEINE | Soucook River | Historical presence | 2006 | NH | 299977 | 4795896 | GPS survey coordinates | 2 |
| 20080812-1000-WAP-Soucook River-Canterbury-SEINE | Soucook River | Historical presence | 2008 | NH | 301052 | 4806983 | GPS survey coordinates | 2 |
| SPECPD | Spectacle Pond | Historical presence | 1938 | ME | 346858 | 4853544 | Approximated from description of survey location | 4 |
| STANPD | Stanley Pond | Historical presence | 1938 | ME | 348269 | 4855353 | Center of waterbody: no precise location known | 3, 4 |
| 19380616-Harrington-Suncook River-Channel between the Suncook Ponds-Barnstead | Suncook River | Historical presence | 1938 | NH | 315824 | 4805475 | Point in approximate center of channel: exact location unknown | 11 |
| 20050801-1130-WAP-Suncook River-Epsom-SEINE | Suncook River | Historical presence | 2005 | NH | 306455 | 4785958 | GPS survey coordinates | 2 |
| 20050812-1200-WAP-Suncook River-Pembroke-SEINE | Suncook River | Historical presence | 2005 | NH | 304380 | 4781371 | GPS survey coordinates | 2 |
| Tenny River | Tenny River | Historical presence | 1906 | ME | 382430 | 4866833 | Center of waterbody: no precise location known | 6 |
| TRAFPD | Trafton Pond | Historical presence | 1938 | ME | 347996 | 4856501 | Center of waterbody: no precise location known | 3, 4 |
| 20050919-1515-WAP-Trout Pond-Freedom-SEINE | Trout Pond | Historical presence | 2005 | NH | 328826 | 4856328 | GPS survey coordinates | 2 |
| BLANBR | Unnamed brook | Historical presence | 2002 | ME | 396180 | 4852403 | Upstream stream survey point. | 4 |
| BOOMBR | Unnamed brook | Historical presence | 2010 | ME | 377131 | 4819868 | Upstream stream survey point. | 4 |
| WATBRK | Unnamed brook | Historical presence | 2010 | ME | 368058 | 4845444 | Upstream stream survey point. | 4 |
| 20050803-1345-WAP-Cocheco River-Farmington-SEINE | Waldron Mill Pond | Historical presence | 2005 | NH | 331081 | 4808093 | GPS survey coordinates | 2 |
| 1947-Wheelwright Pond-Lee | Wheelwright Pond | Historical presence | 1947 | NH | 336471 | 4778033 | Center of waterbody: no precise location known | 5, 9 |
| BROBRK | Browns Brook | Historical presence (likely misidentified) | 2007 | ME | 554512 | 4967921 | GPS survey coordinates | 4 |
| MARRPD | Marr Pond | Historical presence (likely misidentified) | 1960 | ME | 476293 | 4999425 | Center of waterbody: no precise location known | 4 |
| Meddybemps Lake | Meddybemps Lake | Historical presence (likely misidentified) | 1997 | ME | 627061 | 4992143 | Center of waterbody: no precise location known | 12 |
| Sunday Pond | Sunday Pond | Historical presence (likely misidentified) | 1976 | ME | 345573 | 4962472 | Center of waterbody: no precise location known | 12 |
| West Grand Lake | West Grand Lake | Historical presence (likely misidentified) | 1952 | ME | 590071 | 5009684 | Center of waterbody: no precise location known | 12 |
| BARKER | Barker Pond | Current presence | 2021 | ME | 359060 | 4860985 | Mean center point of seine and/or eDNA sample replicates collected at this location | 13 |
| 20190701-1130-BS-unnamed stream-Ossipee-DIPNET | Beech River | Current presence | 2019 | NH | 324576 | 4840402 | GPS survey coordinates | 2 |
| 20170913-1300-BS-Little Suncook River-Epsom-DIPNET | Bixby Pond | Current presence | 2017 | NH | 312215 | 4788215 | GPS survey coordinates | 2 |
| 20090629-1200-IMPOUND-Black Brook-Manchester-EFISH | Black Brook | Current presence | 2009 | NH | 298097 | 4764887 | GPS survey coordinates | 2 |
| 20120802-1300-BS-Branch River-Milton-DIPNET | Branch River | Current presence | 2012 | NH | 340367 | 4813998 | Mean center point of 2+ surveys at this location | 2 |
| 20200818-1230-BS-Branch River-Milton-DIPNET | Branch River | Current presence | 2020 | NH | 339216 | 4815551 | GPS survey coordinates | 2 |
| BUCKBR | Buck Meadow Brook | Current presence | 2022 | ME | 350873 | 4869437 | Mean center point of eDNA sample replicates collected at this location | 13 |
| 20140908-800-BS-Cocheco River-Farmington-DIPNET | Cocheco River | Current presence | 2014 | NH | 330772 | 4809005 | GPS survey coordinates | 2 |
| 20140929-1000-BS-Coffin Brook-Alton-DIPNET | Coffin Brook | Current presence | 2014 | NH | 319744 | 4809648 | GPS survey coordinates | 2 |
| 20140929-1200-BS-Coffin Brook-Alton-DIPNET | Coffin Brook | Current presence | 2014 | NH | 321001 | 4810064 | GPS survey coordinates | 2 |
| 20140929-1300-BS-Coffin Brook-Alton-DIPNET | Coffin Brook | Current presence | 2014 | NH | 321740 | 4810226 | GPS survey coordinates | 2 |
| 20140929-800-BS-Coffin Brook-Alton-DIPNET | Coffin Brook | Current presence | 2014 | NH | 319217 | 4810695 | GPS survey coordinates | 2 |
| COLCPD-01 | Colcord Pond | Current presence | 2022 | ME | 342841 | 4855433 | Mean center point of eDNA sample replicates collected at this location | 13 |
| 20150602-Connecticut River-Lebanon-EBOAT | Connecticut River | Current presence | 2015 | NH | 233592 | 4840683 | Digitized from NH Fish survey map (StoryMap) | 1 |
| 20150728-Connecticut River-Lyme-EBOAT | Connecticut River | Current presence | 2015 | NH | 242281 | 4852018 | Digitized from NH Fish survey map (StoryMap) | 1 |
| 20150730-Connecticut River-Hanover-EBOAT | Connecticut River | Current presence | 2015 | NH | 240077 | 4849457 | Digitized from NH Fish survey map (StoryMap) | 1 |
| 20150915-Connecticut River-Charlestown-EBOAT | Connecticut River | Current presence | 2015 | NH | 219944 | 4785825 | Digitized from NH Fish survey map (StoryMap) | 1 |
| 20200806-1000-BS-Cooks Pond-Madison-DIPNET | Cooks Pond | Current presence | 2020 | NH | 326549 | 4859109 | GPS survey coordinates | 2 |
| CRESLK-02 | Crescent Lake | Current presence | 2022 | ME | 383800 | 4869379 | Mean center point of eDNA sample replicates collected at this location | 13 |
| 20130718-1000-BS-Crystal Lake-Gilmanton-DIPNET | Crystal Lake | Current presence | 2013 | NH | 312049 | 4813773 | GPS survey coordinates | 2 |
| 20210623-1300-BS-Crystal Lake-Gilmanton -DIPNET | Crystal Lake | Current presence | 2021 | NH | 313566 | 4811089 | GPS survey coordinates | 2 |
| 20140827-1000-BS-Exeter River-Fremont-DIPNET | Exeter River | Current presence | 2014 | NH | 329049 | 4758827 | GPS survey coordinates | 2 |
| 20140827-800-BS-Exeter River-Fremont-DIPNET | Exeter River | Current presence | 2014 | NH | 326845 | 4759905 | GPS survey coordinates | 2 |
| 20171003-1230-BS-Exeter River-Fremont-DIPNET | Exeter River | Current presence | 2017 | NH | 325949 | 4760503 | GPS survey coordinates | 2 |
| 20171003-930-BS-Exeter River-Brentwood-DIPNET | Exeter River | Current presence | 2017 | NH | 329767 | 4759146 | GPS survey coordinates | 2 |
| 20171018-1000-BS-Exeter River-Brentwood-DIPNET | Exeter River | Current presence | 2017 | NH | 336793 | 4759418 | GPS survey coordinates | 2 |
| 20210721-1100-BS-Exeter River-Exeter-DIPNET | Exeter River | Current presence | 2021 | NH | 341678 | 4758791 | GPS survey coordinates | 2 |
| 20220823-1430-BS-Exeter River-Chester-DIPNET | Exeter River | Current presence | 2022 | NH | 320961 | 4759832 | GPS survey coordinates | 2 |
| 20130806-1200-BS-Garland Pond-Moultonborough-DIPNET | Garland Pond | Current presence | 2013 | NH | 306200 | 4846808 | GPS survey coordinates | 2 |
| 20190701-1030-BS-Garland Pond-Ossipee-DIPNET | Garland Pond | Current presence | 2019 | NH | 323622 | 4840928 | GPS survey coordinates | 2 |
| 20220711-1330-BS-Garland Pond-Moultonborough-DIPNET | Garland Pond | Current presence | 2022 | NH | 305852 | 4846897 | GPS survey coordinates | 2 |
| GWORKS | Great Works River | Current presence | 2021 | ME | 358932 | 4797487 | Mean center point of seine and/or eDNA sample replicates collected at this location | 13 |
| 20170926-1200-BS-Harper Brook tributary-New Hampton-DIPNET | Harper Brook | Current presence | 2017 | NH | 289303 | 4833369 | Mean center point of 2+ surveys at this location | 2 |
| HIGHLK-04 | Highland Lake | Current presence | 2021 | ME | 359880 | 4884753 | Mean center point of seine and/or eDNA sample replicates collected at this location | 13 |
| HIGHLK-04 | Highland Lake | Current presence | 2021 | ME | 359754 | 4884799 | Mean center point of seine and/or eDNA sample replicates collected at this location | 13 |
| 20130723-1300-BS-Isinglass River-Barrington-DIPNET | Isinglass River | Current presence | 2013 | NH | 332103 | 4788900 | GPS survey coordinates | 2 |
| 20200707-1000-BS-Isinglass River-Barrington-DIPNET | Isinglass River | Current presence | 2020 | NH | 330813 | 4789621 | GPS survey coordinates | 2 |
| 20200707-1530-BS-Isinglass River-Barrington-DIPNET | Isinglass River | Current presence | 2020 | NH | 330310 | 4789780 | GPS survey coordinates | 2 |
| 20120801-1030-BS-Jones Brook-Middleton-DIPNET | Jones Brook | Current presence | 2012 | NH | 333250 | 4816854 | GPS survey coordinates | 2 |
| 20120801-1200-BS-Jones Brook-Middleton-DIPNET | Jones Brook | Current presence | 2012 | NH | 333751 | 4817084 | GPS survey coordinates | 2 |
| 20120801-1330-BS-Jones Brook-Middleton-DIPNET | Jones Brook | Current presence | 2012 | NH | 332844 | 4817220 | GPS survey coordinates | 2 |
| 20120801-900-BS-Jones Brook-Middleton-DIPNET | Jones Brook | Current presence | 2012 | NH | 334802 | 4816368 | GPS survey coordinates | 2 |
| 20130812-1000-BS-Jones Brook-Milton-DIPNET | Jones Brook | Current presence | 2013 | NH | 337824 | 4813437 | GPS survey coordinates | 2 |
| 20130812-1200-BS-Jones Brook-Milton-DIPNET | Jones Brook | Current presence | 2013 | NH | 335218 | 4815056 | GPS survey coordinates | 2 |
| KIMBAL | Kimball Brook | Current presence | 2021 | ME | 341695 | 4887242 | Mean center point of seine and/or eDNA sample replicates collected at this location | 13 |
| 20180709-1000-BS-Kanasatka Lake-Moultonborough-DIPNET | Lake Kanasatka | Current presence | 2018 | NH | 303025 | 4843212 | GPS survey coordinates | 2 |
| 20180709-1130-BS-Kanasatka Lake-Moultonborough-DIPNET | Lake Kanasatka | Current presence | 2018 | NH | 302156 | 4844502 | GPS survey coordinates | 2 |
| 20180709-1300-BS-Kanasatka Lake-Moultonborough-DIPNET | Lake Kanasatka | Current presence | 2018 | NH | 301199 | 4844667 | GPS survey coordinates | 2 |
| 20210713-1100-BS-Snake River-New Hampton-DIPNET | Lake Waukewan | Current presence | 2021 | NH | 294736 | 4837713 | GPS survey coordinates | 2 |
| 20140909-1200-BS-Wentworth Lake-Wolfeboro-DIPNET | Lake Wentworth | Current presence | 2014 | NH | 327301 | 4830530 | GPS survey coordinates | 2 |
| 20140909-800-BS-Wentworth Lake-Wolfeboro-DIPNET | Lake Wentworth | Current presence | 2014 | NH | 323743 | 4828392 | GPS survey coordinates | 2 |
| 20210723-1400-BS-Wickwas Lake-Meredith-DIPNET | Lake Wicwas | Current presence | 2021 | NH | 293622 | 4832449 | GPS survey coordinates | 2 |
| 20090604-1100-BS-Lake Winnipesaukee-Moultonborough-SEINE | Lake Winnipesaukee | Current presence | 2009 | NH | 307972 | 4844033 | Mean center point of 2+ surveys at this location | 2 |
| 20090604-1130-BS-Lake Winnipesaukee-Moultonborough-SEINE | Lake Winnipesaukee | Current presence | 2009 | NH | 307997 | 4844158 | GPS survey coordinates | 2 |
| 20090604-1200-BS-Lake Winnipesaukee-Moultonborough-SEINE | Lake Winnipesaukee | Current presence | 2009 | NH | 308057 | 4844281 | GPS survey coordinates | 2 |
| 20090604-1230-BS-Lake Winnipesaukee-Moultonborough-SEINE | Lake Winnipesaukee | Current presence | 2009 | NH | 307531 | 4843826 | Mean center point of 2+ surveys at this location | 2 |
| 20090604-1300-BS-Lake Winnipesaukee-Moultonborough-SEINE | Lake Winnipesaukee | Current presence | 2009 | NH | 307596 | 4844580 | Mean center point of 2+ surveys at this location | 2 |
| 20090914-1200-WARMWATER-Lake Winnipesaukee-Moultonborough-EBOAT | Lake Winnipesaukee | Current presence | 2009 | NH | 307780 | 4845044 | Mean center point of 2+ surveys at this location | 2 |
| 20100610-1100-BS-Winnipesaukee Lake-Moultonborough-DIPNET | Lake Winnipesaukee | Current presence | 2010 | NH | 305611 | 4843959 | GPS survey coordinates | 2 |
| 20100615-1000-BS-Winnipesaukee Lake-Moultonborough-DIPNET | Lake Winnipesaukee | Current presence | 2010 | NH | 305746 | 4843979 | Mean center point of 2+ surveys at this location | 2 |
| 20100615-1100-BS-Winnipesaukee Lake-Moultonborough-DIPNET | Lake Winnipesaukee | Current presence | 2010 | NH | 306116 | 4844122 | Mean center point of 2+ surveys at this location | 2 |
| 20100728-1015-BS-Winnipesaukee Lake-Moultonborough-DIPNET | Lake Winnipesaukee | Current presence | 2010 | NH | 306623 | 4844466 | Mean center point of 2+ surveys at this location | 2 |
| 20100728-1100-BS-Winnipesaukee Lake-Moultonborough-DIPNET | Lake Winnipesaukee | Current presence | 2010 | NH | 307118 | 4844509 | GPS survey coordinates | 2 |
| 20100728-1230-BS-Winnipesaukee Lake-Moultonborough-DIPNET | Lake Winnipesaukee | Current presence | 2010 | NH | 307866 | 4844686 | GPS survey coordinates | 2 |
| 20100728-930-BS-Winnipesaukee Lake-Moultonborough-DIPNET | Lake Winnipesaukee | Current presence | 2010 | NH | 306331 | 4844282 | Mean center point of 2+ surveys at this location | 2 |
| 20100728-945-BS-Winnipesaukee Lake-Moultonborough-DIPNET | Lake Winnipesaukee | Current presence | 2010 | NH | 306569 | 4844282 | Mean center point of 2+ surveys at this location | 2 |
| 20100729-1030-BS-Winnipesaukee Lake-Moultonborough-DIPNET | Lake Winnipesaukee | Current presence | 2010 | NH | 310247 | 4838356 | GPS survey coordinates | 2 |
| 20100729-1130-BS-Winnipesaukee Lake-Moultonborough-DIPNET | Lake Winnipesaukee | Current presence | 2010 | NH | 307474 | 4845060 | GPS survey coordinates | 2 |
| 20120806-1100-BS-Winnipesaukee Lake-Moultonborough-DIPNET | Lake Winnipesaukee | Current presence | 2012 | NH | 303216 | 4842552 | Mean center point of 2+ surveys at this location | 2 |
| 20130509-1100-BS-Winnipesaukee Lake-Moultonborough-DIPNET | Lake Winnipesaukee | Current presence | 2013 | NH | 307548 | 4844394 | GPS survey coordinates | 2 |
| 20190702-1000-BS-Winnipesaukee Lake-Moultonborough-DIPNET | Lake Winnipesaukee | Current presence | 2019 | NH | 306859 | 4844601 | GPS survey coordinates | 2 |
| 20190702-1200-BS-Winnipesaukee Lake-Moultonborough-DIPNET | Lake Winnipesaukee | Current presence | 2019 | NH | 305832 | 4844014 | GPS survey coordinates | 2 |
| 20100914-1000-BS-Lamprey River-Raymond-DIPNET | Lamprey River | Current presence | 2010 | NH | 319671 | 4768920 | GPS survey coordinates | 2 |
| 20100914-1200-BS-Lamprey River-Raymond-DIPNET | Lamprey River | Current presence | 2010 | NH | 318991 | 4769404 | GPS survey coordinates | 2 |
| 20110722-1100-BS-Lamprey River-Epping-SEINE | Lamprey River | Current presence | 2011 | NH | 326474 | 4767364 | Mean center point of 2+ surveys at this location | 2 |
| 20110811-930-BS-Lamprey River-Raymond-DIPNET | Lamprey River | Current presence | 2011 | NH | 324722 | 4765503 | GPS survey coordinates | 2 |
| 20110823-1100-BS-Lamprey River-Raymond-DIPNET | Lamprey River | Current presence | 2011 | NH | 321419 | 4767076 | GPS survey coordinates | 2 |
| 20120817-800-BS-Lamprey River-Epping-DIPNET | Lamprey River | Current presence | 2012 | NH | 326779 | 4767701 | GPS survey coordinates | 2 |
| 20180629-930-BS-Lamprey River-Epping-DIPNET | Lamprey River | Current presence | 2018 | NH | 326688 | 4767643 | GPS survey coordinates | 2 |
| 20100618-1030-BS-Lees Pond-Moultonborough-DIPNET | Lees Pond | Current presence | 2010 | NH | 306392 | 4845825 | GPS survey coordinates | 2 |
| 20210712-1200-BS-Moores Pond-Tamworth-DIPNET | Moores Pond | Current presence | 2021 | NH | 323202 | 4858492 | GPS survey coordinates | 2 |
| MUDNO | Mud Pond | Current presence | 2022 | ME | 358926 | 4865646 | Mean center point of eDNA sample replicates collected at this location | 13 |
| 20140723-1000-BS-Northeast Pond-Milton-DIPNET | Northeast Pond | Current presence | 2014 | NH | 342338 | 4810897 | GPS survey coordinates | 2 |
| 20140723-800-BS-Northeast Pond-Milton-DIPNET | Northeast Pond | Current presence | 2014 | NH | 341558 | 4814410 | GPS survey coordinates | 2 |
| 20190809-930-BS-Northeast Pond-Milton-DIPNET | Northeast Pond | Current presence | 2019 | NH | 341348 | 4815046 | GPS survey coordinates | 2 |
| OSSIPE | Ossipee River | Current presence | 2021 | ME | 353203 | 4852143 | Mean center point of seine and/or eDNA sample replicates collected at this location | 13 |
| OSSIPM | Ossipee River | Current presence | 2021 | ME | 343737 | 4850670 | Mean center point of seine and/or eDNA sample replicates collected at this location | 13 |
| OSSIPM | Ossipee River | Current presence | 2021 | ME | 343794 | 4850679 | Mean center point of seine and/or eDNA sample replicates collected at this location | 13 |
| PANTHR-01 | Panther Pond | Current presence | 2022 | ME | 382047 | 4866257 | Mean center point of eDNA sample replicates collected at this location | 13 |
| 20190724-1000-BS-Pemigewasset Lake-New Hampton-DIPNET | Pemingewasset Lake | Current presence | 2019 | NH | 289892 | 4832801 | Mean center point of 2+ surveys at this location | 2 |
| 20150723-1030-BS-Powwow River-South Hampton-DIPNET | Powwow River | Current presence | 2015 | NH | 337701 | 4749517 | GPS survey coordinates | 2 |
| PRESUM-01 | Presumpscot River | Current presence | 2021 | ME | 383527 | 4845097 | Mean center point of seine and/or eDNA sample replicates collected at this location | 13 |
| PRESUM-02 | Presumpscot River | Current presence | 2021 | ME | 383596 | 4845196 | Mean center point of seine and/or eDNA sample replicates collected at this location | 13 |
| 20210618-1030-BS-Purity Lake-Eaton-DIPNET | Purity Lake | Current presence | 2021 | NH | 332664 | 4860539 | GPS survey coordinates | 2 |
| 20170926-1030-BS-Red Hill River-Sandwich-DIPNET | Red Hill River | Current presence | 2017 | NH | 303589 | 4851424 | GPS survey coordinates | 2 |
| 20170926-900-BS-Red Hill River-Sandwich-DIPNET | Red Hill River | Current presence | 2017 | NH | 303311 | 4851842 | GPS survey coordinates | 2 |
| 20220711-1030-BS-Red Hill River-Moultonborough-DIPNET | Red Hill River | Current presence | 2022 | NH | 305584 | 4849758 | GPS survey coordinates | 2 |
| 20220711-1130-BS-Red Hill River-Moultonborough-DIPNET | Red Hill River | Current presence | 2022 | NH | 305648 | 4849550 | GPS survey coordinates | 2 |
| 20140909-1300-BS-Ryefield Brook-Wolfeboro-DIPNET | Ryefield Brook | Current presence | 2014 | NH | 327387 | 4830765 | GPS survey coordinates | 2 |
| SACONO-03 | Saco River | Current presence | 2021 | ME | 353523 | 4862366 | Mean center point of seine and/or eDNA sample replicates collected at this location | 13 |
| 20190809-1000-BS-Salmon Falls River-Milton-DIPNET | Salmon Falls River | Current presence | 2019 | NH | 340682 | 4815322 | GPS survey coordinates | 2 |
| 20190809-1100-BS-Salmon Falls River-Milton-DIPNET | Salmon Falls River | Current presence | 2019 | NH | 340588 | 4815327 | GPS survey coordinates | 2 |
| 20190809-1230-BS-Salmon Falls River-Milton-DIPNET | Salmon Falls River | Current presence | 2019 | NH | 340628 | 4815973 | GPS survey coordinates | 2 |
| 20190809-945-BS-Salmon Falls River-Milton-DIPNET | Salmon Falls River | Current presence | 2019 | NH | 341112 | 4815264 | GPS survey coordinates | 2 |
| 20200818-1030-BS-Salmon Falls River-Milton-DIPNET | Salmon Falls River | Current presence | 2020 | NH | 341080 | 4817411 | GPS survey coordinates | 2 |
| SFALLS | Salmon Falls River | Current presence | 2021 | ME | 345383 | 4796431 | Mean center point of seine and/or eDNA sample replicates collected at this location | 13 |
| 20130603-1200-EBTJV-Seaver Brook-Plaistow-EFISH | Seaver Brook | Current presence | 2013 | NH | 329402 | 4743961 | GPS survey coordinates | 2 |
| SEBAGO-01 | Sebago Lake | Current presence | 2021 | ME | 373507 | 4863505 | Mean center point of seine and/or eDNA sample replicates collected at this location | 13 |
| 20190920-900-BS-Soucook River-Loudon-DIPNET | Soucook River | Current presence | 2019 | NH | 299964 | 4795891 | GPS survey coordinates | 2 |
| 20210616-1000-BS-Soucook River-Loudon-DIPNET | Soucook River | Current presence | 2021 | NH | 301116 | 4807381 | GPS survey coordinates | 2 |
| 20190920-1100-BS-Suncook River-Barnstead-DIPNET | Suncook River | Current presence | 2019 | NH | 314287 | 4807806 | GPS survey coordinates | 2 |
| CRESLK-01 | Tenny River | Current presence | 2022 | ME | 382590 | 4867344 | Mean center point of eDNA sample replicates collected at this location | 13 |
| PANTHR-01 | Tenny River | Current presence | 2022 | ME | 382071 | 4866266 | Mean center point of eDNA sample replicates collected at this location | 13 |
| 20150924-1015-BS-unnamed pond-Moultonborough-DIPNET | Unnamed pond | Current presence | 2015 | NH | 299997 | 4847941 | GPS survey coordinates | 2 |
| 20151008-1230-BS-Heron Pond-Moultonborough-DIPNET | Unnamed pond | Current presence | 2015 | NH | 300604 | 4845925 | GPS survey coordinates | 2 |
| 20220628-1100-BS-Warren Hatchery Pond-Warren-DIPNET | Unnamed pond | Current presence | 2022 | NH | 268205 | 4866061 | Mean center point of 2+ surveys at this location | 2 |
| 20210715-1000-BS-Upper Suncook Lake-Barnstead-DIPNET | Upper Suncook Lake | Current presence | 2021 | NH | 314625 | 4807431 | GPS survey coordinates | 2 |
| 20140909-1000-BS-Warren Brook-Wolfeboro-DIPNET | Warren Brook | Current presence | 2014 | NH | 328940 | 4828228 | GPS survey coordinates | 2 |
| ANDROS | Androscoggin River | Current absence | 2022 | ME | 405089 | 4877012 | Mean center point of eDNA sample replicates collected at this location | 13 |
| 20200813-1300-BS-Archers Pond-Ossipee-DIPNET | Archers Pond | Current absence | 2020 | NH | 327486 | 4843183 | GPS survey coordinates | 2 |
| BARKER | Barker Pond | Current absence | 2021 | ME | 358996 | 4860972 | Mean center point of seine and/or eDNA sample replicates collected at this location | 13 |
| GWORKB | Bauneg Beg Pond | Current absence | 2021 | ME | 359003 | 4801935 | Mean center point of seine and/or eDNA sample replicates collected at this location | 13 |
| BEARPD-01 | Bear Pond | Current absence | 2021 | ME | 362600 | 4890828 | Mean center point of seine and/or eDNA sample replicates collected at this location | 13 |
| BEARPD-02 | Bear Pond | Current absence | 2021 | ME | 363311 | 4889371 | Mean center point of seine and/or eDNA sample replicates collected at this location | 13 |
| 20210723-1000-BS-Bearcamp Pond-Sandwich-DIPNET | Bearcamp Pond | Current absence | 2021 | NH | 308843 | 4854626 | GPS survey coordinates | 2 |
| 20190924-1400-BS-Beaver Brook-New Durham-DIPNET | Beaver Brook | Current absence | 2019 | NH | 325161 | 4822405 | GPS survey coordinates | 2 |
| 20160922-900-BS-Berry Pond-Moultonborough-DIPNET | Berry Pond | Current absence | 2016 | NH | 307391 | 4847846 | GPS survey coordinates | 2 |
| BRADPD-01 | Bradley Pond | Current absence | 2022 | ME | 351426 | 4899886 | Mean center point of eDNA sample replicates collected at this location | 13 |
| BRADPD-02 | Bradley Pond | Current absence | 2022 | ME | 351075 | 4899369 | Mean center point of eDNA sample replicates collected at this location | 13 |
| 20210715-1200-BS-Brindle Pond-Barnstead-DIPNET | Brindle Pond | Current absence | 2021 | NH | 318108 | 4803749 | GPS survey coordinates | 2 |
| BROBRK | Browns Brook | Current absence | 2021 | ME | 554519 | 4967959 | Mean center point of seine and/or eDNA sample replicates collected at this location | 13 |
| BUFFBR | Buff Brook | Current absence | 2022 | ME | 356584 | 4828560 | Mean center point of eDNA sample replicates collected at this location | 13 |
| BURNPD-01 | Burnt Meadow Pond | Current absence | 2021 | ME | 348499 | 4865679 | Mean center point of seine and/or eDNA sample replicates collected at this location | 13 |
| BURNPD-02 | Burnt Meadow Pond | Current absence | 2021 | ME | 348622 | 4865064 | Mean center point of seine and/or eDNA sample replicates collected at this location | 13 |
| UNK-Canobie Lake-Salem/Windham | Canobie Lake | Current absence | Unknown | NH | 315568 | 4740615 | Center of waterbody: no precise location known | 5 |
| CARBRK | Carsley Brook | Current absence | 2022 | ME | 368261 | 4882325 | Mean center point of eDNA sample replicates collected at this location | 13 |
| CARBRK | Carsley Brook | Current absence | 2022 | ME | 368272 | 4882347 | Mean center point of eDNA sample replicates collected at this location | 13 |
| 20210802-940-BS-Cawley Pond-Sanbornton-DIPNET | Cawley Pond | Current absence | 2021 | NH | 289508 | 4824108 | GPS survey coordinates | 2 |
| CHANBR | Chandler Brook | Current absence | 2022 | ME | 401966 | 4862414 | Mean center point of eDNA sample replicates collected at this location | 13 |
| 20210712-1000-BS-Chocorua Lake-Tamworth-DIPNET | Chocorua Lake | Current absence | 2021 | NH | 320804 | 4862778 | GPS survey coordinates | 2 |
| COLCPD-01 | Colcord Pond | Current absence | 2022 | ME | 342809 | 4855549 | Mean center point of eDNA sample replicates collected at this location | 13 |
| COLCPD-02 | Colcord Pond | Current absence | 2022 | ME | 342637 | 4857682 | Mean center point of eDNA sample replicates collected at this location | 13 |
| 20210716-1100-BS-Conway Lake-Conway-DIPNET | Conway Lake | Current absence | 2021 | NH | 335622 | 4871631 | GPS survey coordinates | 2 |
| 20160830-930-BS-Copp Brook-Wakefield-DIPNET | Copp Brook | Current absence | 2016 | NH | 338388 | 4826564 | GPS survey coordinates | 2 |
| 20160922-1130-BS-Copps Pond-Tuftonboro-DIPNET | Copps Pond | Current absence | 2016 | NH | 316279 | 4838673 | GPS survey coordinates | 2 |
| CRESLK-01 | Crescent Lake | Current absence | 2022 | ME | 382630 | 4867352 | Mean center point of eDNA sample replicates collected at this location | 13 |
| CRESLK-03 | Crescent Lake | Current absence | 2022 | ME | 382680 | 4871890 | Mean center point of eDNA sample replicates collected at this location | 13 |
| CROOKN | Crooked River | Current absence | 2022 | ME | 357337 | 4900567 | Mean center point of eDNA sample replicates collected at this location | 13 |
| CROOKR | Crooked River | Current absence | 2021 | ME | 373954 | 4873047 | Mean center point of seine and/or eDNA sample replicates collected at this location | 13 |
| CROOKR | Crooked River | Current absence | 2021 | ME | 373935 | 4873110 | Mean center point of seine and/or eDNA sample replicates collected at this location | 13 |
| CROOKS | Crooked River | Current absence | 2022 | ME | 374470 | 4870827 | Mean center point of eDNA sample replicates collected at this location | 13 |
| DINGLY | Dingley Brook | Current absence | 2022 | ME | 378809 | 4863133 | Mean center point of eDNA sample replicates collected at this location | 13 |
| DUCKIN | Duck Pond Brook | Current absence | 2022 | ME | 358278 | 4884976 | Mean center point of eDNA sample replicates collected at this location | 13 |
| DUCKNO | Duck Pond Brook | Current absence | 2022 | ME | 357414 | 4889865 | Mean center point of eDNA sample replicates collected at this location | 13 |
| EDDYBR | Eddy Brook | Current absence | 2022 | ME | 393682 | 4868007 | Mean center point of eDNA sample replicates collected at this location | 13 |
| GRTBRK | Great Brook | Current absence | 2022 | ME | 346435 | 4846972 | Mean center point of eDNA sample replicates collected at this location | 13 |
| GWORKN | Great Works River | Current absence | 2021 | ME | 357525 | 4805673 | Mean center point of seine and/or eDNA sample replicates collected at this location | 13 |
| GWORKS | Great Works River | Current absence | 2021 | ME | 359010 | 4797536 | Mean center point of seine and/or eDNA sample replicates collected at this location | 13 |
| OTTER-02 | Half Moon Pond | Current absence | 2022 | ME | 378313 | 4846655 | Mean center point of eDNA sample replicates collected at this location | 13 |
| 20170929-930-BS-Harper Brook -New Hampton-DIPNET | Harper Brook | Current absence | 2017 | NH | 287105 | 4833896 | GPS survey coordinates | 2 |
| 20210722-1200-BS-Hawkins Pond-Center Harbor-DIPNET | Hawkins Pond | Current absence | 2021 | NH | 293836 | 4840324 | GPS survey coordinates | 2 |
| 20200708-900-BS-Heads Pond-Hooksett-DIPNET | Heads Pond | Current absence | 2020 | NH | 301469 | 4774897 | GPS survey coordinates | 2 |
| 20210802-1330-BS-Hermit Lake-Sanbornton-DIPNET | Hermit Lake | Current absence | 2021 | NH | 289184 | 4827445 | GPS survey coordinates | 2 |
| HIGHLK-02 | Highland Lake | Current absence | 2021 | ME | 358456 | 4884496 | Mean center point of seine and/or eDNA sample replicates collected at this location | 13 |
| HIGHLK-03 | Highland Lake | Current absence | 2021 | ME | 362824 | 4879478 | Mean center point of seine and/or eDNA sample replicates collected at this location | 13 |
| 20210714-1030-BS-Horn Pond-Wakefield-DIPNET | Horn Pond | Current absence | 2021 | NH | 341178 | 4825926 | GPS survey coordinates | 2 |
| INGALS-01 | Ingalls Pond | Current absence | 2022 | ME | 355971 | 4858146 | Mean center point of eDNA sample replicates collected at this location | 13 |
| INGALS-02 | Ingalls Pond | Current absence | 2022 | ME | 356010 | 4857860 | Mean center point of eDNA sample replicates collected at this location | 13 |
| 20200707-1230-BS-Isinglass River-Barrington-DIPNET | Isinglass River | Current absence | 2020 | NH | 330006 | 4790143 | GPS survey coordinates | 2 |
| JOSIES | Josies Brook | Current absence | 2021 | ME | 369752 | 4840508 | Mean center point of seine and/or eDNA sample replicates collected at this location | 13 |
| JOSIES | Josies Brook | Current absence | 2021 | ME | 369713 | 4840554 | Mean center point of seine and/or eDNA sample replicates collected at this location | 13 |
| JOSIES | Josies Brook | Current absence | 2021 | ME | 369673 | 4840574 | Mean center point of seine and/or eDNA sample replicates collected at this location | 13 |
| UNK-Winnipesaukee Lake-Fish Cove-Meredith | Lake Winnipesaukee | Current absence | Unknown | NH | 303342 | 4835248 | Digitized coordinates: northern end of cove | 5 |
| UNK-Winnisquam Lake-Sanbornton/Laconia/Belmont | Lake Winnisquam | Current absence | Unknown | NH | 297443 | 4823795 | Center of waterbody: no precise location known | 5 |
| 20220914-1330-BS-Lamprey River-Newmarket-DIPNET | Lamprey River | Current absence | 2022 | NH | 341924 | 4772629 | GPS survey coordinates | 2 |
| UNK-Lamprey River-Bunker Pond-Epping | Lamprey River | Current absence | Unknown | NH | 326528 | 4767262 | Digitized coordinates from 1992 aerial imagery (Google Earth) | 5 |
| LITTLR | Little River | Current absence | 2021 | ME | 350404 | 4803866 | Mean center point of seine and/or eDNA sample replicates collected at this location | 13 |
| LITTLR | Little River | Current absence | 2021 | ME | 350508 | 4803767 | Mean center point of seine and/or eDNA sample replicates collected at this location | 13 |
| 20220826-1030-BS-Long Pond-Northwood-DIPNET | Long Pond | Current absence | 2022 | NH | 319112 | 4790395 | GPS survey coordinates | 2 |
| UNK-Lower Suncook Lake-Barnstead | Lower Suncook Lake | Current absence | Unknown | NH | 316474 | 4804418 | Center of waterbody: no precise location known | 5 |
| MARRPD-01 | Marr Pond | Current absence | 2022 | ME | 476038 | 4999615 | Mean center point of eDNA sample replicates collected at this location | 13 |
| MARRPD-02 | Marr Pond | Current absence | 2022 | ME | 476736 | 4999434 | Mean center point of eDNA sample replicates collected at this location | 13 |
| MARBRK | Marshall Brook | Current absence | 2021 | ME | 551549 | 4902260 | Mean center point of seine and/or eDNA sample replicates collected at this location | 13 |
| MARBRK | Marshall Brook | Current absence | 2021 | ME | 551462 | 4902331 | Mean center point of seine and/or eDNA sample replicates collected at this location | 13 |
| MEADBR | Meadow Brook | Current absence | 2022 | ME | 413726 | 4869189 | Mean center point of eDNA sample replicates collected at this location | 13 |
| MEADBR | Meadow Brook | Current absence | 2022 | ME | 413707 | 4869164 | Mean center point of eDNA sample replicates collected at this location | 13 |
| MERRIL | Merrill Brook | Current absence | 2022 | ME | 408712 | 4855826 | Mean center point of eDNA sample replicates collected at this location | 13 |
| MERRIL | Merrill Brook | Current absence | 2022 | ME | 408757 | 4855859 | Mean center point of eDNA sample replicates collected at this location | 13 |
| RANGE-01 | Middle Range Pond | Current absence | 2022 | ME | 389396 | 4876975 | Mean center point of eDNA sample replicates collected at this location | 13 |
| RANGE-02 | Middle Range Pond | Current absence | 2022 | ME | 388940 | 4874366 | Mean center point of eDNA sample replicates collected at this location | 13 |
| MOSQPD | Mosquito Pond | Current absence | 2022 | ME | 356553 | 4906749 | Mean center point of eDNA sample replicates collected at this location | 13 |
| MOSQPD | Mosquito Pond | Current absence | 2022 | ME | 356591 | 4906800 | Mean center point of eDNA sample replicates collected at this location | 13 |
| MUDNO | Mud Pond | Current absence | 2022 | ME | 358925 | 4865722 | Mean center point of eDNA sample replicates collected at this location | 13 |
| MUDNO | Mud Pond | Current absence | 2022 | ME | 358894 | 4865593 | Mean center point of eDNA sample replicates collected at this location | 13 |
| MUDSO | Mud Pond | Current absence | 2022 | ME | 348493 | 4830536 | Mean center point of eDNA sample replicates collected at this location | 13 |
| 20200805-900-BS-Nippo Brook-Barrington-DIPNET | Nippo Brook | Current absence | 2020 | NH | 330050 | 4788388 | GPS survey coordinates | 2 |
| OCSACO | Old Course Saco River | Current absence | 2021 | ME | 346249 | 4884452 | Mean center point of seine and/or eDNA sample replicates collected at this location | 13 |
| OSSIPR | Ossipee River | Current absence | 2022 | ME | 353409 | 4852082 | Mean center point of eDNA sample replicates collected at this location | 13 |
| UNK-Oyster River-Mill Pond-Durham | Oyster River | Current absence | Unknown | NH | 343760 | 4777090 | Point in approximate center of impoundment: exact location unknown | 5 |
| PANTHR-02 | Panther Pond | Current absence | 2022 | ME | 381933 | 4863544 | Mean center point of eDNA sample replicates collected at this location | 13 |
| PANTHR-03 | Panther Pond | Current absence | 2022 | ME | 383458 | 4864876 | Mean center point of eDNA sample replicates collected at this location | 13 |
| JORDAN | Panther Run | Current absence | 2021 | ME | 382391 | 4860701 | Mean center point of seine and/or eDNA sample replicates collected at this location | 13 |
| JORDAN | Panther Run | Current absence | 2021 | ME | 382451 | 4860670 | Mean center point of seine and/or eDNA sample replicates collected at this location | 13 |
| 20200729-930-BS-Pine River-Effingham-DIPNET | Pine River | Current absence | 2020 | NH | 329392 | 4846184 | GPS survey coordinates | 2 |
| PISCAT | Piscataqua River | Current absence | 2021 | ME | 394379 | 4847547 | Mean center point of seine and/or eDNA sample replicates collected at this location | 13 |
| PISCDN | Piscataqua River | Current absence | 2022 | ME | 395385 | 4845115 | Mean center point of eDNA sample replicates collected at this location | 13 |
| PISCUP | Piscataqua River | Current absence | 2022 | ME | 394821 | 4850479 | Mean center point of eDNA sample replicates collected at this location | 13 |
| PISCUP | Piscataqua River | Current absence | 2022 | ME | 394877 | 4850524 | Mean center point of eDNA sample replicates collected at this location | 13 |
| UNK-Pleasant Lake-Deerfield | Pleasant Lake | Current absence | Unknown | NH | 316353 | 4784453 | Center of waterbody: no precise location known | 5 |
| PRESBG | Presumpscot River | Current absence | 2022 | ME | 383497 | 4846935 | Mean center point of eDNA sample replicates collected at this location | 13 |
| PROCPD-01 | Proctor Pond | Current absence | 2021 | ME | 356417 | 4900505 | Mean center point of seine and/or eDNA sample replicates collected at this location | 13 |
| PROCPD-02 | Proctor Pond | Current absence | 2021 | ME | 356622 | 4900277 | Mean center point of seine and/or eDNA sample replicates collected at this location | 13 |
| 20200813-1100-BS-Purity Lake-Madison-DIPNET | Purity Lake | Current absence | 2020 | NH | 332157 | 4858595 | GPS survey coordinates | 2 |
| REDBRK | Red Brook | Current absence | 2022 | ME | 391700 | 4831261 | Mean center point of eDNA sample replicates collected at this location | 13 |
| 20210623-930-BS-Rocky Pond-Gilmanton-DIPNET | Rocky Pond | Current absence | 2021 | NH | 301241 | 4808763 | GPS survey coordinates | 2 |
| 20210802-1230-BS-Rollins Pond-Sanbornton-DIPNET | Rollins Pond | Current absence | 2021 | NH | 289596 | 4823155 | GPS survey coordinates | 2 |
| ROYAL | Royal River | Current absence | 2022 | ME | 398033 | 4874305 | Mean center point of eDNA sample replicates collected at this location | 13 |
| ROYAL | Royal River | Current absence | 2022 | ME | 398085 | 4874309 | Mean center point of eDNA sample replicates collected at this location | 13 |
| SACONO-01 | Saco River | Current absence | 2021 | ME | 353461 | 4862049 | Mean center point of seine and/or eDNA sample replicates collected at this location | 13 |
| SACONO-02 | Saco River | Current absence | 2021 | ME | 353667 | 4862320 | Mean center point of seine and/or eDNA sample replicates collected at this location | 13 |
| SACOSO | Saco River | Current absence | 2021 | ME | 356709 | 4851980 | Mean center point of seine and/or eDNA sample replicates collected at this location | 13 |
| 20170814-1030-BS-Salmon Falls River-Milton-DIPNET | Salmon Falls River | Current absence | 2017 | NH | 342012 | 4819286 | GPS survey coordinates | 2 |
| SEBAGO-03 | Sebago Lake | Current absence | 2021 | ME | 370319 | 4864974 | Mean center point of seine and/or eDNA sample replicates collected at this location | 13 |
| SEBAGO-06 | Sebago Lake | Current absence | 2021 | ME | 381748 | 4861726 | Mean center point of seine and/or eDNA sample replicates collected at this location | 13 |
| UNK-Shadow Lake-Salem/Windham | Shadow Lake | Current absence | Unknown | NH | 316939 | 4743138 | Center of waterbody: no precise location known | 5 |
| SHEPR | Shepards River | Current absence | 2022 | ME | 345342 | 4866439 | Mean center point of eDNA sample replicates collected at this location | 13 |
| OTTER-01 | Snake Pond | Current absence | 2022 | ME | 378903 | 4846581 | Mean center point of eDNA sample replicates collected at this location | 13 |
| SOKOLK-01 | Sokokis Lake | Current absence | 2022 | ME | 356131 | 4839975 | Mean center point of eDNA sample replicates collected at this location | 13 |
| SOKOLK-02 | Sokokis Lake | Current absence | 2022 | ME | 354658 | 4841389 | Mean center point of eDNA sample replicates collected at this location | 13 |
| SEBAGO-01 | Songo River | Current absence | 2021 | ME | 373573 | 4863697 | Mean center point of seine and/or eDNA sample replicates collected at this location | 13 |
| SOPER | Soper Mill Brook | Current absence | 2022 | ME | 402200 | 4875477 | Mean center point of eDNA sample replicates collected at this location | 13 |
| 20160830-1230-BS-South River-Effingham-DIPNET | South River | Current absence | 2016 | NH | 338958 | 4840538 | GPS survey coordinates | 2 |
| SPECPD | Spectacle Pond | Current absence | 2021 | ME | 346912 | 4853557 | Mean center point of seine and/or eDNA sample replicates collected at this location | 13 |
| SPECPD | Spectacle Pond | Current absence | 2021 | ME | 347075 | 4853646 | Mean center point of seine and/or eDNA sample replicates collected at this location | 13 |
| RACHEL | Spurwink River | Current absence | 2022 | ME | 396686 | 4827272 | Mean center point of eDNA sample replicates collected at this location | 13 |
| 20200812-1000-BS-Squam River-Ashland-DIPNET | Squam River | Current absence | 2020 | NH | 288770 | 4843687 | GPS survey coordinates | 2 |
| STANPD-01 | Stanley Pond | Current absence | 2021 | ME | 348513 | 4854698 | Mean center point of seine and/or eDNA sample replicates collected at this location | 13 |
| STANPD-02 | Stanley Pond | Current absence | 2021 | ME | 348085 | 4854932 | Mean center point of seine and/or eDNA sample replicates collected at this location | 13 |
| STANPD-03 | Stanley Pond | Current absence | 2021 | ME | 347761 | 4855828 | Mean center point of seine and/or eDNA sample replicates collected at this location | 13 |
| SEBAGO-04 | Sticky River | Current absence | 2021 | ME | 375388 | 4848545 | Mean center point of seine and/or eDNA sample replicates collected at this location | 13 |
| SEBAGO-04 | Sticky River | Current absence | 2021 | ME | 375337 | 4848562 | Mean center point of seine and/or eDNA sample replicates collected at this location | 13 |
| 20170913-1100-BS-Suncook River-Epsom-DIPNET | Suncook River | Current absence | 2017 | NH | 308338 | 4790514 | GPS survey coordinates | 2 |
| SYMMES-01 | Symmes Pond | Current absence | 2022 | ME | 348891 | 4834448 | Mean center point of eDNA sample replicates collected at this location | 13 |
| HEATH-01 | The Heath | Current absence | 2022 | ME | 382072 | 4875178 | Mean center point of eDNA sample replicates collected at this location | 13 |
| HEATH-02 | The Heath | Current absence | 2022 | ME | 381928 | 4874663 | Mean center point of eDNA sample replicates collected at this location | 13 |
| TRAFPD-01 | Trafton Pond | Current absence | 2021 | ME | 348269 | 4856242 | Mean center point of seine and/or eDNA sample replicates collected at this location | 13 |
| TRAFPD-02 | Trafton Pond | Current absence | 2021 | ME | 347795 | 4856809 | Mean center point of seine and/or eDNA sample replicates collected at this location | 13 |
| 20170814-1230-BS-Union Meadows Pond-Wakefield-DIPNET | Union Meadows Pond | Current absence | 2017 | NH | 336298 | 4818783 | GPS survey coordinates | 2 |
| BOOMBR | Unnamed brook | Current absence | 2021 | ME | 377162 | 4819899 | Mean center point of seine and/or eDNA sample replicates collected at this location | 13 |
| BOOMBR | Unnamed brook | Current absence | 2021 | ME | 377133 | 4819866 | Mean center point of seine and/or eDNA sample replicates collected at this location | 13 |
| CANCO | Unnamed brook | Current absence | 2022 | ME | 396704 | 4837552 | Mean center point of eDNA sample replicates collected at this location | 13 |
| RANGE-01 | Unnamed brook | Current absence | 2022 | ME | 389394 | 4877006 | Mean center point of eDNA sample replicates collected at this location | 13 |
| RIDGEB | Unnamed brook | Current absence | 2022 | ME | 363811 | 4826613 | Mean center point of eDNA sample replicates collected at this location | 13 |
| WATBRK | Unnamed brook | Current absence | 2021 | ME | 368059 | 4845438 | Mean center point of seine and/or eDNA sample replicates collected at this location | 13 |
| WATBRK | Unnamed brook | Current absence | 2021 | ME | 368114 | 4845449 | Mean center point of seine and/or eDNA sample replicates collected at this location | 13 |
| 20170929-1430-BS-unnamed pond-Meredith-DIPNET | Unnamed pond | Current absence | 2017 | NH | 292235 | 4832668 | GPS survey coordinates | 2 |
| 20210723-1200-BS-Berry Pond-Moultonborough-DIPNET | Weed Brook | Current absence | 2021 | NH | 307193 | 4848852 | GPS survey coordinates | 2 |
| UNK-Wheelwright Pond-Lee | Wheelwright Pond | Current absence | Unknown | NH | 336471 | 4778033 | Center of waterbody: no precise location known | 5 |
| 20210716-1400-BS-White Lake-Tamworth-DIPNET | White Lake | Current absence | 2021 | NH | 321375 | 4855971 | GPS survey coordinates | 2 |
| 20210715-1400-BS-Whites Pond-Pittsfield-DIPNET | Whites Pond | Current absence | 2021 | NH | 312329 | 4797021 | GPS survey coordinates | 2 |
| 20210722-1000-BS-Winona Lake-New Hampton-DIPNET | Winona Lake | Current absence | 2021 | NH | 293446 | 4838540 | GPS survey coordinates | 2 |
| **Sources**: (1) New Hampshire Fish and Game Department (NHFGD) n.d.; (2) M. Carpenter, NHFGD, written communication, 7 February 2023; (3) Cooper 1939; (4) M. Gallagher, Maine Department of Inland Fisheries and Wildlife (MDIFW), written communication, 25 January 2021; (5) NHFGD 2015; (6) Kendall 1914; (7) North American Native Fishes Association 2023; (8) Doering et al. 1995; (9) Harrington 1947b; (10) USEPA 2016, (11) Harrington 1948; (12) Vaux 2013; (13) University of Maine bridle shiner surveys 2021-2022 (this study). | | | | | | | | |

## **REFERENCES**

Cooper, G. P. 1939. A biological survey of thirty-one lakes and ponds of the Upper Saco River and Sebago Lake drainage systems in Maine. Maine Department of Inland Fisheries and Game, Fish Survey Report 2. Available from https://digitalmaine.com/ifw_docs/70/ [accessed March 2021].

Doering, P. H., C. T. Roman, L. L. Beatty, A. A. Keller, and C. A. Oviatt. 1995. Water quality and habitat evaluation of Bass Harbor Marsh Acadia National Park, Maine. Page 213. National Park Service, New England System Support Office (NESO), NPS/NESORNR/NRTR/95-31, Boston, MA. Available from https://openparksnetwork.org/single-item-view/?oid=OPN_NS:E413FC0BFEFB4D4F437728EC961791BC#page/1/mode/2up [accessed February 2021].

Harrington, R. W. 1947b. The early life history of the bridled shiner, *Notropis bifrenatus* (Cope). Copeia 1947(2):97–102.

Harrington, R. W. 1948a. The life cycle and fertility of the bridled shiner, *Notropis bifrenatus* (Cope). The American Midland Naturalist 39(1):83–92.

Kendall, W.C. 1914. An annotated catalogue of the fishes of Maine. Portland Society of Natural History, Portland, ME. Available from https://www.biodiversitylibrary.org/item/45961.

New Hampshire Fish and Game Dept. (NHFGD). (n.d.). New Hampshire fish survey map. Available from https://nhfg.maps.arcgis.com/apps/MapJournal/index.html?appid=d6549e90155b441fa0e29bdc44eebc2b# [accessed 25 February 2025].

New Hampshire Fish and Game Dept. (NHFGD). 2015. New Hampshire Wildlife Action Plan. New Hampshire Fish and Game Department, SWG Report, Concord, NH. Available from https://www.wildlife.nh.gov/wildlife-and-habitat/nh-wildlife-action-plan/swap-2015 [accessed May 2023].

North American Native Fishes Association. 2023. Bridle shiner—*Notropis bifrenatus*. Available from https://fishmap.org/species/Bridle-Shiner.html [accessed January 2023].

U.S. Environmental Protection Agency (USEPA). 2016. Region 1 Maine lakes data sets. https://archive.epa.gov/emap/archive-emap/web/html/index-158.html.

Vaux, P. D. 2013. Maine Aquatic Biodiversity Project Database [Dataset]. Available from https://www.gulfofmaine.org/kb/2.0/record.html?recordid=9790 [accessed September 2023].

**Supplement E. HUC14 extraction using GRASS-GIS.**

U.S. Geological Survey (USGS) Hydrological Unit Code 14 (HUC14) polygons were not available for all of Maine and New Hampshire, so we used the USGS 3D Elevation Program 10-meter resolution Digital Elevation Model (USGS 1998) as a base digital elevation model (DEM) to extract HUC14s. We downloaded the DEM at a 30-m resolution from a geospatial analysis platform (Google Earth Engine, Google LLC, Mountain View, California, USA) as the 10-m resolution proved to be too computationally intensive for subsequent analyses. We used a geographic information system (GRASS-GIS, version 8.3; Neteler et al. 2012) run through package *rgrass* (version 0.4-2; Bivand 2024) in Program R (version 4.3.3; R Core Team 2024) to delineate HUC14s (code provided in Katz 2024). We determined the computational region of the delineation process by calculating the bounding box of all watersheds (HUC10 polygons) that intersected a 100-km buffer around the known historical range of bridle shiners in New Hampshire and Maine (i.e., the Saco and Merrimack HUC6 basins). We then dissolved the boundaries between the selected HUC10 polygons and rasterized them to create a mask raster.

We applied the GRASS-GIS function “*r.watershed*” with flags “*-s*” (D8 flow) and “*-b*” (beautify flat areas) and a threshold value (the minimum number of upstream grid cells needed to initiate a river and create HUC14s [Friedrichs‐Manthey et al. 2020]) of 14400 raster cells to delineate HUC14s. We determined the threshold value by first calculating the mean area of both HUC12s and HUC14s in Maine and New Hampshire (USGS 2021), then comparing this mean area to threshold values reported by Friedrichs‐Manthey et al. (2020). We determined that HUC12s correspond most closely to HUC14s calculated with a threshold value of 12800 cells, and that HUC14 HUC14s correspond most closely to HUC14s calculated with a threshold value between 1600 and 3200 cells. Because Friedrichs‐Manthey et al. (2020) used a 90-m DEM to calculate their HUC14s, we multiplied these threshold values by nine to determine an equivalent threshold using a 30-m DEM. We chose the lower of the two values (14400 cells) to delineate HUC14s at a finer scale. We then vectorized the HUC14s into polygons using the “*r.to.vect*” function with flag “*-s*” to smooth corners. We created additional HUC14s where the “*r.watershed*” function left gaps along the coastline, and then removed any overlapping areas (polygons < 0.029-km^2^) between HUC14s.

## **REFERENCES**

Bivand, R. S. 2024. *rgrass*: Interface between “GRASS” Geographical Information System and “R”. R.

Friedrichs‐Manthey, M., S. D. Langhans, T. Hein, F. Borgwardt, H. Kling, S. C. Jähnig, and S. Domisch. 2020. From topography to hydrology—The modifiable area unit problem impacts freshwater species distribution models. Ecology and Evolution 10(6):2956–2968.

Katz, L. S. 2024. BridleShinerHabitatModels. GitHub. Available from https://github.com/larakatz/BridleShinerHabitatModels.

Neteler, M., M. H. Bowman, M. Landa, and M. Metz. 2012. GRASS GIS: A multi-purpose open source GIS. Environmental Modelling & Software 31:124–130.

U.S. Geological Survey (USGS). 1998. 3D Elevation Program 10-Meter resolution Digital Elevation Model. Raster, Earth Engine Data Catalog, accessed October 7, 2024, at https://developers.google.com/earth-engine/datasets/catalog/USGS_3DEP_10m.

U.S. Geological Survey (USGS). 2021. USGS Watershed Boundary Dataset (WBD) for 2-digit Hydrologic Unit - 01. Geodatabase, Watershed Boundary Dataset (WBD) - USGS National Map Downloadable Data Collection, ScienceBase, accessed October 7, 2024, at https://www.sciencebase.gov/catalog/item/5a03bdb3e4b0dc0b45b31a81.

**Supplement F. Detailed methods and results for bridle shiner species distribution models.**

## **METHODS**

Because we were using input points and covariates representative of entire drainages, we could not randomly assign pseudo-absences using the *BIOMOD_FormatingData* function. Therefore, we generated 1,000 random seeds and used each seed to select a subset of points to use as pseudo-absences for each of the four period/drainage-type combinations (historical period and HUC12s [Figure 2, Model 2a]; historical period and HUC14s [Model 2b]; current period and HUC12s [Model 3a]; current period and HUC14s [Model 3b]). Each drainage was represented by its centroid point and could only be assigned one value for occupancy (i.e., a drainage could not have both a “*presence*” and a “*pseudo-absence*”).

We followed the recommendations of the *biomod2* pseudo-absence vignette (Thuiller et al. 2024) and assigned three times as many pseudo-absences as known presence points in each model (*n* = 156 Model 2a pseudo-absence points; *n* = 228 Model 2b points; *n* = 129 Model 3a points; *n* = 177 Model 3b points). We weighted presence and pseudo-absence points equally (Barbet-Massin et al. 2012). Once pseudo-absences were assigned, we split each input dataset into 1,000 combinations of model calibration-validation (training) presence points (80%) and ensemble model evaluation presence points (20%; Hastie et al. 2009; Meller et al. 2014). To calibrate individual models, we further split the training data into calibration (75% [60% of entire dataset]) and validation (25% [20% of entire dataset]) datasets with 10 cross-validations. We conducted 10 permutations to estimate variable importance for each model.

Machine learning models have fixed settings, or hyperparameters, that must be defined prior to model training. We used the *biomod2* “bigboss” options as base hyperparameter values for the GBM and RF models and modified the *biomod2* “default” options for the CTA/CART models. The “default” options for CTA models mostly use the *rpart* package default parameter values: we changed the default *minsplit* value (*minsplit* = 5) from *biomod2* to match the relationship between *minsplit* and *minbucket* in *rpart* (*minsplit* = 3**minbucket* = 15). We also changed the number of cross-validations to 100 to obtain consistent estimates of prediction error for each tree (Breiman et al. 1984). We also retained the *rpart* default complexity parameter (*cp*) of 0.01 rather than the default *biomod2 cp* (*cp* = 0.001) because higher *cp* threshold values result in smaller trees and less model overfitting. Machine-learning models are prone to overfitting, and while more complex formulations tend to have better in-sample performance, simpler formulations have greater transferability (Wenger and Olden 2012). We kept all the bigboss hyperparameters for the RF models but left the *mtry* hyperparameter as null rather than define a set number of candidate variables to try at each tree node. This allowed the model to use the *randomForest* package (version 4.7-1.1; Liaw and Wiener 2002) *mtry* default (*mtry* = square root of number of explanatory variables for classification trees).

We evaluated each run of each individual model (*n* = 10,000 CTA runs, *n* = 10,000 GBM runs, and *n* = 10,000 RF runs) using the cross-validated receiver operating characteristic curve metric (ROC; Fielding and Bell 1997) and the true skill statistic (TSS; Allouche et al. 2006) of the validation dataset. ROC values above 0.8 indicate good predictive performance, and values above 0.9 indicate high predictive performance (González-Ferreras et al. 2016; Gu et al. 2024). TSS values above 0.6 indicate good predictive performance, and values above 0.8 indicate high predictive performance (González-Ferreras et al. 2016).

We then generated 1,000 ensemble models using the *BIOMOD_EnsembleModeling* function and the 1,000 random seeds. We used each seed’s respective combined calibration-validation dataset to train that seed’s ensemble model. For each seed, we retained model runs with a TSS value of at least 0.5 (as TSS values below 0.5 suggest poor model performance; González-Ferreras et al. 2016; Gu et al. 2024). We did not generate an ensemble model from seeds where TSS < 0.5 for all model runs or TSS ≥ 0.5 for only one model run. As with the individual models, we conducted 10 permutations to estimate variable importance for each ensemble model. We chose to compute the ensemble weighted mean (*EMwmean*) probability of presence, both the ensemble weighted mean and ensemble committee averaged (*EMca*) variable importance, and to evaluate the ensemble models using ROC and TSS. We used a proportional weighted mean decay so that weights were assigned to models in proportion to their TSS value (Thuiller et al. 2024).

We used the *BIOMOD_EnsembleForecasting* function to predict bridle shiner presence at points within the evaluation dataset and the *pROC* package (version 1.18.5; Robin et al. 2011) to evaluate the model area under the ROC curve (AUC). AUC estimates can be misleading when generating pseudo-absences from more distant areas, but we attempted to avoided this bias when we restricted the model extent to a 50-km buffer around the known historical bridle shiner range (Lobo et al. 2008; Sutton et al. 2015).

Raw probability scores generated by a model need to be rescaled by species prevalence in order to reflect habitat suitability (Jiménez-Valverde and Lobo 2007; Lobo et al. 2008). We selected the threshold coordinates of the AUC curve that maximized both sensitivity and specificity (Jiménez-Valverde and Lobo 2007; Komac et al. 2016), then used these metrics to calculate TSS. If more than one set of coordinates maximized AUC, we saved all threshold coordinate sets and their associated TSS values in the ensemble model output summary.

We then forecast the ensemble models using the entire presence/pseudo-absence dataset (calibration, validation, and evaluation points) and the points with unknown presence (drainages not chosen as pseudo-absences). We used the *matrixStats* package (Version 1.3.0; Bengtsson 2024) to assign weights by TSS value to each ensemble model and calculated the weighted mean probability of presence at each point and for each final ensemble model (Figure 2, Models 2a, 2b, 3a, and 3b). We used the same TSS-based weights to calculate a weighted mean threshold value for each final ensemble model. We considered all values at or below the threshold probability value to be “absences” and all values above the threshold to be “presences.” We could not directly determine the AUC or TSS of the final ensemble model predictions because occupancy status was truly unknown for most of the drainages. Alternately we used the means of the intermediate ensemble model AUC, TSS, sensitivity, specificity, and threshold values to assess final ensemble model performance.

## **RESULTS**

**Table SF1**. Assessment of three types of species distribution model (Classification Tree Analysis [CTA], Generalized Boosted Models [GBM], and Random Forest [RF]) fit to the validation dataset of each time period/spatial scale combination (historical/HUC12 [Figure 2, Model 2a], historical/HUC14 [Model 2b], current/HUC12 [Model 3a], and current/HUC14 [Model 3b]) of Maine and New Hampshire bridle shiner (*Notropis bifrenatus*) presence-pseudo-absence data.

|  | **Model 2a** | | **Model 2b** | | **Model 3a** | | **Model 3b** | | **Overall** | |
| --- | --- | --- | --- | --- | --- | --- | --- | --- | --- | --- |
|  | **ROC** | **TSS** | **ROC** | **TSS** | **ROC** | **TSS** | **ROC** | **TSS** | **ROC** | **TSS** |
| **CTA** | 0.7141 | 0.4142 | 0.6954 | 0.3840 | 0.7468 | 0.4799 | 0.7315 | 0.4472 | 0.7220 | 0.4313 |
| **GBM** | 0.8037 | 0.2596 | 0.7930 | 0.3474 | 0.8343 | 0.3653 | 0.8269 | 0.4152 | 0.8145 | 0.3469 |
| **RF** | 0.7984 | 0.1746 | 0.7970 | 0.2996 | 0.8355 | 0.3156 | 0.8260 | 0.3530 | 0.8142 | 0.2857 |

**Table SF2**. Assessment of 1000 intermediate species distribution ensemble models of the historical (1898-2008) and current (2009-2022) bridle shiner (*Notropis bifrenatus*) range in Maine and New Hampshire using held-apart evaluation datasets of presences and pseudo-absences. Models were fit to the combined calibration-validation dataset for each time period/spatial scale combination: historical/HUC12 (Model 2a), historical/HUC14 (Model 2b), current/HUC12 (Model 3a), and current/HUC14 (Model 3b). Assessment metrics included the mean area under the receiver operating characteristic curve (AUC), mean true skill statistic (TSS), mean sensitivity and specificity, the mean and TSS-weighted mean threshold values, and the number of intermediate ensemble models used to build the final ensemble.

|  | **Model 2a** | **Model 2b** | **Model 3a** | **Model 3b** | **Mean** |
| --- | --- | --- | --- | --- | --- |
| **Mean AUC** | 0.7928 | 0.8279 | 0.7919 | 0.8362 | 0.8122 |
| **Mean TSS** | 0.5991 | 0.6282 | 0.6072 | 0.6254 | 0.6150 |
| **Mean sensitivity** | 0.9301 | 0.9368 | 0.8808 | 0.8720 | 0.9049 |
| **Mean specificity** | 0.6690 | 0.6914 | 0.7264 | 0.7533 | 0.7100 |
| **Mean threshold** | 362.9 | 375.2 | 349.8 | 368.6 | 364.1 |
| **Weighted mean threshold** | 373.7 | 382.4 | 358.5 | 374.2 | 372.2 |
| **Number of models** | 892 | 959 | 897 | 961 | 927 |

**Table SF3**. Number of drainages at the pseudo-Hydrologic Unit Code 14 (HUC14) and HUC12 scale predicted to be historically (1898-2008) and currently (2009-2022) occupied by bridle shiner (*Notropis bifrenatus*) by species distribution models. The “Change” columns denote proportional percent change in drainage number over time.

|  | **Model 2a** | **Model 3a** | **Change (HUC12)** | **Model 2b** | **Model 3b** | **Change**  **(HUC14)** |
| --- | --- | --- | --- | --- | --- | --- |
| **Maine** | 71 | 55 | -20.6% | 267 | 161 | -36.2% |
| **New Hampshire** | 118 | 105 | -9.6% | 357 | 263 | -15.9% |
| **Total*** | 171 | 144 | -14.1% | 595 | 406 | -24.4% |
| *The total number of drainages is less than the sum of drainages in Maine and New Hampshire because multiple drainages overlap the state border. | | | | | | |

## **REFERENCES**

Allouche, O., Tsoar, A., and Kadmon, R. 2006. Assessing the accuracy of species distribution models: prevalence, kappa and the true skill statistic (TSS). Journal of Applied Ecology 43(6): 1223–1232. doi:10.1111/j.1365-2664.2006.01214.x.

Barbet-Massin, M., Jiguet, F., Albert, C., & Thuiller, W. 2012. Selecting pseudo-absences for species distribution models: How, where and how many? Methods in Ecology and Evolution, 3, 327–338. https://doi.org/10.1111/j.2041-210X.2011.00172.x

Bengtsson, H. 2024. *matrixStats*: Functions that apply to rows and columns of matrices (and to vectors). R. Available from https://CRAN.R-project.org/package=matrixStats.

Breiman, L., Friedman, J., Olshen, R. A., & Stone, C. J. 1984. Classification and regression trees. Chapman and Hall/CRC. https://doi.org/10.1201/9781315139470.

Fielding, A.H., and Bell, J.F. 1997. A review of methods for the assessment of prediction errors

in conservation presence/absence models. Environmental Conservation 24(1): 38–49. Cambridge University Press. doi:10.1017/S0376892997000088.

González-Ferreras, A.M., Barquín, J., and Peñas, F.J. 2016. Integration of habitat models to

predict fish distributions in several watersheds of Northern Spain. Journal of Applied Ichthyology 32(1): 204–216. doi:10.1111/jai.13024.

Gu, R., Wei, S., Li, J., Zheng, S., Li, Z., Liu, G., and Fan, S. 2024. Predicting the impacts of

climate change on the geographic distribution of moso bamboo in China based on *biomod2* model. Eur J Forest Res. doi:10.1007/s10342-024-01706-9.

Hastie, T., Tibshirani, R., and Friedman, J.H. 2009. The elements of statistical learning: Data mining, inference, and prediction. *In* 2nd edition. Springer, New York, NY.

Jiménez-Valverde, A., and Lobo, J.M. 2007. Threshold criteria for conversion of probability of

species presence to either-or presence-absence. Acta Oecologica 31(3): 361–369. doi:10.1016/j.actao.2007.02.001.

Komac, B., Esteban, P., Trapero, L., and Caritg, R. 2016. Modelization of the current and future

habitat suitability of *Rhododendron ferrugineum* using potential snow accumulation. PloS one 11: e0147324. doi:10.1371/journal.pone.0147324.

Liaw, A., and Wiener, M. 2002. Classiﬁcation and regression by randomForest. R News 2: 18-

22.

Lobo, J.M., Jiménez-Valverde, A., and Real, R. 2008. AUC: A misleading measure of the

performance of predictive distribution models. Global Ecology and Biogeography 17(2): 145–151. doi:10.1111/j.1466-8238.2007.00358.x.

Meller, L., Cabeza, M., Pironon, S., Barbet-Massin, M., Maiorano, L., Georges, D., and Thuiller,

W. 2014. Ensemble distribution models in conservation prioritization: From consensus predictions to consensus reserve networks. Diversity and Distributions 20(3): 309–321. doi:10.1111/ddi.12162.

Robin, X., Turck, N., Hainard, A., Tiberti, N., Lisacek, F., Sanchez, J.-C., and Müller, M. 2011.

*pROC*: An open-source package for R and S+ to analyze and compare ROC curves. BMC Bioinformatics 12(1): 77. doi:10.1186/1471-2105-12-77.

Sutton, W.B., Barrett, K., Moody, A.T., Loftin, C.S., DeMaynadier, P.G., and Nanjappa, P.

2015. Predicted changes in climatic niche and climate refugia of conservation priority salamander species in the Northeastern United States. Forests 6(1): 1–26. Multidisciplinary Digital Publishing Institute. doi:10.3390/f6010001.

Thuiller, W., Georges, D., Gueguen, M., Engler, R., Breiner, F., Lafourcade, B., Patin, R., and

Blancheteau, H. 2024. *biomod2*: Ensemble platform for species distribution modeling. R. Available from https://biomodhub.github.io/biomod2/.

Wenger, S.J., and Olden, J.D. 2012. Assessing transferability of ecological models: an underappreciated aspect of statistical validation. Methods in Ecology and Evolution **3**(2): 260–267. doi:10.1111/j.2041-210X.2011.00170.x.

# **Supporting Information: All References**

Allouche, O., Tsoar, A., and Kadmon, R. 2006. Assessing the accuracy of species distribution models: prevalence, kappa and the true skill statistic (TSS). Journal of Applied Ecology 43(6): 1223–1232. doi:10.1111/j.1365-2664.2006.01214.x.

Barbet-Massin, M., Jiguet, F., Albert, C., & Thuiller, W. 2012. Selecting pseudo-absences for species distribution models: How, where and how many? Methods in Ecology and Evolution, 3, 327–338. https://doi.org/10.1111/j.2041-210X.2011.00172.x

Bengtsson, H. 2024. *matrixStats*: Functions that apply to rows and columns of matrices (and to vectors). R. Available from https://CRAN.R-project.org/package=matrixStats.

Bivand, R. S. 2024. *rgrass*: Interface between “GRASS” Geographical Information System and “R”. R.

Breiman, L., Friedman, J., Olshen, R. A., & Stone, C. J. 1984. Classification and regression trees. Chapman and Hall/CRC. https://doi.org/10.1201/9781315139470

Cooper, G. P. 1939. A biological survey of thirty-one lakes and ponds of the Upper Saco River and Sebago Lake drainage systems in Maine. Maine Department of Inland Fisheries and Game, Fish Survey Report 2. Available from https://digitalmaine.com/ifw_docs/70/ [accessed March 2021].

Doering, P. H., C. T. Roman, L. L. Beatty, A. A. Keller, and C. A. Oviatt. 1995. Water quality and habitat evaluation of Bass Harbor Marsh Acadia National Park, Maine. Page 213. National Park Service, New England System Support Office (NESO), NPS/NESORNR/NRTR/95-31, Boston, MA. Available from https://openparksnetwork.org/single-item-view/?oid=OPN_NS:E413FC0BFEFB4D4F437728EC961791BC#page/1/mode/2up [accessed February 2021].

Fielding, A.H., and Bell, J.F. 1997. A review of methods for the assessment of prediction errors

in conservation presence/absence models. Environmental Conservation 24(1): 38–49. Cambridge University Press. doi:10.1017/S0376892997000088.

Friedrichs‐Manthey, M., S. D. Langhans, T. Hein, F. Borgwardt, H. Kling, S. C. Jähnig, and S. Domisch. 2020. From topography to hydrology—The modifiable area unit problem impacts freshwater species distribution models. Ecology and Evolution 10(6):2956–2968.

González-Ferreras, A.M., Barquín, J., and Peñas, F.J. 2016. Integration of habitat models to

predict fish distributions in several watersheds of Northern Spain. Journal of Applied Ichthyology 32(1): 204–216. doi:10.1111/jai.13024.

Gu, R., Wei, S., Li, J., Zheng, S., Li, Z., Liu, G., and Fan, S. 2024. Predicting the impacts of

climate change on the geographic distribution of moso bamboo in China based on *biomod2* model. Eur J Forest Res. doi:10.1007/s10342-024-01706-9.

Harrington, R. W. 1947b. The early life history of the bridled shiner, *Notropis bifrenatus* (Cope). Copeia 1947(2):97–102.

Harrington, R. W. 1948a. The life cycle and fertility of the bridled shiner, *Notropis bifrenatus* (Cope). The American Midland Naturalist 39(1):83–92.

Hastie, T., Tibshirani, R., and Friedman, J.H. 2009. The elements of statistical learning: Data mining, inference, and prediction. *In* 2nd edition. Springer, New York, NY.

Jiménez-Valverde, A., and Lobo, J.M. 2007. Threshold criteria for conversion of probability of

species presence to either-or presence-absence. Acta Oecologica 31(3): 361–369. doi:10.1016/j.actao.2007.02.001.

Katz, L. S. 2024. BridleShinerHabitatModels. GitHub. Available from https://github.com/larakatz/BridleShinerHabitatModels.

Kendall, W.C. 1914. An annotated catalogue of the fishes of Maine. Portland Society of Natural History, Portland, ME. Available from https://www.biodiversitylibrary.org/item/45961.

Komac, B., Esteban, P., Trapero, L., and Caritg, R. 2016. Modelization of the current and future

habitat suitability of *Rhododendron ferrugineum* using potential snow accumulation. PloS one 11: e0147324. doi:10.1371/journal.pone.0147324.

Liaw, A., and Wiener, M. 2002. Classiﬁcation and regression by randomForest. R News 2: 18-

22.

Lobo, J.M., Jiménez-Valverde, A., and Real, R. 2008. AUC: A misleading measure of the

performance of predictive distribution models. Global Ecology and Biogeography 17(2): 145–151. doi:10.1111/j.1466-8238.2007.00358.x.

Meller, L., Cabeza, M., Pironon, S., Barbet-Massin, M., Maiorano, L., Georges, D., and Thuiller,

W. 2014. Ensemble distribution models in conservation prioritization: from consensus predictions to consensus reserve networks. Diversity and Distributions 20(3): 309–321. doi:10.1111/ddi.12162.

Neteler, M., M. H. Bowman, M. Landa, and M. Metz. 2012. GRASS GIS: A multi-purpose open source GIS. Environmental Modelling & Software 31:124–130.

New Hampshire Fish and Game Dept. (NHFGD). (n.d.). New Hampshire fish survey map. Available from https://nhfg.maps.arcgis.com/apps/MapJournal/index.html?appid=d6549e90155b441fa0e29bdc44eebc2b# [accessed 25 February 2025].

New Hampshire Fish and Game Dept. (NHFGD). 2015. New Hampshire Wildlife Action Plan. New Hampshire Fish and Game Department, SWG Report, Concord, NH. Available from https://www.wildlife.nh.gov/wildlife-and-habitat/nh-wildlife-action-plan/swap-2015 [accessed May 2023].

North American Native Fishes Association. 2023. Bridle shiner—*Notropis bifrenatus*. Available from https://fishmap.org/species/Bridle-Shiner.html [accessed January 2023].

Robin, X., Turck, N., Hainard, A., Tiberti, N., Lisacek, F., Sanchez, J.-C., and Müller, M. 2011.

*pROC*: An open-source package for R and S+ to analyze and compare ROC curves. BMC Bioinformatics 12(1): 77. doi:10.1186/1471-2105-12-77.

Sutton, W.B., Barrett, K., Moody, A.T., Loftin, C.S., DeMaynadier, P.G., and Nanjappa, P.

2015. Predicted changes in climatic niche and climate refugia of conservation priority salamander species in the Northeastern United States. Forests 6(1): 1–26. Multidisciplinary Digital Publishing Institute. doi:10.3390/f6010001.

Thuiller, W., Georges, D., Gueguen, M., Engler, R., Breiner, F., Lafourcade, B., Patin, R., and

Blancheteau, H. 2024. *biomod2*: Ensemble platform for species distribution modeling. R. Available from https://biomodhub.github.io/biomod2/.

U.S. Environmental Protection Agency (USEPA). 2016. Region 1 Maine lakes data sets. https://archive.epa.gov/emap/archive-emap/web/html/index-158.html.

U.S. Geological Survey (USGS). 1998. 3D Elevation Program 10-Meter resolution Digital Elevation Model. Raster, Earth Engine Data Catalog, accessed October 7, 2024, at https://developers.google.com/earth-engine/datasets/catalog/USGS_3DEP_10m.

U.S. Geological Survey (USGS). 2021. USGS Watershed Boundary Dataset (WBD) for 2-digit Hydrologic Unit - 01. Geodatabase, Watershed Boundary Dataset (WBD) - USGS National Map Downloadable Data Collection, ScienceBase, accessed October 7, 2024, at https://www.sciencebase.gov/catalog/item/5a03bdb3e4b0dc0b45b31a81.

Vaux, P. D. 2013. Maine Aquatic Biodiversity Project Database [Dataset]. Available from https://www.gulfofmaine.org/kb/2.0/record.html?recordid=9790 [accessed September 2023].

Wenger, S.J., and Olden, J.D. 2012. Assessing transferability of ecological models: an underappreciated aspect of statistical validation. Methods in Ecology and Evolution **3**(2): 260–267. doi:10.1111/j.2041-210X.2011.00170.x.
